# Sampling informational properties of codon usage through the tree of life

**DOI:** 10.1371/journal.pone.0335824

**Published:** 2025-11-26

**Authors:** Octavio Martínez, Manuel Humberto Reyes-Valdés, Neftalí Ochoa-Alejo

**Affiliations:** 1 Unidad de Genómica Avanzada (UGA), Cinvestav, Irapuato, Guanajuato, México; 2 Department of Plant Breeding, Universidad Autónoma Agraria Antonio Narro, Saltillo, Coahuila, México; 3 Departamento de Ingeniería Genética, Unidad Irapuato, Cinvestav, Irapuato, Guanajuato, México; Shandong Agricultural University, CHINA

## Abstract

The genetic code, a unifying principle in biology, ensures that all organisms, stemming from a Last Universal Common Ancestor (LUCA), share fundamental rules for translating DNA into proteins. However, codon usage varies across the tree of life, influenced not only by GC-content and proteome composition but also by complex, often less understood rules dependent on each species’ evolutionary trajectory. To better understand these rules, we segregated codons into their functional parts and applied Shannon’s information-theoretic measures to 1,434 species from eight diverse taxonomic groups. We provide robust evidence that the first codon base plays a central role in amino acid determination, while the third base serves an accessory function. Using conditional entropy measures, we rigorously quantified this relationship, universally confirming the greater informational variability of the third base across all sampled species for the first time at this scale. Our analysis revealed significant heterogeneity in coding strategies across different taxonomic groups. Notably, the unique variability observed in Archaea, in contrast to the more constrained patterns in Eukaryotes and Bacteria, underscores the profound influence of evolutionary pressures and distinct life histories on genetic information processing. The identification of outlier species, exhibiting distinct informational profiles, highlights specific instances where unusual lifestyles or ecological niches may have driven unique adaptations in codon usage and underlying informational dependencies. These informational patterns offer a complementary perspective to traditional phylogenetic analyses, further revealing a hierarchical organization of informational dependencies among codon components that sheds light on the intricate grammar of genetic information. We also rigorously investigated the relationship between GC-content and our informational measures, concluding that these entropy measures provide valuable insights that cannot be obtained from GC-content alone. This work not only offers a novel framework for quantifying informational properties of codon usage but also reveals previously unappreciated aspects of how genetic information is encoded and processed across life’s domains.

## Introduction

The Genetic Code, which dictates the translation of genomic DNA base sequences into the amino acid sequences of proteins, stands as a fundamental unifying principle of biology. Its near universality across all known life forms strongly implies a single common ancestor for all life on Earth [1].

However, codon usage in both nuclear and plastid versions of the genetic code varies–a phenomenon known as “codon bias”. This bias is often studied within restricted taxa, as exemplified by [2], who suggested that in a macroalgal chloroplast, natural selection primarily determines codon bias and correlates with gene expression levels.

It is generally accepted that selection acts on codon usage, optimizing multiple, partially understood signals [3]. In the same paper, the authors demonstrate how AI methods can predict codon sequences from amino acid sequences in various species, showing that their models achieve higher prediction accuracy for highly expressed genes and for bacteria compared to eukaryotes. In a commentary on [3], Elazara et al. [4] note that orthologous coding sequences encode a rich evolutionary history, akin to the etymology of words across human languages. This perspective suggests that species utilize the genetic code with distinct “accents”, and a deeper understanding of these differences will likely enlighten the evolutionary pathways followed by various groups of organisms.

Shannon’s mathematical theory of communication [5] has provided a powerful framework for diverse applications in biology. Its utility for analyzing biological sequences, particularly patterns of codon usage, became especially prominent from the early 1990s. In his foundational paper, Frank Wright [6] defined the Effective Number of Codons (ENc). This measurement, while not explicitly using Shannon’s entropy formula, is inherently an entropy-like quantity, quantifying the departure from a uniform probability distribution of codon usage, i.e., from the maximal possible entropy.

In an analogous way, the “effective number of loci” in quantitative genetics [7] and the “effective number of species” in ecological diversity studies [8] are also entropy-based measurements.

Our research group has utilized Shannon’s information theory to estimate transcriptome diversity and to develop indices for gene specificity and transcriptome specialization, demonstrating these methods using human data [9]. We further demonstrated that cancer reduces transcriptome specialization in humans [10], and have also applied these methods to study *Capsicum* transcriptome dynamics across different accessions during fruit development [11].

Among the most important conceptual and technical advances in the application of Shannon’s information theory to biology are those stemming from the work of Christoph Adami. For example, in his seminal paper [12], Adami clarifies the crucial distinction between “entropy” and “information”, presenting clear applications within molecular biology and genomics. Furthermore, in an opinion piece [13], he convincingly argues that only differences between uncertainties can be considered as “information”, emphasizing that a proper understanding of information in terms of prediction is a key concept in biology. Additionally, in his recent book [14], Adami provides deep and compelling arguments explaining how the evolutionary process creates complexity from information.

Information theory has also been applied to explore the data transmission process inherent in DNA, with the aim that insights from natural methods can inspire the design of improved engineering transmission techniques [15].

The aim of this work is to improve our understanding of the molecular grammar implicit in the nuclear genetic code. To this end, we consider the codon’s functional constituents (individual base positions, duplets, the complete codon, and the encoded amino acid), and apply entropy formulas to dissect average uncertainties and information content. Genomic codon frequencies from 1,434 species across diverse life domains were collected. These data, along with functions for their processing, have been incorporated into a publicly available R package. Our results include a detailed analysis of average uncertainties, confirming the functional roles of different codon parts, estimating variation across diverse taxa, and identifying species with highly distinctive entropy profiles.

## Materials and methods

### Codon frequencies data

We selected species for this study based on the availability of curated codon frequency data within the Codon Statistics Database [16], which contains frequency tables for over 15,000 species. This database was generated from species with reference genomes in the RefSeq database (release 207), by tabulating codon frequencies from the coding sequences (CDSs) of all non-redundant genes (for further details, see [16]). For our analysis, we exclusively used data from nuclear coding genes for each selected species (see S1 Dataset).

Given the potential for overly detailed or confusing interpretations with the tools developed in this study, we decided to analyze a representative sample of species from eight distinct domains of life. Table 1 outlines these groups and the number of species included from each.

**Table 1:** Groups of species included in this study.

Group	Abbreviation	Key	Number
Animal	Animal	An	652
Archaea	Archaea	Ar	435
Enterobacteriaceae	EnteroBac	Eb	126
Plant	Plant	Pl	122
Fungus	Fungus	Fu	71
Virus	Virus	Vi	21
Protist	Protist	Pr	4
Other Bacteria	OtherBac	Ob	3

The total number of species included in this study is 1,434. Each group is identified by its “**Abbreviation**” and “**Key**”, which are consistently used throughout the figures and tables. Within each group, individual species are uniquely labeled by their respective key followed by a sequential number. The full scientific name for each species is available within the R package “Shannon.codon” [17] and also in S1 Dataset.

As presented in Table 1, the number of species analyzed per group varies significantly, ranging from small numbers, such as 3 for Ob (Other Bacteria) or 4 for Pr (Protists, represented by amoebas), to substantially represented groups like Animals (652) and Archaea (435) (see also S1 Table S2 Text). Despite this variability, the total number of species studied here, while extensive for this type of analysis, still represents only a fraction of the vast diversity of life. In contrast, studies of codon frequency data in the literature are often restricted to narrow taxa; thus, the sample employed here offers the advantage of examining the “whole forest” rather than merely “restricted parcels”.

### Dissecting codon functionality

The nuclear genetic code exhibits intra-codon functional characteristics due to its redundancy (or “degeneracy”), where 64 codons code for only 20 amino acids plus a stop signal [1]. An analogy can be drawn with the etymology of spoken words, where an initial part often serves as the root while a latter part specifies; for example, the prefix “Bio-” in “Biogeography” or “Biomolecule.” In an analogous way, the first base within a codon is more determinant than the third base regarding the encoded amino acid (“aa”). Hereafter, the abbreviation “aa” will be used to refer not only to the 20 amino acids but also to the stop signal (encoded by three codons), encompassing all 21 distinct signals within the core genetic code.

To illustrate the differential importance of each base within the codon, consider that, for example, when the first base is G, the 16 codons beginning with that base can code for one of only five different aa (Ala, Asp, Glu, Gly, or Val). In contrast, the 16 codons where the third base is G can code for a total of 14 different aa. Assuming equal frequency for each codon (an artificial but illustrative assumption), the third base is, on average, 2.375 times less determinant than the first base for aa codification (see S5 Table in S2 Text).

To comprehensively analyze codon frequencies, we looked at the functional parts within each codon, beyond just the codon itself and its coded amino acid. We represented each codon as “**FST**”, where “**F**”,“**S**” and “**T**” denote the first, second, and third bases, respectively. This approach allows us to consider four instances for each base position, plus 16 instances for each of the three possible duplets within a codon (i.e., “**FS**”, “**FT**” and “**ST**”).

In this way, we can separately analyze all functionally distinct data for each species. It is important to note that all strata within codons are derived by summing the original frequencies of the relevant codons within each species. For example, to obtain the 16 frequencies of the “**FS**” duplet for a given species, we sum the frequencies of the codons that contain each specific duplet instance.

### Entropies and multivariate spaces from the data

The core equation in Shannon’s communication theory [5] is given by

$$H(X)=-\sum_{i=1}^{i=k}p_{i}\log_{2}(p_{i})$$ (1)

where *X* is a collection of *k* events with probabilities of occurrence $P[X=x_{i}]=p_{i}$ for i=1,2,⋯,k. This equation quantifies the average uncertainty, or **entropy**, associated with this collection of events.

In our context, we consider various collections of events (*X*) for each species, such as the 64 codons, the 21 aa, the three different duplets, or each base within the codon. Furthermore, Eq (1) can be extended to calculate joint average uncertainty for two sets of events (e.g., H(X,Y)), conditional entropies (e.g., H(X|Y)), or mutual information terms (e.g., I(X;Y)).

Our publicly available R package, “Shannon.codon” [17], provides functions to directly calculate all *H* values for each species from their raw codon frequencies, alongside other analytical tools. The package documentation includes definitions for entropy, joint and conditional entropy, and mutual information, all explained within the context of codon frequency analysis, as well as a guide for their interpretation.

One of the primary challenges in interpreting these kinds of results is their inherent multi-dimensionality. For instance, the space generated from the relative frequencies of codons has 64 dimensions, while the space generated by the relative frequencies of amino acids (aa) has 21. While various numerical methods exist to visualize and compare points in such spaces, we chose to estimate dendrograms (tree-like diagrams) from Euclidean distances and by the “complete linkage” clustering method. For studying these dendrograms, we utilized functionalities available in our publicly available R package DendroLikeness [18].

Our study combines hypothesis-driven approaches, where propositions suggested by the informational structure of the genetic code are tested, with exploratory analyses aimed at estimating differences between groups or detecting particularly interesting species within the spaces defined by the methods presented. Detailed results are presented in S2 Text.

A critical consideration in our study is the nature of the input data. Our analyses are based solely on the vector of 64 codon frequencies calculated for each species from its complete set of coding sequences (CDSs). This methodological choice means that all subsequent informational measures, such as entropies and mutual information, are derived from this compressed representation of the genomic data. We acknowledge that this approach inherently discards the sequence-specific information that is critical for traditional phylogenetic analysis and the reconstruction of evolutionary relationships. Our objective, however, was not to replicate a phylogenetic tree but to provide a complementary perspective by quantifying the emergent, high-level informational properties of the genetic code itself. By focusing on the statistical properties of codon usage, our methodology allows us to isolate and explore the fundamental “molecular grammar” of genetic encoding, revealing underlying rules and dependencies that may be obscured in sequence-based analyses. Therefore, while our measures are not intended to capture an organism’s full evolutionary history, they are uniquely suited to our goal of understanding the informational structure of the genetic code across the tree of life.

## Results and discussion

### Analysis of main entropy measures per group of species

Given the dissection of the codon into its functional parts, we identified eight primary sources of average uncertainty (entropy), which are presented in Table 2.

**Table 2:** Sources of variation, instances and associated *H*’s for codon partition.

Source	Instances	*k*	*H*’s	max(H)
Codon	codon	64	*H*(codons)	$\log_{2}(64)=6$
Amino acid	*aa*	21	*H*(*aa*)	$\log_{2}(21)\approx 4.3923$
Duplets	FS, FT, ST	16	H(FS), H(FT), H(ST)	$\log_{2}(16)=4$
Bases	F, S, T	4	H(F), H(S), H(T)	$\log_{2}(4)=2$

Column “**Source**” gives the part of the codon, “**Instances**” are the possible parts, “*k*” gives the number of classes (probabilities) employed, “*H* **’s**” give the name of the *H*’s considered, which are calculated by modifying Eq (1) employing the corresponding *k* probabilities, and, finally, “max(H)" gives the maximum possible value of the terms (in bits). For each one of the 1,434 species the values are calculated with the R package“Shannon.codon” [17] (see S2 Text).

To mitigate the influence of outliers, we calculated the median rather than the mean for each of the eight entropy terms (*H*) listed in Table 2. A comprehensive overview, including the minimum and maximum median values for each of the eight species groups (from Table 1), and each of the eight entropy terms (from Table 2), is available in S3 Table in S2 Text.

For clearer interpretation, we will focus on the maximum percentages of the *H* values relative to their maximum possible value (as shown in the corresponding column in Table 2). These percentages indicate how closely the observed values approach the maximum efficiency in the use of the corresponding genetic coding.

Across all 1,434 species, we observed the highest *H* value for bases for the second base within the codon, *H*(*S*), at 98.71%. This represents the value of *H* closest to 100%. The first base within the codon, *H*(*F*), followed closely with an efficiency of 98.10%. It is interesting to note that the second base’s *H* value is slightly higher (0.61%) than that of the first base, which generally plays a more significant role in determining the amino acid. This slight difference could be attributed to a bias in the sampled species, or perhaps because the second base has more degrees of freedom within codons, given the necessity to code for specific amino acids.

Conversely, the efficiency for amino acids, measured as *H*(*aa*) across all species, was 95.30%. This surprisingly ranked as the lowest among the eight *H* values. While this indicates that the ultimate goal of codons-coding for amino acids is near maximum efficiency in the sampled organisms, it is still lower than the efficiencies observed for the other seven terms. This includes the efficiency of codons, *H*(codon), which stands at 95.68% –higher than that of amino acids, but still less than the other six terms that dissect codon parts.

Looking at the maximum percentages per *H* term and per group of species in S3 Table (S2 Text), we observe intriguing differences. For *H*(codon), plants achieve the highest efficiency within a group, reaching 96.52%. In contrast, animals demonstrate the maximum efficiency for *H*(*aa*) at 95.54%. Animals also exhibit the highest efficiency for the FS duplet, with an *H*(*FS*) value of 97.66%. Meanwhile, plants again show the maxima for the FT and ST duplets, at 98.12% and 97.76%, respectively. Considering individual bases per codon position, the maximum *H*(*F*) (98.37%) is found in plants, the maximum *H*(*S*) (98.95%) in archaea, and the maximum *H*(*T*) (98.78%) in viruses. These variations are likely attributable to the distinct selective pressures and evolutionary pathways characterizing each taxon. Interestingly, within the eukaryotic groups, both animals and plants display the highest coding efficiencies for codons, amino acids, and all three duplets, suggesting that these groups utilize their genomic coding potential almost perfectly.

### Groups in the *H* and codon frequency spaces

Interpreting extremely large dendrograms, like one showing all 1,434 species in our study, would be quite challenging. Instead, we can calculate Euclidean distances between the eight groups of species, which allows us to present dendrograms illustrating the relative similarities among these groups (see S2 Sect in S2 Text).

**Fig 1:**
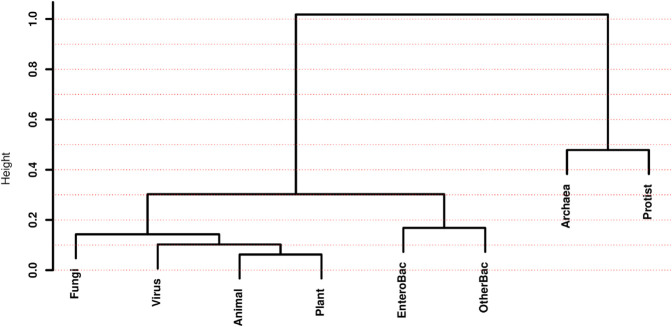
Dendrograms for species groups in the space of the eight main *H* measures.

**Fig 2:**
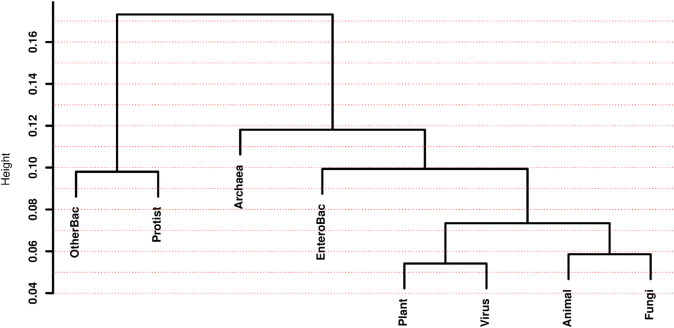
Dendrograms for species groups in the space of the 64 RCF.

For this analysis, we considered two multivariate spaces. In the first, each species is represented by a vector of its eight main *H* measures (from the “*H* **’s**” column in Table 2). In the second, each species is represented by a vector of its 64 Relative Codon Frequencies –hereafter abbreviated by the acronym “RCF”. In both scenarios, we calculated medians per group and constructed dendrograms using Euclidean distances between groups, employing the compact method. Figs 1 and 2 display the resulting dendrograms for each of these two spaces, respectively.

The dendrograms in Figs 1 and 2 are strikingly different. Beyond the variations in height, which simply reflect the distinct scales of the two spaces –the *H* measures in Fig 1 and the RCF in Fig 2– the two dendrograms share none of the seven clusters of species groups. This implies that the two spaces possess very distinctive distance structures, at least for the group medians, leading to dissimilar relatedness panoramas between groups.

In Fig 1 the two most similar groups in the *H* space are animals and plants, forming a cluster with the lowest intra-group distance. In contrast, archaea and protists form a binary cluster at a height of almost 0.5 in this space, indicating that they are the two most distant groups from the remaining six groups. Another well-separated binary cluster consists of organisms classified as bacteria (“EnteroBac” and “OtherBac” in Table 1).

Conversely, in Fig 2, the binary cluster with the lowest height is formed by plants and viruses. Surprisingly, the binary cluster with the highest height comprises the small set of “OtherBac” and protists, represented by four amoeba species.

While the dendrograms in Figs 1 and 2 are clearly not phylogenetic trees, it is instructive to broadly compare them with phylogenetic trees constructed from molecular data. In these comparisons, we can exclude the virus group, which is generally considered highly distinct and often shown interacting with, rather than being part of, the main tree of cellular life. In their seminal work, Carl Woese and his colleagues [19] proposed the three-domain system (Archaea, Bacteria, Eukarya), the most widely accepted high-level classification of life, originally based on rRNA sequences. In that work, the authors present a phylogenetic tree for 19 organisms from the three domains, showing that Archaea and Eukarya are more closely related to each other than to Bacteria, with Bacteria closer to the tree’s root. Neither of the two dendrograms in Figs 1 or 2 is fully consistent with the tree presented in [19]. This demonstrates that distance measures in the *H* or codon frequency spaces don’t fully recapitulate phylogenetic relations –and it would be naive to expect them to, given the complex factors influencing evolutionary pathways. A more recent study by [20] offers a highly comprehensive phylogenetic tree, incorporating massive genomic data that have expanded our understanding of microbial diversity, especially within Bacteria and Archaea, and how Eukarya emerged from within Archaea, including major eukaryotic supergroups (Animals, Plants, Fungi, Protists) as distinct clades. Again, neither of the two dendrograms in Figs 1 or 2 is completely coherent with the one presented in [20].

From Figs 1 and 2 we have seen that neither the *H* measures nor the RCF alone allow for a fully consistent reconstruction of the groups’ phylogeny. However, we will now explore how studying the extended *H* space, which comprises the 54 non-redundant entropy measures (see S10 Table in S2 Text), allows us to test hypotheses derived from the genetic code’s informational structure. This approach could significantly improve our understanding of the molecular grammar used by living organisms.

### Is the entropy in the third base larger than in the first one?

Since the elucidation of the DNA structure in 1953 [21], it was clear that A/T and G/C base pairing would be fundamental to molecular replication, making it ideal for genetic coding. Subsequently, within the “*RNA Tie Club*” |founded by George Gamow| speculation began regarding how the sequence of DNA bases could encode the sequence of the 20 amino acids. The first experimental evidence for the genetic code was presented almost a decade later, in 1961 [22]. Soon after, in a purely theoretical work, Francis Crick postulated the wobble hypothesis [23]. This hypothesis suggested that while standard base pairs might be used strictly in the first two positions of the triplet (codon), some *wobble* could occur in the pairing of the third base, thereby explaining the degeneracy of the genetic code.

With our current comprehensive understanding of the nuclear genetic code, we know that 18 of the 20 amino acids are encoded by two to six synonymous codons; methionine (Met) and tryptophan (Trp) are the only exceptions, each being coded by a single codon. Furthermore, the importance of each base within a codon for amino acid coding exhibits a decreasing gradient from the first to the third position (see S5 Table in S2 Text, which details the amino acids coded by different bases in the first and third codon positions). It is well established that more mutations are tolerated in the third codon position than in the first, precisely because a change at the third position is less likely to alter the encoded amino acid [24].

This observation leads us to hypothesize that the entropy in the third base is greater than that in the first, mathematically expressed as H(T)>H(F). Given that entropy (*H*) quantifies average uncertainty, it seems reasonable to infer that the third base, possessing more freedom to vary without changing the encoded amino acid, will consequently exhibit higher entropy than the first.

Table 3 presents the counts and percentages for which the hypothesis H(T)>H(F) holds true across each species group and for the entire set of 1,434 species (last column).

**Table 3:** Numbers and percentages in which the hypothesis H(T)>H(F) was true per group.

Animal	Archaea	EnteroBac	Plant	Fungus	Virus	Protist	OtherBac	Total
415/652	117/435	57/126	72/122	35/71	12/21	1/4	3/3	712/1,434
(64)	(27)	(45)	(59)	(49)	(57)	(25)	(100)	(50)

Column labels are the abbreviations of the groups in Table 1. The first row gives the quotients of the number of cases in which the hypothesis H(T)>H(F) was true over the total number of species per group. The second row presents between parenthesis the rounded percentages of the cases in which the hypothesis H(T)>H(F) was true. (see also S6 Table in S2 Text).

From Table 3, we observe that H(T)>H(F) is true for approximately half of the 1,434 species, specifically in a proportion of 712/1,434≈0.4965. However, there is considerable heterogeneity among groups in the percentages for which this hypothesis holds. Considering only groups represented by at least 70 species (Animals, Archaea, EnteroBac, Plants, and Fungi), we find that in the eukaryotic groups, Animals and Plants, over half of the evaluated species, 64% and 59%, respectively, exhibit higher entropy in the third codon base than in the first. Fungi are borderline, with 49%. In contrast, Archaea show the lowest percentage, with only 27% of species supporting the hypothesis, close to just one in four. For the other large prokaryotic group, EnteroBac, this percentage is closer to half, at 45%.

In S3.1 Sect of S2 Text, we further detail the parameter *H*(*T*)–*H*(*F*), which is greater than zero in a given species only when H(T)>H(F). The median of this parameter is greater than zero exclusively for Animals and Plants, confirming that the hypothesis H(T)>H(F) is typically met only in these two groups. Furthermore, the distributions of *H*(*T*)–*H*(*F*) values per group, shown in S4 Fig within S2 Text, reveal that Archaea exhibit considerable variation, while Animals and Plants display very compact distributions.

We observed (Table 3) that the hypothesis of a larger *H* value in the third codon base compared to the first is supported in only about 50% of all sampled species. One potential reason for this relatively low percentage could be that our analysis does not account for differences in the amino acid composition across the genomes of the sampled species. Mueller et al. [25] globally compared proteomes across the major domains of life using experimental proteomics data. In their work, these authors demonstrated a clear separation among Archaea, Eukaryotes, and Bacteria within the space of experimentally validated proteomes from 100 species (9 archaea, 49 bacteria, and 32 eukaryotes). This finding led us to conclude that we should discount the effects of amino acid composition from the entropies of the third and first bases to enable a fairer test of our hypothesis.

### Taking into account amino acids when evaluating base entropy

Conditional entropy [5] quantifies the average uncertainty remaining in a signal after a related signal has been observed. For two sets of events, *X* and *Y*, the conditional entropy of *X* given *Y*, H(X|Y), is defined as H(X|Y)=H(X,Y)−H(Y). This means the conditional entropy of *X* given *Y* is the joint entropy of the two events minus the entropy of the known event (For a more detailed explanation, please refer to the file “IR2025ShannonInfo.pdf” within the “Shannon.codon” [17] package).

In our specific context, for each sampled species, we are interested in the conditional uncertainty inherent in the bases given the amino acids. Specifically, we can measure H(T|aa) and H(F|aa), which represent the conditional entropies of the third and first bases, respectively, when the corresponding amino acid (*aa*) is known.

Using conditional entropies, the hypothesis that the third base is more variable than the first when the effect of amino acids is discounted is mathematically translated to H(T|aa)>H(F|aa). As before, this hypothesis is grounded in the fact that the first base within the codon plays a more critical role in amino acid determination than the third. This is because variation in the third base is often irrelevant for the ultimate goal of genetic coding: amino acid determination.

The difference, Diff=H(T|aa)−H(F|aa), serves as a proxy to evaluate the hypothesis H(T|aa)>H(F|aa). A value of Diff>0 implies a larger average uncertainty in the third base compared to the first, with the effects of amino acids discounted in both cases.

**Fig 3:**
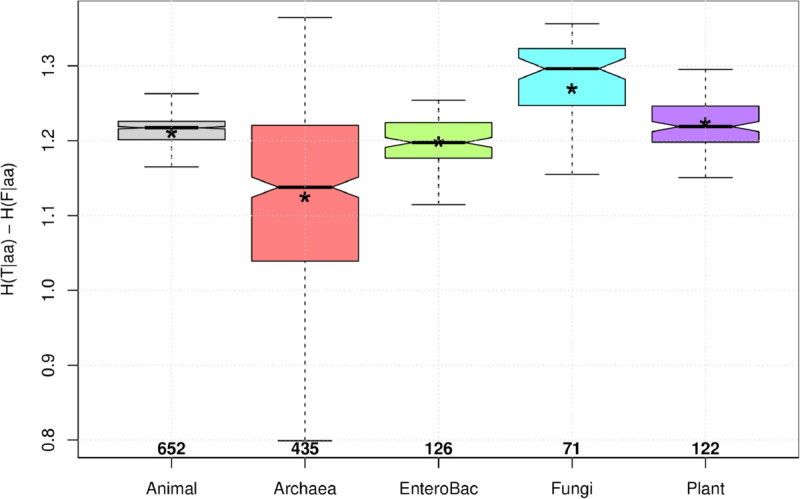
Distributions as box plots of the values of Diff=H(T|aa)−H(F|aa) for five groups of species. Numbers at the bottom of each box plot are the number of species in the corresponding groups. Asterisks mark the position of the mean for each distribution.

Notably, in all 1,434 species studied here, the hypothesis H(T|aa)>H(F|aa) was confirmed. S7 Table in S2 Text presents the main statistics for the difference Diff=H(T|aa)−H(F|aa) for each of the eight species groups, while Fig 3 displays the distributions of Diff=H(T|aa)−H(F|aa) values as box plots for the five groups with the largest number of species.

If the base position and the amino acid coded were statistically independent, we would expect maximum entropy values of H(T|aa)=H(F|aa)=2 bits. However, these theoretical maxima were never reached in the species studied, indicating that there is always some degree of dependence between bases and amino acids. In fact, the maximum observed H(T|aa) was approximately 1.5473, found in the basidiomycete fungus *Fibroporia radiculosa*. Conversely, the maximum H(F|aa) was approximately 0.2420, corresponding to the Marburg marburgvirus, which causes a form of viral hemorrhagic fever in humans and other primates. It is noteworthy that max(H(T|aa)) is more than six times larger than max(H(F|aa)), which implies that the conditional entropy of the third base, given the amino acid, can exhibit significantly more variation than the conditional entropy of the first base, given the amino acid. The estimated Pearson’s correlation coefficient between H(T|aa) and H(F|aa) was $\hat{r}\approx 0.7492$, with an *R*^2^ value of ${\hat{r}}^{2}\approx 0.5946$. This suggests that approximately 60% of the variance in H(T|aa) can be explained by the variance in H(F|aa).

As stated previously, Diff=H(T|aa)−H(F|aa) was consistently positive across all species. This difference ranged from a minimum of approximately 0.6882, observed in the methanogenic Archaea *Methanobrevibacter wolinii* [26], up to a maximum of approximately 1.3646, found in another Archaea, *Haloquadratum walsbyi*, a halophilic microorganism characterized by flat, square–shaped cells.

The median of Diff=H(T|aa)−H(F|aa) across all species was approximately 1.2113. The mean was approximately 1.1858, with a 99% Confidence Interval ranging from 1.1799 to 1.1918. These values are well above the zero threshold, which would have falsified the hypothesis H(T|aa)>H(F|aa).

In Fig 3, we observe the distributions of Diff=H(T|aa)−H(F|aa) across the five groups with the most numerous samples. The distributions generally range from a minimum of 0.8 to a maximum greater than 1.35, excluding outlier values. For all five groups, the interquartile range is well above 1 bit, and the medians and means of the differences exceed 1.1 bits. This collectively provides strong evidence favoring the hypothesis H(T|aa)>H(F|aa), at least within the sampled species.

Among the five distributions in Fig 3, the Archaea distribution exhibits the smallest mean and median, yet also the largest spread, as indicated by both its interquartile range and whisker length. This extensive variation in the Diff parameter supports the notion that Archaea represents the most molecularly diverse of the three domains of life. Indeed, the archaeal tree has expanded dramatically with new molecular data [27] and continues to grow thanks to metagenomic approaches like that in [28], where the authors reveal a vast and previously overlooked diversity within Archaea. In contrast, the EnteroBac distribution in Fig 3 is highly symmetric (its mean and median coincide), and its interquartile range is almost entirely contained within the second quartile of the Archaea distribution, confirming that the sampled bacteria are much less diverse in Diff values than Archaea.

Conversely, the three eukaryotic groups in Fig 3 |Animals, Fungi, and Plants| demonstrate higher mean and median Diff values compared to those estimated for microbes (Archaea and EnteroBac). This suggests that the coding role of the third base is less constrained than that of the first base in Eukarya compared to Bacteria or Archaea. In turn, this could be attributed to factors such as the higher complexity (presence of a nucleus and membrane–bound organelles), larger cell size, multicellularity, and longer generation times characteristic of eukaryotes. These differences collectively influence evolutionary pathways, which over very long timescales, have produced distinct coding strategies among species groups (see also S8 Table in S2 Text).

We assert that understanding these differences in coding strategies and relative efficiencies in the use of the genetic code is the essential first step towards a better comprehension of the molecular grammar of life.

### Standardized mutual information between components of codons

The quantity I(X;Y)=H(X)−H(X|Y)=H(Y)−H(Y|X), known as “Mutual Information” (hereafter abbreviated as MI), quantifies the informational dependence between two sets of events, *X* and *Y*. In this regard, it serves a purpose analogous to Pearson’s correlation coefficient, as both measure the strength of a relationship between variables, although MI is capable of capturing both linear and nonlinear dependencies (For further details and interpretation, please refer to the file “IR2025ShannonInfo.pdf” within the “Shannon.codon” [17] package).

Although Shannon’s foundational paper [5] did not use “Mutual Information” as a specific term, this quantity was derived and discussed therein as “equivocation” or “reduction of uncertainty”. MI has since been widely used, directly or indirectly, for various applications, such as broadly quantifying information in DNA binding sites [29], clustering gene expression data [30], or defining gene specificity [9].

**Fig 4:**
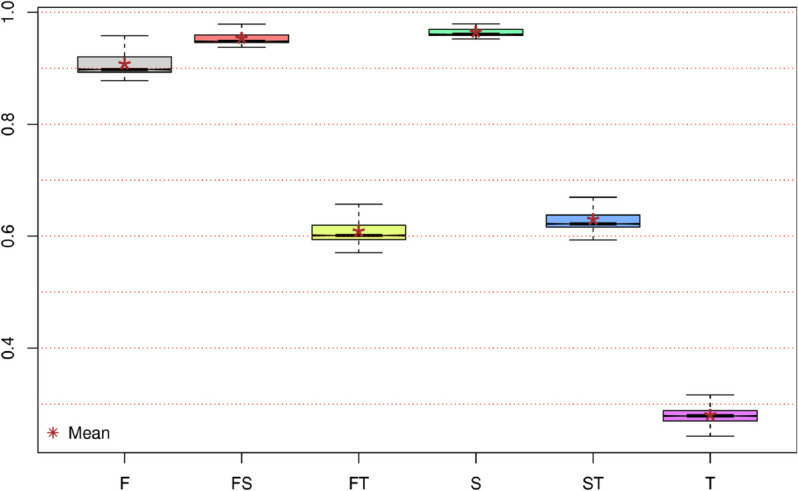
Distributions as box plots of standardized mutual information measures between aa and other codon components in the 1,434 species studied. Distributions for sI(aa;F), sI(aa;FS), sI(aa;FT), sI(aa;S), sI(aa;ST), and sI(aa;T) are displayed; the second component of the *sI* is shown as a label on the *X*-axis.

Here, we employ a standardized version of MI, sI(X,Y), which can take values ranging from zero (when the two terms are statistically independent) to one (when full dependence exists; see S4 Sect in S2 Text). We focus on the overall dependence estimated between amino acids (*aa*) and inner codon components. Fig 4 illustrates the distributions of these measures across the entire set of 1,434 species studied.

In Fig 4, the first notable observation is the high compactness of the six distributions of standardized MI measures across all sampled species. The standard deviation (*S*) for these distributions varies from a minimum of approximately 0.006 for the dependency between amino acids and the second base, sI(aa;S), up to a maximum of approximately 0.024 for the dependency between amino acids and the third base, sI(aa;T). These small spreads inherently indicate that the informational dependencies between amino acids and internal codon parts are well-defined and consistent across the sampled species.

In contrast, the central tendencies of these distributions vary widely, ranging from a minimum average of 0.2800 for sI(aa;T) (represented by the rightmost box plot in Fig 4) to a maximum of 0.9637 for sI(aa;S). The distributions presented in Fig 4 are congruent with the expected informational structure of the genetic code. While sI(aa;F), sI(aa;FS), and sI(aa;S) show distributions close to the maximum value of 1, the distribution for sI(aa;T) exhibits low dependency values. This latter fact is consistent with the secondary role played by the third base in amino acid determination.

The highest value among all measures presented in Fig 4 is approximately 0.9791, corresponding to an sI(aa;S) value –the standardized MI between amino acids and the second base (the fourth box plot from the left in Fig 4). This exceptionally high dependency value is observed in *Methanopyrus kandleri*, a hyperthermophilic Archaea discovered on the wall of a black smoker in the Gulf of California at a depth of 2,000 m, thriving at temperatures between 84–110 °C. This organism also shows high dependency values for sI(aa;FS)≈0.9544 and sI(aa;F)≈0.9089, suggesting that the extreme conditions in which this species evolved have driven such values to the highest observed dependency among the sampled species. Conversely, for this same species, the low value of sI(aa;T)≈0.2511 implies a lack of strong selective pressure on the third codon base, allowing it more freedom to vary.

**Fig 5:**
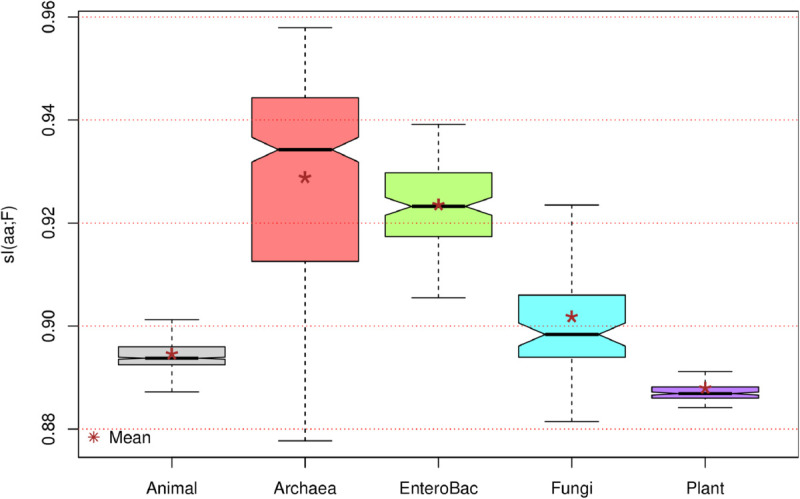
Distributions as box plots of the standardized MI sI(aa;F) per species group.

Now, let’s focus on the distributions of the standardized MI between amino acids and the first base of codons, sI(aa;F), for the five most sampled species groups, as presented in Fig 5.

In Fig 5, you can see that the distribution of sI(aa;F) for Archaea has the largest spread, while the Plant distribution is the most compact. Across all five species groups, the sI(aa;F) distributions are almost entirely contained between 0.88 and 0.96, confirming the strong dependency between the first base and the encoded amino acid.

The order of central tendency (means and medians) for the distributions in Fig 5 is Archaea > EnteroBac > Fungi > Animal > Plant. This suggests that the selective pressures and, consequently, the evolutionary paths of species in these groups have been quite distinct. While the interquartile range of the EnteroBac distribution is fully contained within the second quartile of the Archaea distribution, the distributions for the eukaryotic groups |Fungi, Animal, and Plant| are well below the interquartile range for Archaea. This indicates that factors driving eukaryotic evolution are, in some ways, less stringent than for Archaea or EnteroBac, possibly due to multicellularity and longer generation times in eukaryotes.

S8 and S9 Figs in S2 Text present the distributions of sI(aa;S) and sI(aa;FS) for the five most numerous species groups, respectively. These figures resemble Fig 5 in both their relative spread and the order of central tendency measures among the groups. However, as observed in the distributions in Fig 4, sI(aa;S) and sI(aa;FS) are generally larger than sI(aa;F). In fact, we found that in all 1,434 species, sI(aa;S)>sI(aa;F), which is intriguing because it means the dependence of the encoded amino acid is larger for the second codon base than for the first. At first glance, this seems counter–intuitive given that the second base, in principle, has a less relevant role than the first for determining the amino acid (see S10 Fig in S2 Text).

**Fig 6:**
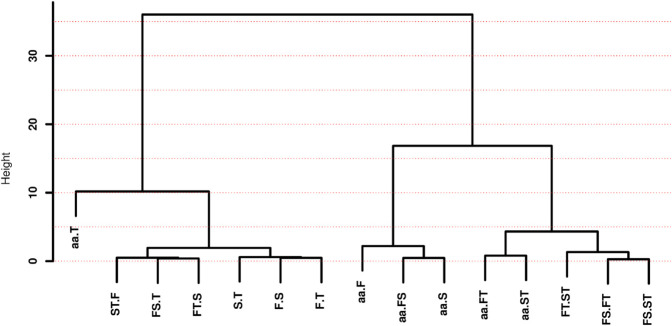
Dendrogram for the 15 *sI* measures in the 1,434 species. Labels are formed by the two terms in the *sI* measures; for example, sI(aa;T) has the label **aa.T**.

The relationships among the 15 *sI* measures of dependency between different codon parts, as measured in the 1,434 species, are complex. To gain a better understanding of these relationships, we constructed a dendrogram, which is presented in Fig 6.

In Fig 6, two large clusters are visible, separated at a height greater than 35. The cluster on the left-hand side of the dendrogram groups *sI* measures that exhibit consistently low values across almost all species. Indeed, the medians of the measures in this cluster |sI(aa;T), sI(ST;F), sI(FS;T), sI(FT;S), sI(S;T), sI(F;S), and sI(F;T)| are all smaller than 0.3. Notably, among these seven measures, only one, sI(aa;T), includes the amino acid term (*aa*), clearly reaffirming the secondary role played by the third base in amino acid determination.

Conversely, the large cluster on the right-hand side of the dendrogram comprises eight *sI* measures that demonstrate a high degree of dependence between their terms, with a median of at least 0.5223 across all species. Within this cluster, sI(FS;FT) is grouped with sI(FS;ST) at a height very close to zero, indicating that these measures are highly similar across all species; their Pearson correlation coefficient is, in fact, $\hat{r}\approx 0.9554$. This right-hand cluster includes all *sI* measures containing the *aa* term (except for the aforementioned sI(aa;T)). The height at which these are grouped in the dendrogram also serves as an inverse measure of correlation: a higher dendrogram height implies lower correlation between the corresponding *sI* values.

The overall panorama of dependency relations between codon parts presented in Fig 6 allows for a better understanding of the complex grammars embedded in the genetic code by segregating and ordering such dependencies (see S4 Sect in S2 Text).

### Comparing species locations in the RCF × H dimensions

For each species, we obtained a vector of 54 *H* entropy measures (S10 Table in S2 Text) from the 64 Relative Codon Frequencies (RCF). We have shown that these measures help in understanding the implicit rules of the genetic code. In S5 Sect within S2 Text, we investigate the complexity of the *H* landscape, determining that neither the RCF nor the *H* spaces provide a perfect segregation of species by group. That is, dendrograms constructed from Euclidean distances in either space do not perfectly segregate species groups. This is likely due to the evolutionary process not proceeding linearly in the modification of RCF or *H* measures over very long timescales, even though the consistency of clusters in the *H* space is greater than 98% for pairs of species with small divergence times (S12 Table and S12 Fig in S2 Text). Furthermore, horizontal gene transfer between Bacteria and Archaea cannot be ruled out [31].

**Fig 7:**
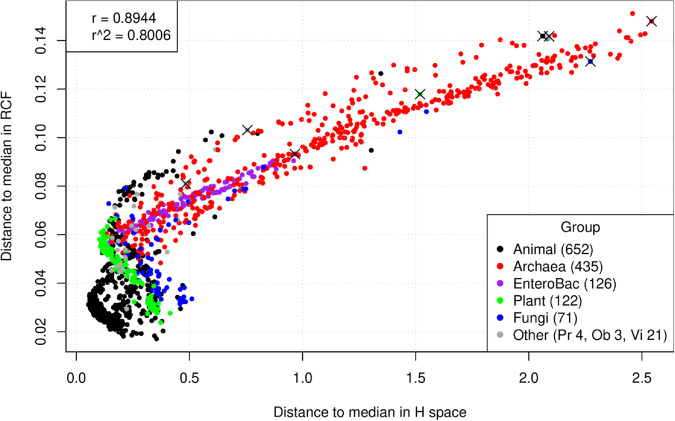
Dot plot of distances to medians for each of the 1,434 species in the *H* (X-axis) × RCF (Y-axis) spaces. Outliers in *X* marked with ×; see Table 4.

A useful way to visualize the spread and position of each species is to consider its distance to the median in each of the two spaces (RCFs and *H* values). Fig 7 presents a dot plot showing the distances of each species to the medians of the RCF × H spaces, with points colored by species group.

In Fig 7, we observe that Archaea (represented by red circles) is the group with the largest spread in both coordinates. This aligns with the larger spread for Archaea noted in the distributions shown in Figs 3 and 5, confirming that the Archaea group exhibits the greatest variability among the eight groups studied. The linear correlation for the distances to the medians in both spaces is high ($\hat{r}\approx 0.8944$); however, this value is primarily driven by the distances of the Archaea species to the medians.

**Fig 8:**
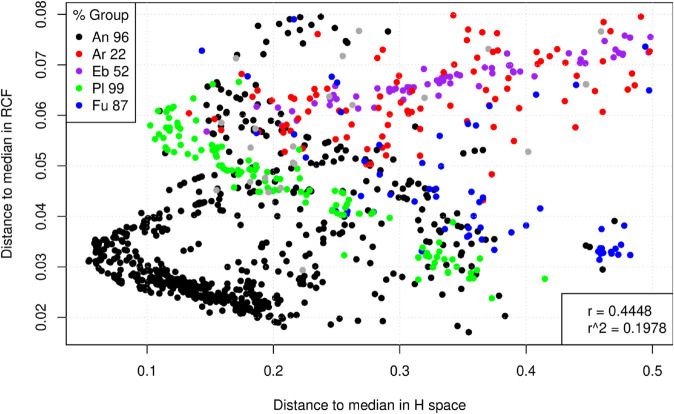
Zoom view of the lower-left corner of Fig 7.

To examine the panorama of smaller distances to the medians in both spaces more closely, Fig 8 provides a zoomed-in view of the lower-left corner of Fig 7, including only species within the box X≤0.5,Y≤0.08. Fig 8 contains large percentages of eukaryotic species: 96% of Animals, 99% of Plants, and 87% of Fungi. In contrast, it includes smaller percentages of prokaryotes: only 22% of Archaea and 52% of EnteroBac (as indicated in the upper-left legend). When considering only species close to the medians in both axes, the estimated $\hat{r}$ decreases to approximately 0.4448, almost half of the correlation estimated with the entire dataset in Fig 7. In Fig 8, many of the animal species (black dots) form a “<” pattern in the lower-left portion of the graph. This pattern implies a strong, divergent, and non-linear behavior in the RCF × H space for this group of species. Also in Fig 8, the behavior of plants (green circles) is approximately inverse, tending to exhibit high distances to the median in the *H* space that are negatively correlated with low distances to the median in the RCF space.

Overall, Figs 7 and 8 demonstrate that while the relationship between distances to the median in the RCF × H plane is approximately linear for Archaea, this relationship is more complex for the other seven species groups. This implies that studying entropies contributes to revealing aspects of genetic code usage that cannot be directly inferred from codon frequencies alone.

Table 4 presents the eight species with the largest distances to the medians, one representative from each of the eight groups. Those outlier species are marked with an × symbol in Fig 7.

**Table 4:** Species with larger distances to medians.

Label	*H* dev.	Scientific name	Comment
An587	2.06	*Strongyloides ratti*	Nematode (parasite) found in wild rats.
Ar41	2.54	*Halarchaeum rubridurum*	Halophilic archaea in the family Halobacteriaceae.
Eb37	0.97	*Cronobacter universalis* NCTC 9529	Gram-negative, Enterobacteriaceae.
Pl122	1.52	*Chlamydomonas reinhardtii*	Single-cell green alga.
Fu44	2.27	*Rhodotorula graminis* WP1	Fungus of class Microbotryomycetes.
Vi4	0.48	Human immunodeficiency virus 1	HIV-1 virus causes human HIV.
Pr4	2.09	*Entamoeba nuttalli* P19	Amoeba prevalent in macacos
Ob2	0.76	*Endomicrobium proavitum*	Ultramicrobacterium; fixes nitrogen.

First two letters in **Label** denote the group; An – Animal, Ar – Archaea, Eb – EnteroBac, Pl – Plant, Fu – Fungus, Vi – Virus, Pr – Protist and Ob – OtherBac. See Table 1 and S5 Sect within S2 Text for details. Column “***H* dev.**” is the deviation from the median in the *H* space (*X* coordinate in Figs 7 and 8).

Species listed in Table 4 are atypical within their respective groups, suggesting they have followed unusual evolutionary paths, strongly deviating from the medians in both RCF and *H* spaces (these species are marked with an “×” in Fig 7). The peculiar profile in the *H* space of these species could offer clues about the selective pressures potentially associated with unusual lifestyles or ecological niches.

The first row in Table 4, with label An587, corresponds to the animal species *Strongyloides ratti*. This species is the fourth most extreme in the *H* space, with a distance to the median of 2.06 units. Phylogenetic evidence suggests that this nematode’s transition to rat parasitism represents an independent evolutionary pathway within the genus [32]. Notably, parasitic females of this species reproduce by mitotic parthenogenesis, a very rare form of reproduction among animals.

The second row in Table 4, labeled Ar41, represents the most extreme organism among all sampled species, with a distance to the median of 2.54. This species is marked with an “×” symbol in the upper-right corner in Fig 7. This Archaea, *Halarchaeum rubridurum*, belongs to the halophilic genus *Halarchaeum*. Genomic analysis has revealed that members of this genus prefer amino acids over carbohydrates as their primary energy source in high-salinity environments, possessing crucial genes associated with the corresponding metabolic pathway [33].

The row labeled Eb37 in Table 4 corresponds to *Cronobacter universalis*, a species known to produce a very particular *O*-polysaccharide and cause severe illness in highly vulnerable neonates, infants, and the elderly [34]. This Gram-negative Enterobacteriaceae is considered an opportunistic human pathogen that poses a risk to vulnerable populations [35]. This organism is not particularly extreme in the *H* space, with a distance to the median of 0.97, ranking it sixth among the outliers.

*Chlamydomonas reinhardtii*, labeled Pl122 in Table 4, is a clear outlier among plants (marked by a green point crossed by an “×” symbol in Fig 7), with a distance of 1.52 to the median in the *H* space. This unicellular green alga serves as an important genetic model, possessing a wealth of mutants with lesions in structural, metabolic, and regulatory genes [36]. Our results indicate that this representative of unicellular green algae, *C. reinhardtii*, is significantly different from other sampled plant species and closer to some Archaea in both RCF and *H* spaces.

*Rhodotorula graminis*, labeled Fu44 in Table 4, has a distance of 2.27 to the median in the *H* space, making it the second most extreme organism, surpassed only by the Archaea Ar41. This endophytic, pink-pigmented, and encapsulated yeast strain, belonging to the Basidiomycota phylum, was isolated from the stems of *Populus trichocarpa* [37]. Its unique lifestyle likely induced an evolutionary pathway that segregated it from other fungi. This segregation is partly explained by *Rhodotorula graminis* having one of the highest GC-rich genomes (67%) among all fungal genomes and also presenting the largest asymmetry in C/G content observed among the introns of all fungal genomes[37].

The least extreme outlier presented in Table 4 corresponds to the HIV-1 virus (label Vi4), which has a distance of only 0.48 units to the median (the point with an “×” symbol on the left-hand side in Fig 7). Even though only 21 viruses were studied, this organism’s deviation within its group could be attributed to its diploid nature and the fact that it undergoes approximately two to three recombination events per genome per replication cycle, providing a high source of variability [38].

In Fig 1, we observed that Protists, along with Archaea, are the two groups with the highest separation from all other six groups in the main *H* space. In Table 4, the representative for Protists, labeled Pr4, is *Entamoeba nuttalli*, which has a distance to the median of 2.09, making it the third most extreme organism in the *H* space. This organism is a Protist, specifically a protozoan, and is the genetically closest species to *E. histolytica* |the causative agent of amebiasis in humans| in current phylogenetic analyses of *Entamoeba* species [39].

Finally, the last row in Table 4 presents *Endomicrobium proavitum*, with label Ob2. With a distance to the *H* median of 0.76, this species is not particularly extreme. In [40], the authors characterized the genome of a free-living *Endomicrobium proavitum* strain and compared it with a closely related uncultured endosymbiont strain of termite gut flagellates from the same genus. However, given that only three species from the OtherBac group were analyzed, the results for this particular species cannot be considered highly relevant.

### Context and scope of our informational analysis

It is important to frame our findings in the context of our analytical approach. Our study does not directly analyze the variation in codon distributions across species. Instead, we provide insight into the relationships and dependencies between the informational measures that we derived from those distributions. Our methodology, by focusing on these emergent properties, offers a unique window into the underlying grammar of the genetic code that is complementary to traditional sequence-based phylogenetic analysis. By examining how measures such as sI(F;S) and sI(FS;T) are related, we can infer broader principles of how genetic information is structured and processed. Finally, we acknowledge that GC content is a well-known factor in codon usage bias and, as expected, we found it to be a significant correlate with our codon distributions. While a full exploration of this relationship is outside the primary scope of this work, our methods provide a framework for future studies to investigate the interplay between GC content and the informational dependencies we have identified.

## Conclusion

This study demonstrates how applying measures derived from Shannon’s communication theory can significantly enhance our understanding of the molecular rules and informational structure implicit in the genetic code. A key methodological contribution of this work is the novel segregation of codon functional parts, enabling the estimation of average uncertainties (entropies) for each component.

By employing these information-theoretic tools across 1,434 diverse species, we provided robust evidence that the first codon base plays a central role in amino acid determination, while the third base serves an accessory function. Crucially, we showed that while this degeneracy is evident from the genetic code’s structure, our comprehensive analysis, utilizing conditional entropies (H(T|aa)>H(F|aa)), confirmed this accessory role of the third base across all sampled organisms, representing the most extensive test of this principle to date across the tree of life. Furthermore, our work clarified the distinct informational role of the second codon base.

Our analysis revealed significant heterogeneity in coding strategies across different taxonomic groups. Notably, the unique variability observed in Archaea, contrasting with the more constrained patterns in Eukaryotes and Bacteria, underscores the profound influence of evolutionary pressures and distinct life histories on genetic information processing. The identification of outlier species, exhibiting peculiar informational profiles, highlights specific instances where unusual lifestyles or ecological niches may have driven unique adaptations in codon usage and underlying informational dependencies.

The complex interplay of informational dependencies between various codon parts, as elucidated by our standardized mutual information analysis (Fig 6), offers a novel perspective on the intricate grammar embedded within the genetic code. This segregation and ordering of dependencies provide a richer understanding that extends beyond traditional phylogenetic or codon frequency analyses.

GC-content is a fundamental genomic trait linked to many key genomic features, such as codon and amino acid usage. This trait is well-understood and widely used by scholars in the field to provide a concise molecular summary of genetic code usage for each species. To directly address whether our informational measures are merely a variant of GC-content, we examined the relationship between these parameters through detailed dot plot analyses. Our findings reveal that these relationships are complex and often non-linear, with the entropy measures providing a distinct signal that better segregates species into their taxonomic groups than GC-content alone. We conclude that our entropy measures provide valuable information that cannot be obtained directly or indirectly from GC-content, demonstrating their unique contribution to understanding the molecular grammar of the genetic code (see S6 Sect in S2 Text).

This work not only offers a novel framework for quantifying informational properties of codon usage but also reveals previously unappreciated aspects of how genetic information is encoded and processed across life’s domains. This foundational insight into the “molecular grammar” of life paves the way for future detailed studies investigating how specific selective pressures, environmental factors, and evolutionary pathways have shaped the efficiency and variability of genetic coding strategies.

## Supporting information

S1 Dataset Original data for the 1,434 species in this study. (CSV)

S2 Text Supporting text. (PDF)
