# Supplementary material for: Sampling informational properties of codon usage through the tree of life
Source: PLoS One. 2025 Nov 26;20(11):e0335824. doi: 10.1371/journal.pone.0335824 (PMC12654954; doi:10.1371/journal.pone.0335824)
Supplement: S2 Text — (PDF) [file pone.0335824.s002.pdf]

Supporting Information for
"Sampling informational properties of codon usage through the tree of life"

Important notes

Sections of this document are referred in the main text by the numeration given below in "**Content**", e.g., "**S2**", etc. Also, for tables and figures, e.g., "Table **S1**", "Figure **S1**", etc. To make reading easier, some figures and tables in the main text are repeated here.

Computations were carried out using our R packages "`Shannon.codon`" (Martínez, 2025) and

"`DendroLikeness`" (Martínez, 2024). For details of methods, please see the documentation of those packages.

The binary R file "`SuppResCodonUse.RData`" contains all relevant objects and is available upon request from the corresponding author (see the Appendix section here).

Content

S1. Data and Tools 1
S2. Analyses of main entropy measures 2
S3. Analyzing average uncertainties of the first and third bases 7
S3.1. First hypothesis: $\mathcal{H}_0 : H(T) \geq H(F)$ 7
S3.2. Second hypothesis: $\mathcal{H}_0 : H(T|aa) \geq H(F|aa)$ 9
S4. Mutual information between components of codons 11
S5. The complexity of the H's landscape 17
S5.1. Studying distances within and between species groups. 23
S5.2. The largest distances between species in the H space. 24
S5.3. Species close or far to the median per space (H and codon relative frequencies). 28
S6. Relations between GC-content and main entropy measures 36
References 42
S7. Appendix: R objects containing results. 43

S1. Data and Tools

Raw data for this study consist of all genomic codon frequencies for 1,434 species, downloaded from the "Codon Statistics Database" (Subramanian et al., 2022). All calculations were performed in R (R Core Team, 2013), using the facilities programed in our package "`Shannon.codon`" (Martínez, 2025) and "`DendroLikeness`" (Martínez, 2024).

The aim of the study is to analyze codon frequencies from the perspective of Shannon Communication Theory (Shannon, 1948), and within the philosophical framework proposed by Christoph Adami (Adami, 2016), which conceptualizes information as differences of entropies; see also Adami (2004, 2024).

Our first challenge was to select a set of species to represent the immense diversity of the tree of life which could give an ample but tractable set of results. Table **S1** presents the groups of species included in this work.

The groups presented in Table **S1** represent the main domains of life, say Bacteria, Archaea and Eukaryotes (animals, plants, fungi and protists), plus a set of virus. Even when this sample of 1,434 species is strongly uneven (the number of species per group in column n goes from only 3 for Other Bacteria up to

Date: September 18, 2025.

TABLE **S1**. Groups of species included in this study.

key	Full name	Abbreviation	n	Comment
`An`	Animal	Animal	652	Metazoan, including among others: bats (3), birds (9), bovine (12), primates (25), rodents (21), carnivora (21), fish (11), etc.
`Ar`	Archaea	Archaea	435	A domain of single-celled microorganisms, distinct from bacteria and eukaryotes.
`Eb`	Enterobacteriaceae	EnteroBac	126	Gram-negative bacteria.
`Pl`	Plant	Plant	122	Mainly land plants including the 3 most important cereals and 8 significant Solanaceae.
`Fu`	Fungus	Fungi	71	68 *Basidiomycota* and 3 species of *Saccharomyces.*
`Vi`	Virus	Virus	21	Incudes mainly human viruses and some plant viruses.
`Pr`	Protist	Protist	4	Amoebas: *Entamoeba dispar, E. histolytica, E. invadens* and *E. nuttalli.*
`Ob`	Other Bacteria	OtherBac	3	*Elusimicrobium minutum, Endomicrobium proavitum* and *Thermococcus barophilus.*
**Total of species included**			1,434	

652 for Animal), it gives interesting findings about the informational structure of codon usage through the tree of life.

The column "Abbreviation" in Table **S1** shrinks the "Full name" of the group to be presented in the analyses and tables, why the "`key`" column is used to computationally distinguish each group within R objects.

The document "`ShannonCodonGuide.pdf`" (available with the "`Shannon.codon`" package) explains in detail how the codon can be segregated into its functional components to estimate various averages of uncertainties (entropies). Those measurements include 8 quantities depending of a single term, 6 join entropies, 25 conditional entropies and 15 mutual information terms; a total of 54 non-redundant and non-null entropies for each one of the 1,434 species (see Table **S10** for the full list of H's). This gives a total of $54 \times 1,434 = 77,436$ numerical results which need to be summarized and interpreted.

The approach for the analyses was to divide the results on different sets by group of species and, when possible, propose reasonable hypotheses based in the nature of the genetic code structure, and then examine those assumptions under the light of the estimated results.

S2. ANALYSES OF MAIN ENTROPY MEASURES

The relative frequencies of the 64 codons in each species depend, firstly, on the amino acid composition of all proteins encoded in the corresponding genome (the proteome), and secondly, on the preference of codon usage in the cases of synonymous codons, i.e., in the codon bias per amino acid within the species. These two sources of variation can be separated by considering both, the amino acid (aa) coded, as well as the functional segments of the codons, for which we have 3 duplets, the one formed by the First and Second bases, FS, the one formed by the First and Third bases, FT, and the one formed by the Second and Third bases, ST. Additionally we must take into account the positions of the individual bases, the First, F, Second, S, and Third, T, base within each codon.

This partition allow us to consider different sources of variation, which imply sets of relative frequencies, $\mathbf{p}$, with different lengths. Table **S2** presents the sources of variation for the codon partitions.

For each one of the cases presented in Table **S2** the vector $\mathbf{p}$ contains the relative frequencies or probabilities of each one of the observed members, for example, for the case of codons we have

$$\mathbf{p} = (p_1, p_2, \cdots, p_{64})$$

TABLE **S2**. Sources of variation, instances and associated H's for codon partition.

Source	Instances	$k = \mathbf{p}$ length	H's	max(H)
Codon	codon	64	H(codons)	$\log_2(64) = 6$
Amino acid	aa	21	$H(aa)$	$\log_2(21) \approx 4.3923$
Duplets	FS, FT, ST	16	$H(FS)$, $H(FT)$, $H(ST)$	$\log_2(16) = 4$
Bases	F, S, T	4	$H(F)$, $H(S)$, $H(T)$	$\log_2(4) = 2$

where p_i is the relative frequency observed for the i-th codon. In that case the value of H(codons) is obtained by applying Shannon's entropy formula, say H(codons) will be equal to

$$-\sum_{i=1}^{i=64} p_i \log_2(p_i)$$

and the formula for each one of the H in Table **S2** is always the same, except that it is applied to the corresponding relative frequencies of the corresponding instance.

Table **S2** also presents the column "max(H)" with the maximum value that the corresponding H's can reach. Those maxima depend only on the length of the vector of probabilities, column "$k = \mathbf{p}$ length", and in general we have that the maximum value of H, $\log_2(k)$, is reached when all probabilities in $\mathbf{p}$ are identical to $1/k$. In summary, for each species we have 8 main values of H,

$H(\text{codon}), H(aa), H(FS), H(FT), H(ST), H(F), H(S)$ and $H(T)$.

To have a comparative idea of the values of the H's in each group of species, Table **S3** presents the values of the medians of each quantity and their percentage with reference to the maximum possible for each one of the species groups.

TABLE **S3**. Median values of main entropy measurements per group of species. First row per group gives the raw value, while second row gives percentage with reference to the maximum possible in each case. The maximum of each column is presented in **bold**, while the minimum of each column is shown in *italics.*

			Duplets			Bases		
Entropy ⇒	H(codon)	$H(aa)$	$H(FS)$	$H(FT)$	$H(ST)$	$H(F)$	$H(S)$	$H(T)$
Animal	5.78	**4.20**	**3.91**	3.92	3.90	1.97	1.97	1.98
($n = 652$)	96.34	**95.54**	**97.66**	97.93	97.5	98.32	98.65	98.77
Archaea	5.44	*4.08*	3.78	3.61	3.67	*1.86*	**1.98**	1.76
($n = 435$)	90.6	*92.84*	94.45	90.26	91.83	*93.00*	**98.95**	87.99
EnteroBac	5.62	4.16	3.88	3.82	3.83	1.94	1.97	1.93
($n = 126$)	93.59	94.81	97.05	95.58	95.7	96.86	98.75	96.5
Plant	**5.79**	4.19	3.89	**3.92**	**3.91**	**1.97**	1.97	1.97
($n = 122$)	**96.52**	95.48	97.25	**98.12**	**97.76**	**98.37**	98.37	98.54
Fungi	5.78	4.16	3.9	3.9	3.9	1.96	1.98	1.96
($n = 71$)	96.38	94.74	97.6	97.38	97.61	97.95	98.89	97.89
Virus	5.74	4.2	3.89	3.89	3.9	1.94	1.97	**1.98**
($n = 21$)	95.7	95.52	97.14	97.22	97.5	97.19	98.25	**98.78**
Protist	*5.16*	4.16	*3.73*	*3.46*	*3.41*	1.92	*1.88*	*1.56*
($n = 4$)	*85.98*	94.67	*93.29*	*86.50*	*85.32*	95.87	*94.06*	*78.16*
OtherBac	5.57	4.12	3.77	3.83	3.8	1.9	1.93	1.96
($n = 3$)	92.79	93.72	94.32	95.84	94.89	95.23	96.7	98.03
ALL	5.74	4.19	3.89	3.89	3.88	1.96	1.97	1.96
($n = 1,434$)	95.68	95.30	97.18	97.16	97.06	98.10	98.71	98.01

In Table **S3** we present medians instead of means (averages) because the former are less affected by outliers. To interpret the values presented in Table **S3** and their relative percent maxima (in the second row within each group), we must take into account that the entropy, H, quantifies the average uncertainty in the corresponding source, and in principle a value of H close to its maximum possible, i.e., close to 100%, implies an optimum coding from the perspective of Shannon's communication theory.

In Table **S3** all but one of the 72 values of relative percent maxima are above 80%, the exception being the case of $H(T)$ which has a value of only 78.16 for Protist (amoebas); thus in general we observe that genetic coding in the analyzed species is above 80% in efficiency, and the average of all the 72 medians presented is 95.53%.

Focusing our attention in the percent maxima per column (highlighted in **bold** in Table **S3**) we see that Plant is the group with more maxima per column, having larger values of $H(\text{codon})$, $H(FT)$, $H(ST)$ and $H(F)$. This group is followed in that sense by Animal, which have maxima for $H(aa)$ and $H(FS)$, while Archaea and Virus have maxima for $H(S)$ and $H(T)$, respectively.

On the other hand, looking at the minima per column (in *italics* in Table **S3**), we see that Protist (which include only amoebas) have the minimum for codons, $H(\text{codon})$, the three duplets, $H(FS)$, $H(FT)$ and $H(ST)$ as well as for the Second and Third bases, $H(S)$, $H(T)$, i.e., for 5 of the 8 measurements presented. From this fact we can immediately say that informational properties for amoebas are far apart from the other groups. The minimum percent value for $H(aa)$ and $H(F)$, 92.84 and 93.00%, respectively, are found in Archaea, a group that we will confirm later have also very peculiar informational properties.

Now we shift our attention to the relations between pairs of H's estimated for the 1,434 species. With this aim Table **S4** presents the matrix of estimated Pearson's correlation coefficients, $\hat{r}$, between pairs of H's, while Figure **S1** shows the dendrogram constructed using the Euclidean distances between the values of one minus the squared correlation coefficient, $1 - \hat{r}^2$, for pairs of H's and obtained from the 1,434 species.

TABLE **S4**. Matrix of estimated Pearson's correlation coefficients, $\hat{r}$, between pairs of H's measured in the 1,434 species.

	$H(aa)$	$H(FS)$	$H(FT)$	$H(ST)$	$H(F)$	$H(S)$	$H(T)$
$H(\text{codon})$	0.90	0.87	0.98	0.98	0.94	0.03	0.96
$H(aa)$		0.81	0.91	0.86	0.96	-0.16	0.87
$H(FS)$			0.82	0.80	0.90	0.41	0.76
$H(FT)$				0.98	0.94	-0.08	0.99
$H(ST)$					0.89	-0.02	0.99
$H(F)$						-0.01	0.88
$H(S)$							-0.11

In Table **S4** we see that the more highly correlated measurements are $H(T)$ with $H(FT)$ and $H(ST)$, in both cases with $\hat{r} \approx 0.99$, followed by $H(FT)$ with $H(\text{codon})$ with $\hat{r} \approx 0.98$ Consequently these four measurements form the more compact group in Figure **S1**, which groups $H(\text{codon})$, $H(FT)$, $H(ST)$ and $H(T)$ at a very low height < 0.1 This cluster is united to the one formed by $H(aa)$ and $H(F)$ also at a height < 0.1 The measurement that is farther apart from the other seven is $H(S)$, which is linked at a height of almost 1 to the clusters already formed.

In Figure **S1** we can see how linearly-alike are the main H measurements from different sources (see Table **S2**). In this sense the distance between $H(aa)$ with $H(T)$ is small, ≈ 0.08, indicating that the uncertainty due to amino acid (aa) is almost equivalent to the one produced by the First base, and this makes biological sense because the First base is the one with a more important role in aa determination.

Now we will see the panorama of species groups in the 8-dimensional space of the main H measures, and compare such perspective with the one obtained by measuring distances in the 64-dimensional space defined by the relative frequency of codons. Figure **S2** presents the dendrogram constructed from distances

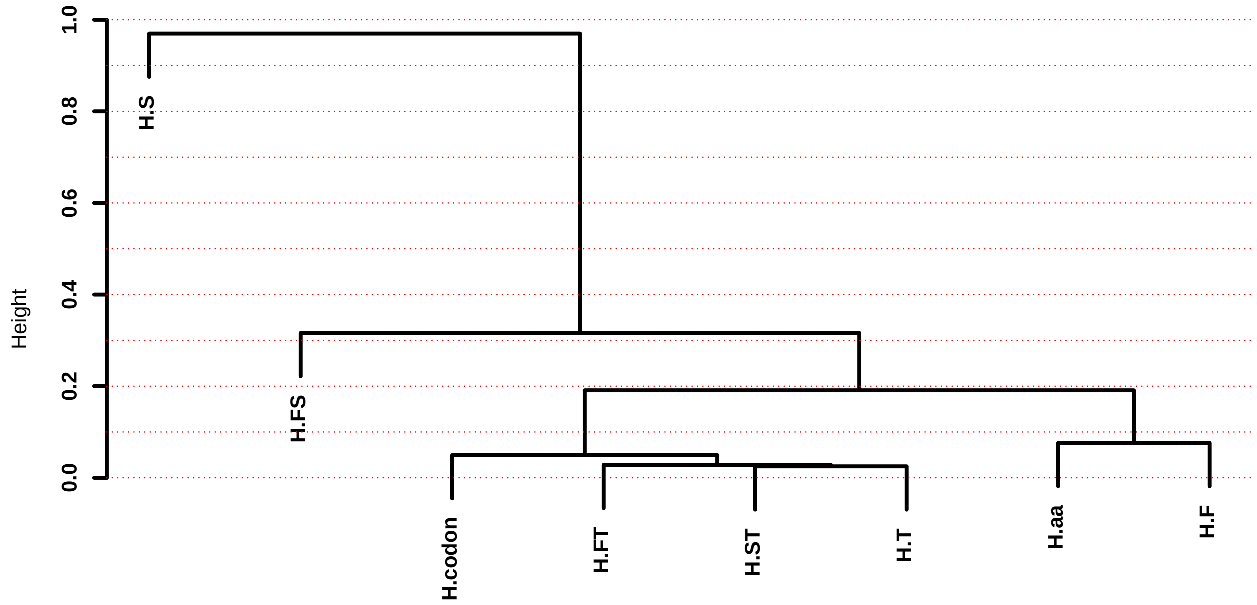

FIGURE **S1**. Dendrogram for the main entropies using as distance $1-\hat{r}^2(a,b)$ where $\hat{r}^2(a,b)$ is the square of Pearson's correlation coefficient and a, b are the different pairs of entropy measures, $H(\text{codon}), H(aa), H(FS), H(FT), H(F), H(S)$ and $H(T)$, shown in the labels as "**H.**" for each term. The values for all the 1,434 species were employed to estimate the values of $1-\hat{r}^2(a,b)$ and the dendrogram was obtained by the "average" (UPGMA) method using the function "`hclust()`".

in H measures, while Figure **S3** presents the one obtained from distances in codon relative frequencies.

Using curly brackets to delimitate clusters, the topology of the dendrogram in Figure **S2** can be represented as

{{{{Fungi, {Virus, {Animal, Plant}}}, {EnteroBac, OtherBac}}, {Archaea, Protist}}}

Of the binary clusters, the one formed by {Animal, Plant} shows the closest group at height ≈ 0.06 while the binary group {Archaea, Protist} is formed at a height ≈ 0.48, i.e., $.48/.06 = 8$ times above than the first one. Intermediate to those two binary groups is the one formed by bacteria, {EnteroBac, OtherBac}, at height ≈ 0.17 Also in Figure **S2**, eukaryotes, excluding Protist but including Virus, are into cluster {Fungi, {Virus, {Animal, Plant}}}, formed at height ≈ 0.14

In contrast with Figure **S2**, the dendrogram presented in Figure **S3** was obtained using as distances the medians between groups of relative codon frequencies, and **not** H values. The topology of the dendrogram in Figure **S3** using curly brackets is

{{OtherBac, Protist}, {Archaea, {EnteroBac, {{Plant, Virus}, {Animal, Fungi}}}}}

In comparing Figure **S3** (codon relative frequencies) with Figure **S2** (H's) we can first notice that their corresponding topologies are completely different; i.e, those dendrograms does not share clusters. This means that grouping by median distances using codon relative frequencies (Figure **S3**) or main H values (Figure **S2**) produces fully different clustering, which in turn means that the corresponding multi-varied spaces do not give alike landscapes at all.

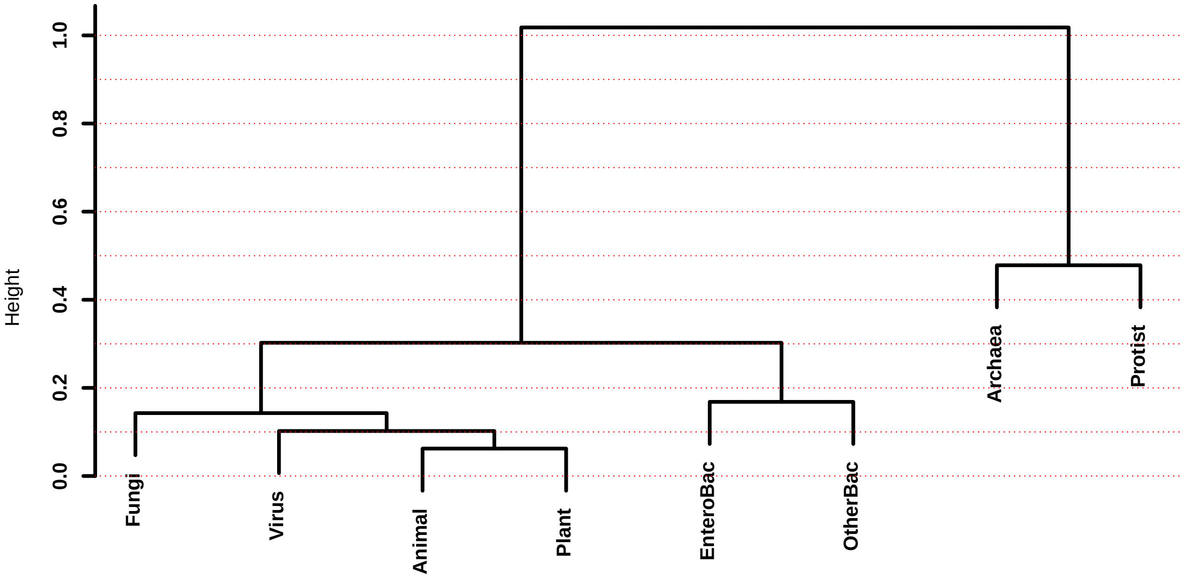

Figure **S2**. Dendrogram for the species groups obtained from the medians of distances between pair of groups in the space of the 8 main entropy measurements, $H(\text{codon}), H(aa), H(FS), H(FT), H(F), H(S)$ and $H(T)$. Dendrogram obtained by the "complete" method using the function "`hclust()`".

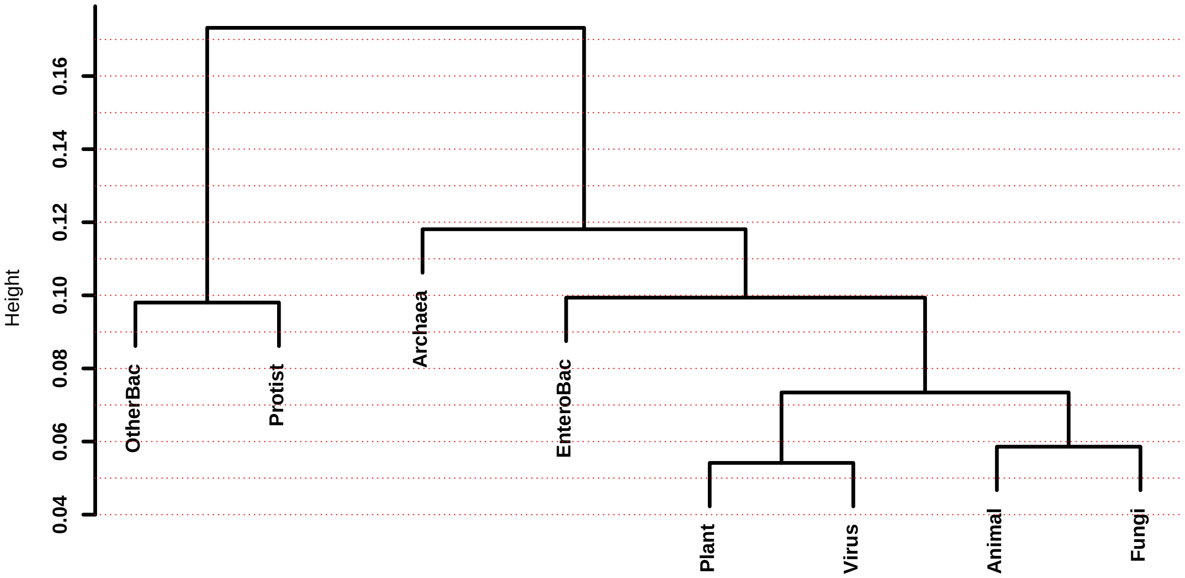

Figure **S3**. Dendrogram for the species groups obtained from the medians of distances between pair of groups in the space of the 64 relative codon frequencies. Dendrogram obtained by the "complete" method using the function "`hclust()`".

To corroborate that distance measurements are different when using codon relative frequencies and H values we standardized all the $(1,434 \times (1,434-1))/2 = 1,027,461$ pair distances in each case, and tested via the `t.test` the absolute differences between between those standardized distances. The mean of the standardized absolute differences between individual distances was ≈ 0.54195, with a 95% confidence interval between 0.5410 and 0.54289, and the p-value of the test was $< 2.2 \times 10^{-16}$, strongly rejecting the null hypothesis that the mean of the absolute differences equaled zero. As an example of the divergence of these distances, when using codon relative frequencies the maximum of the distances was between two

Archean species, while when using H values the maximum distance was between an Archean and an Animal species.

It is important to remark that neither of the two dendrograms (Figures **S2** and **S3**) are intended to reflect taxonomical distances between the groups; that is, the dendrograms cannot be considered as phylogenetic trees.

S3. Analyzing average uncertainties of the first and third bases

$H(F)$ and $H(T)$ quantify the average uncertainties (entropies) due to the First and Third bases, respectively. The First base plays a central role in amino acid (aa) determination, while the role of the Third base is secondary. To evaluate the coding potential of the First and Third base within codons in the genetic code we can observe how many different aa result from the possible instances. Table **S5** presents such data.

Table **S5**. Number of distinct aa coded for instance of the First and Third bases within codons.

Codon position ⇓	Instance (base) A	G	T	C	Mean
First	7	5	5	7	6.00
Third	13	15	14	15	14.25

In Table **S5** the cell in the upper left right hand side corresponds to the column "A" and row "First" has a value of 7. This is because the 16 codons which first base is "A" code for only 7 different aa: "Arg", "Asn", "Ile", "Lys", "Met", "Ser" and "Thr". In contrast, the 16 codons that end with base "A" (second row, "Third", column "A") code for 13 different aa, and so on for the remaining cells. The last column in Table **S5**, named "Mean", presents the average of the corresponding rows, which are 6 for "First" row and 14.25 for the "Third" row. Given that we have 21 different aa (counting the "Stop" signals) Table **S5** exhibits the fact that the Third base is $14.25/6 = 2.375$ times less determinant than the First one for aa codification. This quantifies the fact that a mutation in the Third base within a codon has a smaller probability to cause a change in aa than a mutation in the First base within a codon.

S3.1. First hypothesis: $\mathcal{H}_0 : H(T) \geq H(F)$. Given that H's quantifies average uncertainties, it appears reasonable to postulate as a first hypothesis that the average uncertainty of the Third base is higher than the one produced by the First base, say $\mathcal{H}_0 : H(T) \geq H(F)$.

The rationale for this first hypothesis is that the First base is more restricted to vary than the Third one given the central and secondary role that those codon positions play in aa determination. Table **S6** presents the results obtained in each species group as well as in the total of the 1,434 species.

In Table **S6** we observe that the first hypothesis, $\mathcal{H}_0 : H(T) \geq H(F)$, is true only in approximately 50% of the 1,434 species, more precisely it is observed to be true in 712 species (49.65% presented as 50% in the table due to rounding) and it is false in the remaining 722 species. Thus, at first sight it appears as if the fact $\mathcal{H}_0 : H(T) \geq H(F)$ could be randomly distributed in the set of species studied, being true in approximately half of the species. However, what is more interesting is that the percentages of species for which $\mathcal{H}_0 : H(T) \geq H(F)$ is true are different between species groups, a fact that can be more clearly appreciated in Figure **S4**, which shows the distribution of $H(T) - H(F)$ per species group (only for the 5 groups where more species were sampled).

In Figure **S4** we can first note that the parameter $H(T) - H(F)$, used to judge the truthfulness of the hypothesis $\mathcal{H}_0 : H(T) \geq H(F)$, is very variable for Archaea but much less variable for the groups of superior eukaryotes, Animal and Plant. Concurrently, the only two species groups where $\mathcal{H}_0 : H(T) \geq H(F)$; $H(T) - H(F) > 0$ is true in more than 50% of the species within groups happen for Animals

TABLE **S6**. Raw numbers and percentages (in **bold**) in which $\mathcal{H}_0$ is true or false per group of species (see Table **S1**). Second part presents medians per group.

	Group								
$\mathcal{H}_0$ ⇓	An	Ar	Eb	Pl	Fu	Vi	Pr	Ob	Total
$H(T) \geq H(F)$	415	117	57	72	35	12	1	3	712
% TRUE	**(64)**	**(27)**	**(45)**	**(59)**	**(49)**	**(57)**	**(25)**	**(100)**	**(50)**
$H(T) < H(F)$	237	318	69	50	36	9	3	0	722
% FALSE	**(36)**	**(73)**	**(55)**	**(41)**	**(51)**	**(43)**	**(75)**	**(0)**	**(50)**
Total of Group	652	435	126	122	71	21	4	3	1,434
Medians of quantities per group									
	An	Ar	Eb	Pl	Fu	Vi	Pr	Ob	Total
$H(T)$	1.97541	1.75983	1.93009	1.97073	1.95773	1.97558	1.56313	1.96069	1.96015
$H(F)$	1.96640	1.86009	1.93715	1.96744	1.95900	1.94386	1.91749	1.90464	1.96207
$H(T) - H(F)$	0.00806	-0.10290	-0.00605	0.00341	-0.00425	0.00838	-0.35027	0.05097	-0.00038

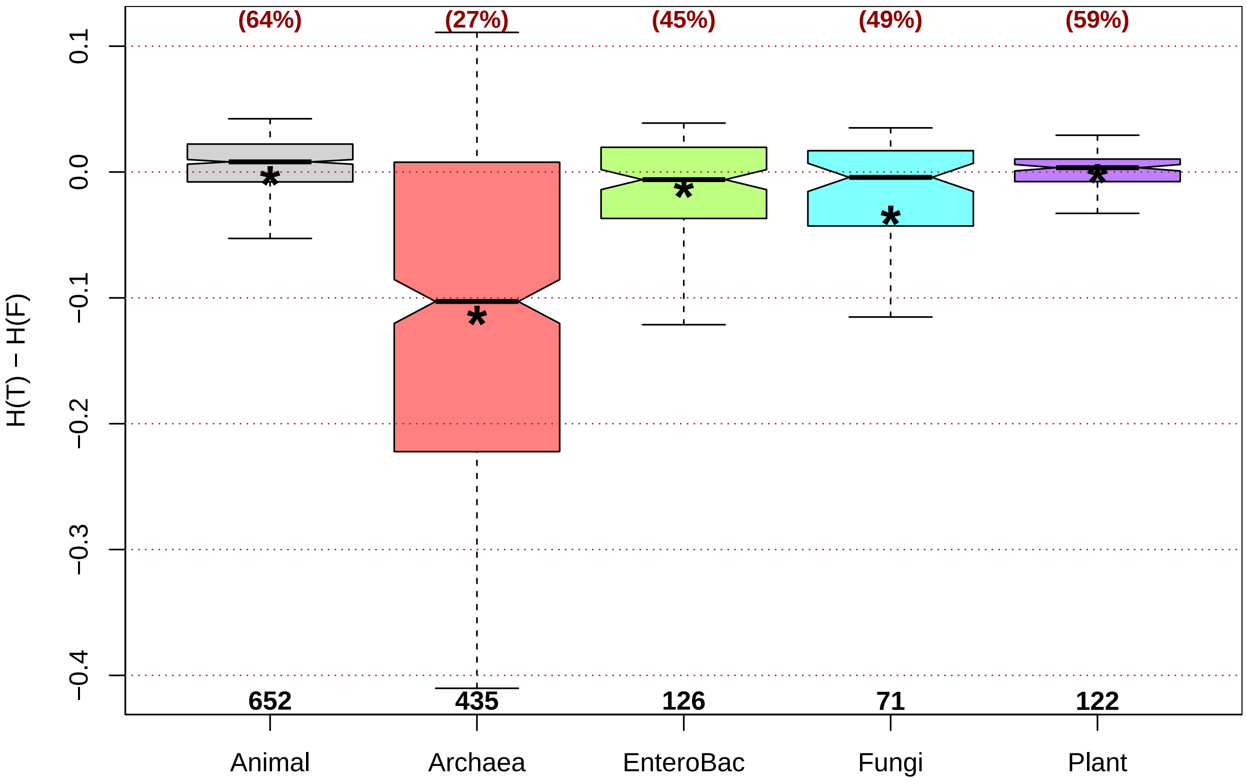

FIGURE **S4**. Distributions as box plots of the values of $H(T) - H(F)$ in the five species groups with more sampled species. Asterisks within each plot point to the position of the mean, numbers in bold at the bottom show the number of species of the corresponding groups while numbers between parenthesis at the top give the percents in which hypothesis $\mathcal{H}_0 : H(T) \geq H(F)$ is true; see also Table **S6**.

(64%) and Plants (59%). This fact suggest that the forces operating during the evolution of Animals and Plants had been more sensitive to the balance between mutation and genetic drift –which could increment $H(T)$, more than in the other groups of species studied.

Selective forces constrain the increase of $H(F)$, given that this First base is preeminent for aa determination, and functional roles of proteins within genomes strongly limit random variation of this First base and, in consequence increases in $H(F)$ are strongly limited during evolution.

S3.2. Second hypothesis: $\mathcal{H}_0 : H(T|aa) \geq H(F|aa)$. We have seen before that the first hypothesis, $\mathcal{H}_0 : H(T) \geq H(F)$; $H(T) - H(F) > 0$ is true in only approximately 50% of the species within groups. Now we will consider the average uncertainties of the Third and First bases but discounting the average entropy due to the amino acids (aa), say, the entropy of the Third base given the one of the amino acids,

$$H(T|aa) = H(T, aa) - H(aa)$$

where $H(T|aa)$ is the conditional entropy observed in the Third position given that the aa are known, $H(T, aa)$ is the joint entropy produced by considering both, the First base as well as aa and $H(aa)$ is the entropy due to amino acids. Similarly we can consider the entropy of the First base given the one of the amino acids,

$$H(F|aa) = H(F, aa) - H(aa)$$

We will consider the second hypothesis of interest, say: $\mathcal{H}_0 : H(T|aa) \geq H(F|aa)$, which can be tested by the difference $H(T|aa) - H(F|aa)$, which when $\mathcal{H}_0$ is true in a given species must be a positive quantity. This second hypothesis makes biological sense because, given the characteristics of the genetic code, $H(T|aa)$ could have a large value because the Third base plays only a secondary role in amino acid determination, while in contrast $H(F|aa)$ must be smaller than $H(T|aa)$ given than the First base plays a more important role in aa determination (see Table **S5**).

Interestingly in all the 1,434 species studied without exception, $\mathcal{H}_0 : H(T|aa) \geq H(F|aa)$ was observed to be true. In fact, the difference $H(T|aa) - H(F|aa)$ presented a positive minimum of ≈ 0.69, with a median of ≈ 1.21, a mean of ≈ 1.19 and a standard deviation, S, of ≈ 0.09 (statistics are given in bits).

Table **S7** presents the main statistics for the groups of species.

In Table **S7** we can corroborate that for all groups of species $\mathcal{H}_0 : H(T|aa) \geq H(F|aa)$ was true, because the minimum of the differences $H(T|aa) - H(F|aa)$ (in column "Diff." in section "**Minima**") was always positive. Furthermore, the smallest of the minima values was ≈ 0.69 for Archaea (column "Ar") while the maximum of those minima, ≈ 1.15, was for Virus (column "Vi").

In summary, the strong evidence presented in Table **S7** suggests that all along the tree of life it is a fact that $H(T|aa) \geq H(F|aa)$, and this in turn implies that the genetic code is optimized for amino acid coding.

Figure **S5** presents the box plots for the differences, $H(T|aa) - H(F|aa)$, for the 5 groups with more species sampled.

We can consider that the difference $H(T|aa) - H(F|aa)$ in some sense quantifies the degree of optimization of the genetic code within the species studied; larger values of $H(T|aa) - H(F|aa)$ imply a better adequacy to the final goal of DNA coding for amino acids.

In Figure **S5** we can observe that, even when all differences $H(T|aa) - H(F|aa)$ are positive and far away from zero, there is considerable variation of this quantity between groups; the differences have a larger spread in Archaea but are highly compact in Animal and EnteroBac (see also section "Standard Deviation" in Table **S7**). Also interesting is the fact that Fungi present the highest mean and median of the differences, implying a stronger optimization of the genetic code in this group of organisms.

Table **S8** presents the species with extreme values of the difference, Diff $= H(T|aa) - H(F|aa)$ for each main group of species. As reference, our own species (*Homo sapiens*) presents values $H(F|aa) \approx 0.21$ $H(T|aa) \approx 1.44$ with a difference $H(T|aa) - H(F|aa) \approx 1.22$, which places humans above the mean and median for the Animal group (Table **S7** and Figure **S5**).

TABLE **S7**. Statistics for $H(F|aa)$, $H(T|aa)$ and its difference (column "Diff", equal to $H(T|aa)-H(F|aa)$) per group of species and also in all the 1,434 species (column "Total").

Group ⇒	An	Ar	Eb	Pl	Fu	Vi	Pr	Ob	Total
n	652	435	126	122	71	21	4	3	1,434
					**Minima**				
Group ⇒	An	Ar	Eb	Pl	Fu	Vi	Pr	Ob	Total
$H(F\|aa)$	0.14484	0.07573	0.11687	0.12905	0.17175	0.14239	0.17310	0.16250	0.07573
$H(T\|aa)$	0.91358	0.84815	1.23907	0.99449	1.31914	1.29318	0.90886	1.28495	0.84815
Diff.	0.73417	0.68821	1.11470	0.86544	1.14322	1.15079	0.73267	1.10955	0.68821
					**Means**				
Group ⇒	An	Ar	Eb	Pl	Fu	Vi	Pr	Ob	Total
$H(F\|aa)$	0.20743	0.13375	0.14835	0.22035	0.19230	0.19879	0.17652	0.17715	0.17997
$H(T\|aa)$	1.41763	1.25766	1.34632	1.44341	1.46113	1.40612	1.02502	1.35736	1.36580
Diff.	1.21019	1.12391	1.19796	1.22306	1.26884	1.20733	0.84850	1.18021	1.18583
					**Medians**				
Group ⇒	An	Ar	Eb	Pl	Fu	Vi	Pr	Ob	Total
$H(F\|aa)$	0.20895	0.12317	0.14878	0.22255	0.19987	0.19594	0.17495	0.17592	0.20073
$H(T\|aa)$	1.42686	1.26804	1.34500	1.44094	1.49709	1.42356	0.91251	1.33483	1.40750
Diff.	1.21733	1.13779	1.19760	1.21891	1.29623	1.21665	0.73911	1.15104	1.21127
					**Standard Deviations (S)**				
Group ⇒	An	Ar	Eb	Pl	Fu	Vi	Pr	Ob	Total
$H(F\|aa)$	0.00927	0.03485	0.01513	0.00908	0.02273	0.02041	0.00463	0.00611	0.04118
$H(T\|aa)$	0.04531	0.13306	0.04544	0.03492	0.09620	0.05126	0.22747	0.05320	0.11531
Diff.	0.04224	0.11434	0.03195	0.03509	0.08273	0.04780	0.22312	0.05744	0.08757

TABLE **S8**. Species with extreme values of the difference, Diff $= H(T|aa) - H(F|aa)$ for each main group of species. First row of Diff. within group presents the minimum and the second row presents the maximum.

Group	Diff.	Label	Species	Comment
Animal	0.73417	An587	*Strongyloides ratti*	Parasitic threadworm
	1.32422	An633	*Varroa destructor*	External parasitic mite
Archaea	0.68821	Ar233	*Methanobrevibacter wolinii* SH	Coccobacillus (from sheep faeces)
	1.36465	Ar136	*Haloquadratum walsbyi* DSM 16790	Square shape and halophilic
EnteroBac	1.11470	Eb37	*Cronobacter universalis* NCTC 9529	Rod-shaped bacteria
	1.25423	Eb22	*Citrobacter murliniae*	Gammaproteobacteria
Fungi	0.86544	Fu44	*Rhodotorula graminis* WP1	Microbotryomycetes
	1.35630	Fu36	*Mycena indigotica*	Bioluminescent fungi
Plant	1.15079	Pl122	*Chlamydomonas reinhardtii*	Single-cell green alga
	1.29509	Pl120	*Zea mays*	Maize (also called "corn")

In Table **S8** a suggestive fact is that both of the extremes of the differences in Animals are in parasitic organisms, a threadworm for the minimum difference and a parasitic mite for the maximum. Also interesting is that for the 122 species of plants studied the minimum difference is for a single-cell green alga, while the maximum difference appears in maize, indicating that this cereal presents the highest coding optimization of the species of plants studied.

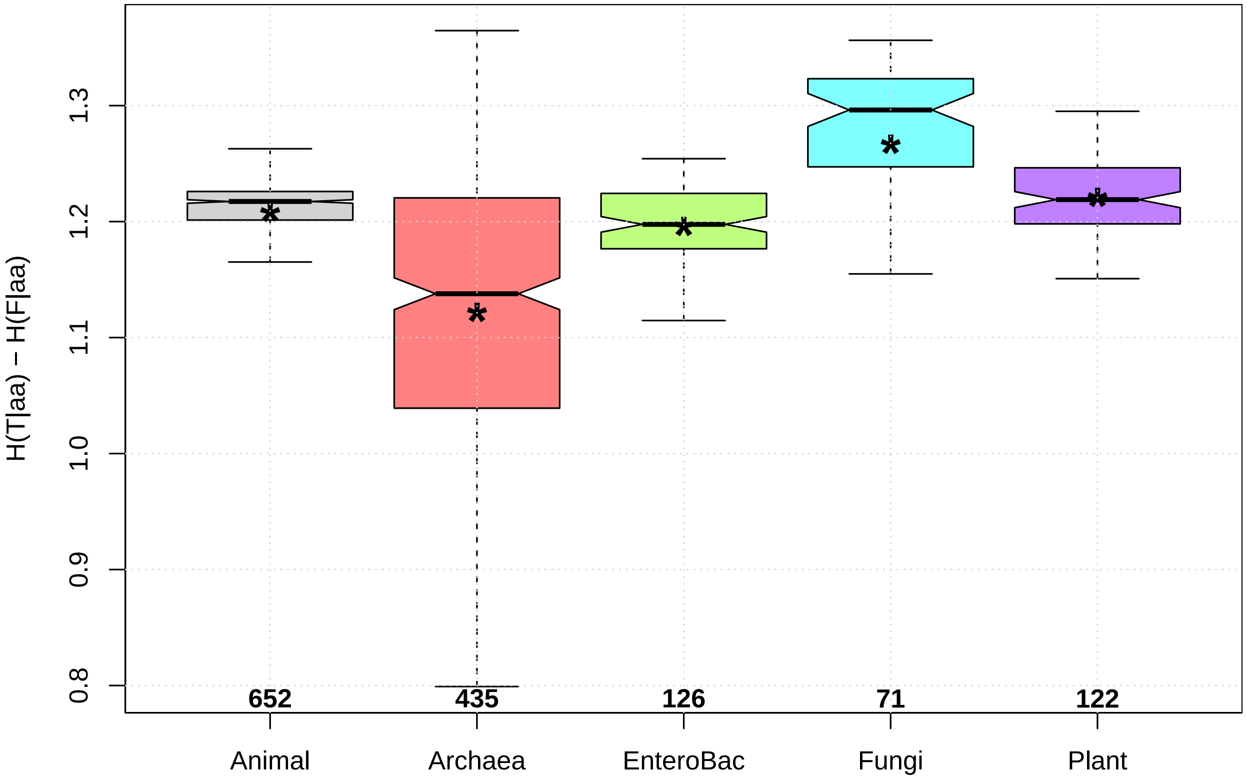

FIGURE **S5**. Distributions as box plots of the values of $H(T|aa) - H(F|aa)$ in the five species groups with more sampled species. Asterisks within each plot point to the position of the mean and numbers in bold at the bottom show the number of species of the corresponding groups; see also Table **S7**.

S4. MUTUAL INFORMATION BETWEEN COMPONENTS OF CODONS

Mutual information between two sets of events, X and Y is defined as

$$I(X;Y) = H(X) - H(X|Y) = H(Y) - H(Y|X)$$

From the previous definition we can infer that mutual information is symmetrical, i.e., $I(X;Y) = I(Y;X)$, and it quantifies the shared average uncertainty between X and Y. Thus, if X and Y are independent we have that $I(X;Y) = 0$.

On the other hand, the maximum value that $I(X;Y)$ happens when $I(X;Y) = \min(H(X), H(Y))$. Thus, for interpretation purposes we can define a quantity that will be always between 0 and 1, say

$$sI(X;Y) = \frac{I(X;Y)}{\min(H(X), H(Y))}$$

Here we refer to "sI" as "*standardized mutual information*", and these quantities quantify the dependency that exist between the two sets of events evaluated. We calculated for all 1,434 species all values of sI, i.e., all values of the 15 possible measures, $sI(aa;FS)$, $sI(aa;FT)$, $sI(aa;ST)$, $sI(aa;F)$, $sI(aa;S)$, $sI(aa;T)$, $sI(FS;FT)$, $sI(FS;ST)$, $sI(FS;T)$, $sI(FT;ST)$, $sI(FT;S)$, $sI(ST;F)$, $sI(F;S)$, $sI(F;T)$ and $sI(S;T)$.

Given that quantities sI vary between 0 and 1, for each one of the 1,434 species studied we can evaluate which of these measurements presented the maximum value. The sI quantity that presents the maximum in each species will tell which of the codon components are more dependent. Table **S9** presents the number of times that a given sI quantity presented the maximum in each species group.

TABLE **S9**. Times that a sI was maximum per group and in total.

Was max ⇓	Group An	Ar	Eb	Fu	Ob	Pl	Pr	Vi	Total
$sI(aa;FS)$	0	65	0	4	0	1	0	0	70
$sI(aa;S)$	652	370	126	67	3	121	4	21	1,364
n species	652	435	126	71	3	122	4	21	1,434
% $sI(aa;S)$	100	85	100	94	100	99	100	100	95

As shown in Table **S9**, only two from the 15 possible sI measuraments, $sI(aa;FS)$ and $sI(aa;S)$, reached the maximum per species in one or more of the 1,434 species studied. This means that the maximum of standardized dependency between codon parts for all species was reached only between aa and, either, the duplet formed by the First and Second bases (FS) or, interestingly, with the Second base (S).

The last row in Table **S9**, "% $sI(aa;S)$", shows the percentage of cases in which $sI(aa;S) > sI(aa;FS)$ and it is intriguing that such fact happens very frequently, in $\approx$ 95% of the cases; $1364/1434 \approx 0.9512$.

Figure **S6** presents the distributions, as box plots, for the 6 sI measurements between amino acids (aa) and other components of the codon.

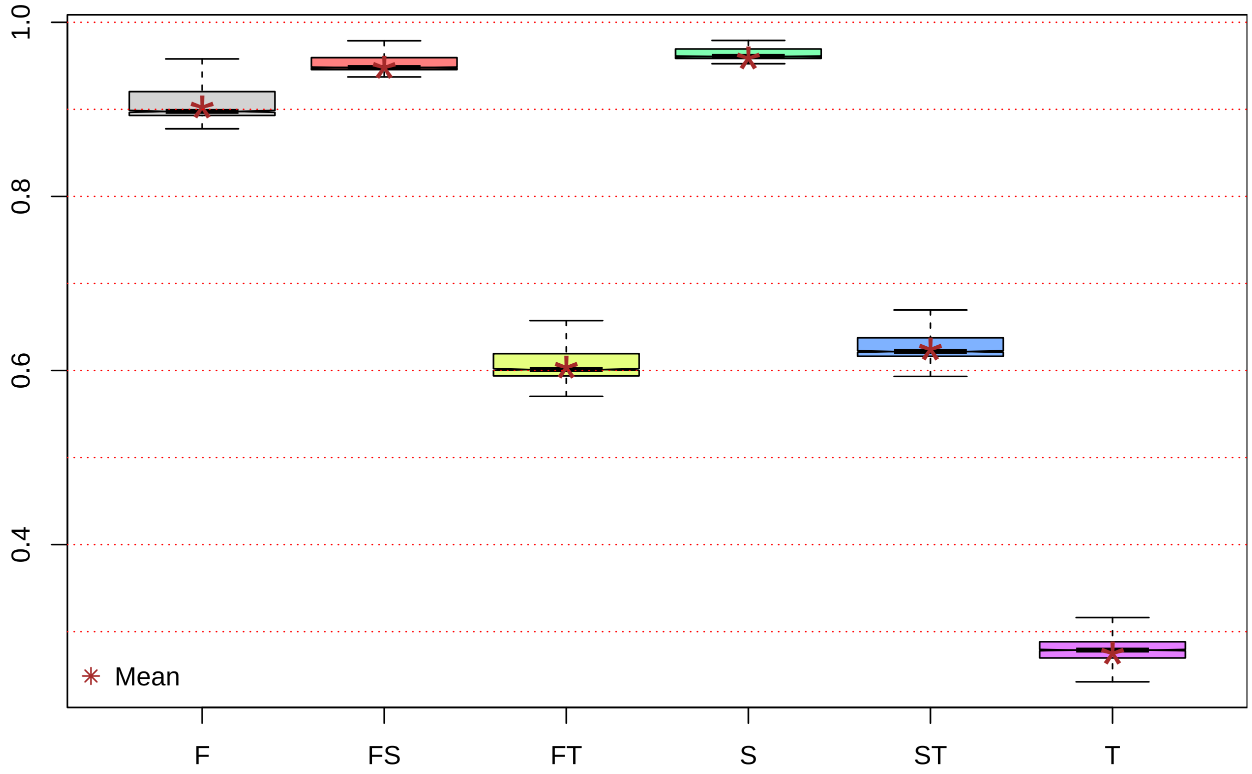

FIGURE **S6**. Distributions as box plots of standardized mutual information measurements, $sI(aa;F)$, $sI(aa;FS)$, $sI(aa;FT)$, $sI(aa;S)$, $sI(aa;ST)$ and $sI(aa;T)$ (second component of the sI is shown as label in the X-axis) in the 1,434 species studied.

In Figure **S6** the first notorious fact is that the 6 distributions of the measurements are highly compact, with a standard deviation, S, that varies between a minimum of 0.00624 for $sI(aa;S)$ up to a maximum of 0.02364 for $sI(aa;T)$ and a median of 0.01707 (data not shown in the plot). In contrast, the central tendencies of those distributions vary widely, from a minimum average of 0.2800 for $sI(aa;T)$, in the

right hand side box plot in Figure **S6**, up to a maximum of 0.9637 for $sI(aa;S)$; means shown as red asterisks in Figure **S6**.

The distributions presented in Figure **S6** are congruent with what is expected from the informational structure of the genetic code; while $sI(aa;F)$, $sI(aa;FS)$ and $sI(aa;S)$ have distributions which are close to the maximum value of 1, the one for $sI(aa;T)$ presents low values of dependency, a fact that is consistent with the secondary role played by the Third row in amino acid determination.

Figure **S7** presents the distributions as box plots of $sI(aa;F)$ in the main groups of species.

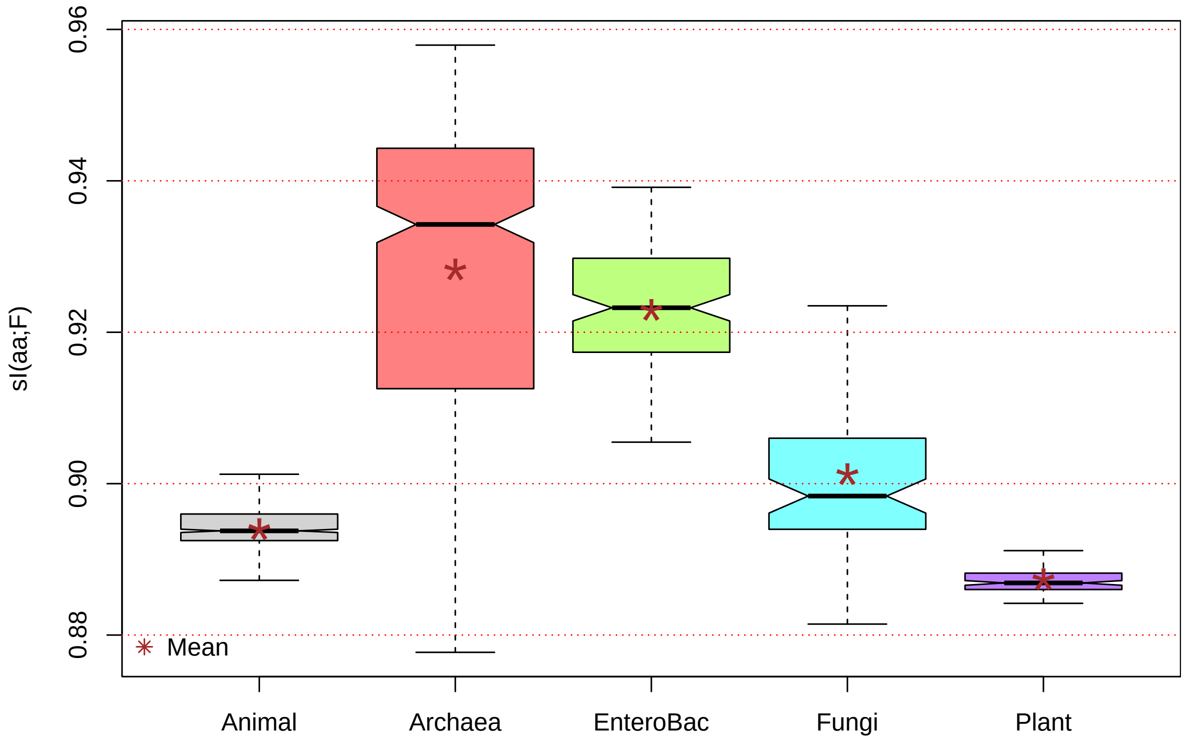

FIGURE **S7**. Distributions as box plots of $sI(aa;F)$ in the main groups of species.

In Figure **S7** we can appreciate how the distribution of $sI(aa;F)$ for Archaea is the one with a larger spread, while the one for Plant is the most compact. For all 5 groups of main species the distributions of $sI(aa;F)$ are almost contained between the limits of 0.88 and 0.96, a fact that corroborates the large dependency between the First base and the amino acid coded.

Figure **S8** presents the distributions as box plots of $sI(aa;FS)$ in the main groups of species, while Figure **S9** presents the distributions as box plots of $sI(aa;S)$ in the same main groups of species.

The distributions in Figure **S8** and Figure **S9** for $sI(aa;FS)$ and $sI(aa;S)$, respectively, are alike in the sense that in both cases the data for Archaea are the most variable while the ones for Plant are the most compact, as it was also observed in Figure **S7** for $sI(aa;F)$.

In Figure **S8** we can observe that the distributions of $sI(aa;FS)$ for the cases with more species sampled are within the range of ≈ 0.9372 and ≈ 0.9788. This demonstrates that the dependence of standardized mutual information of amino acid (aa) with the duplet formed by the First and Second bases is almost as high as it could be, i.e., very close to the full dependency where this measurement will take the value of $sI(aa;FS) = 1$. The distributions in this figure are more symmetric for Animal and EnteroBac and less so for Archaea, Fungi and Plant, where the mean (shown as red asterisks) is less close to the median. Ordering the medians of $sI(aa;FS)$ for the five groups, the smallest is for Plant (0.94226) , followed by Animal (0.94608), Fungi (0.94837), EnteroBac (0.96089) and Archaea (0.96691). These differences

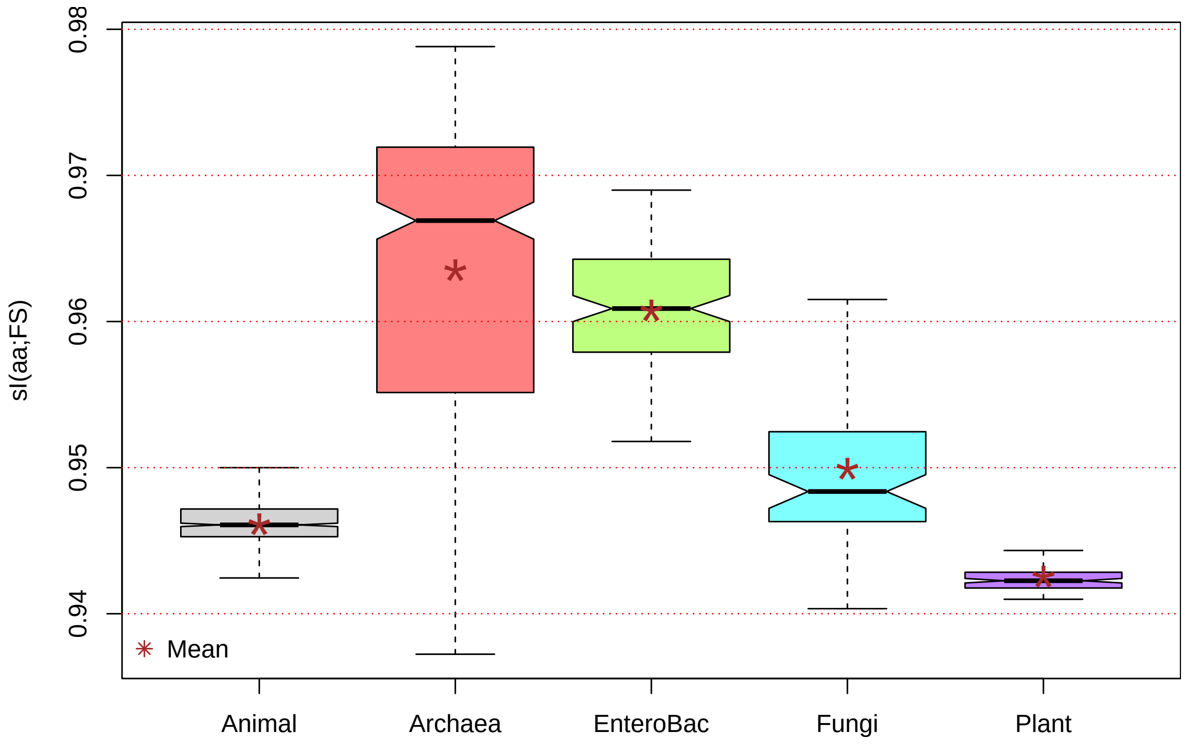

FIGURE **S8**. Distributions as box plots of $sI(aa;FS)$ in the main groups of species.

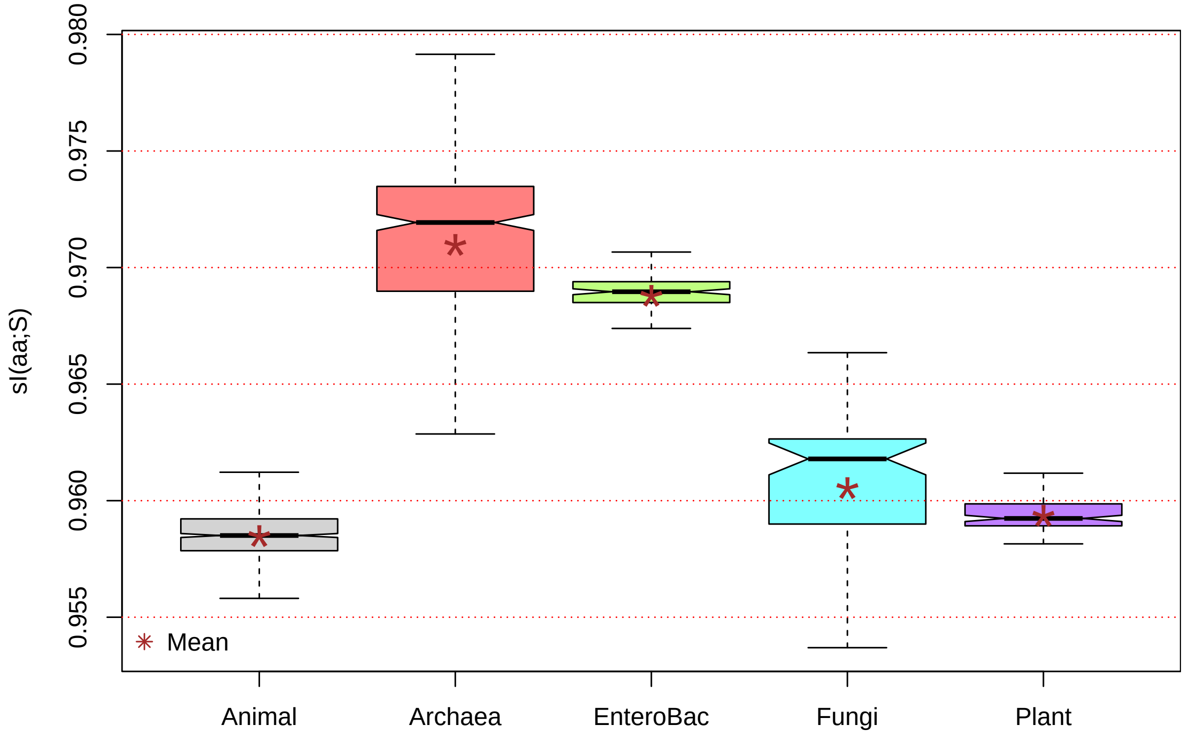

FIGURE **S9**. Distributions as box plots of $sI(aa;S)$ in the main groups of species.

suggest that the evolutionary forces keeping the dependency of aa with FS at high values are taxa dependent.

In Figure **S9** the distributions of $sI(aa;S)$ for the cases with more species sampled are within the range of ≈ 0.9524 and ≈ 0.9791, i.e., higher than the range for $sI(aa;FS)$ shown in Figure **S8**. This is because, as shown in Table **S9**, in approximately 95% of the cases $sI(aa;S) > sI(aa;FS)$, a fact that implies that in the majority of the cases the dependency between aa and S is larger than between aa and FS.

Figure **S10** presents a dot plot and linear model for standardized mutual information $sI(aa;S)$ and $sI(aa;FS)$.

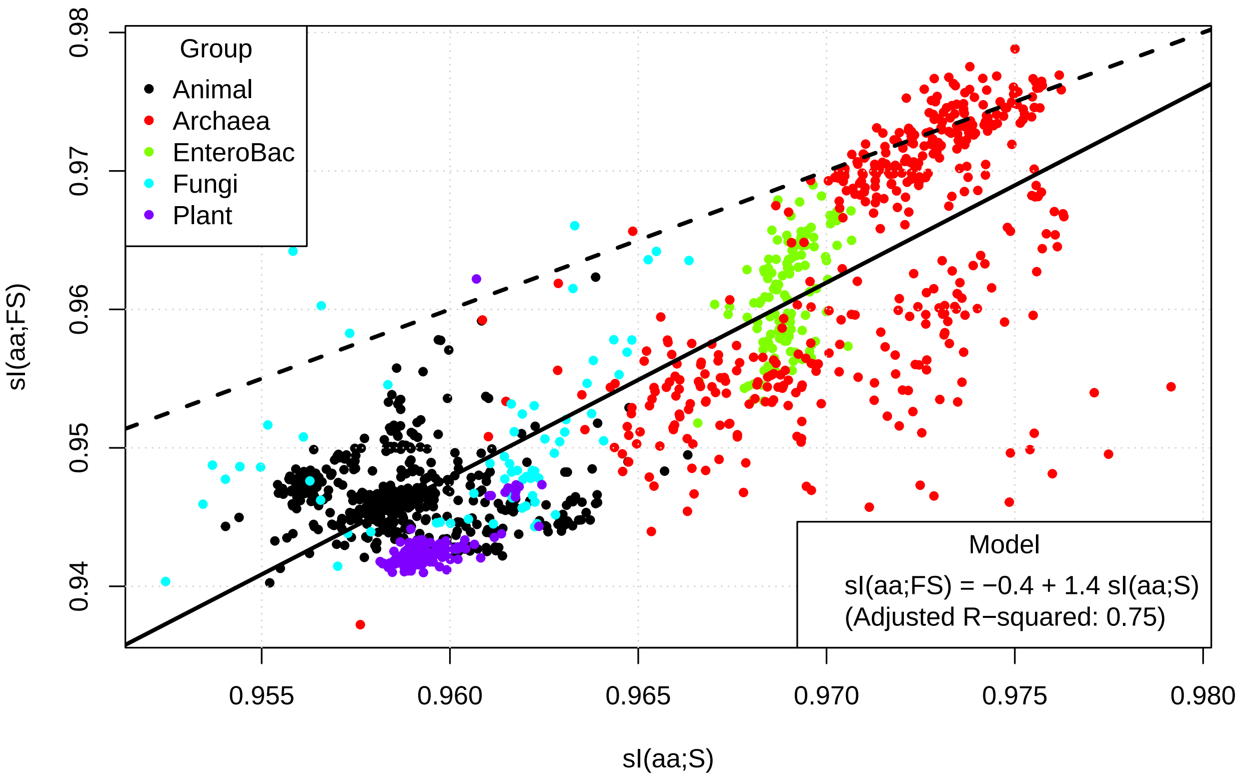

Figure **S10**. Dot plot and linear model (continuous line) for standardized mutual information $sI(aa;S)$ in the X-axis and $sI(aa;FS)$ in the Y-axis. Dashed line at $sI(aa;S) = sI(aa;FS)$.

In Figure **S10** the continuous line presents the estimated linear model $sI(aa;FS) = -0.4 + 1.4sI(aa;S)$, which explains approximately 75% of the variance of $sI(aa;FS)$ as function of $sI(aa;FS)$. Even when the linear relation between $sI(aa;FS)$ and $sI(aa;S)$ is strong and positive, the estimated slope, 1.4, is larger than 1 –the dashed line in the figure, from which we can appreciate that approximately 5% of the points are above this border, and correspond mainly to Archaea (red points), four Fungi (blue points) and a single plant (purple point). This plant outlier has values $sI(aa;FS) \approx 0.9621$ and $sI(aa;S) \approx 0.9607$ and corresponds to the species *Chlamydomonas reinhardtii*, a single-cell green alga (label Pl122, row 1413 in the species matrix), which we found has very different entropy related values compared with the remaining species of the plant group. Except for this outlier, the Plant group presents a highly compact group in the bi-dimensional space $sI(aa;S) \times sI(aa;FS)$. In contrast, the Archaea group species (red points) are highly disperse in the $Is(aa;S) \times sI(aa;FS)$ space, but presents a more compact sub-group in the upper right hand side of the graph. Fungi (light blue points) are also highly dispersed, while Animal (black points) form a more compact group which overlaps to some extent with Fungi and EnteroBac (green points), which forms a relatively compact group which overlaps to some extent with Archaea.

To gain a better understanding of the relations between the 15 standardized mutual information measurements (the sI's) estimated from the 1,434 species, Figure **S11** presents a dendrogram from those quantities.

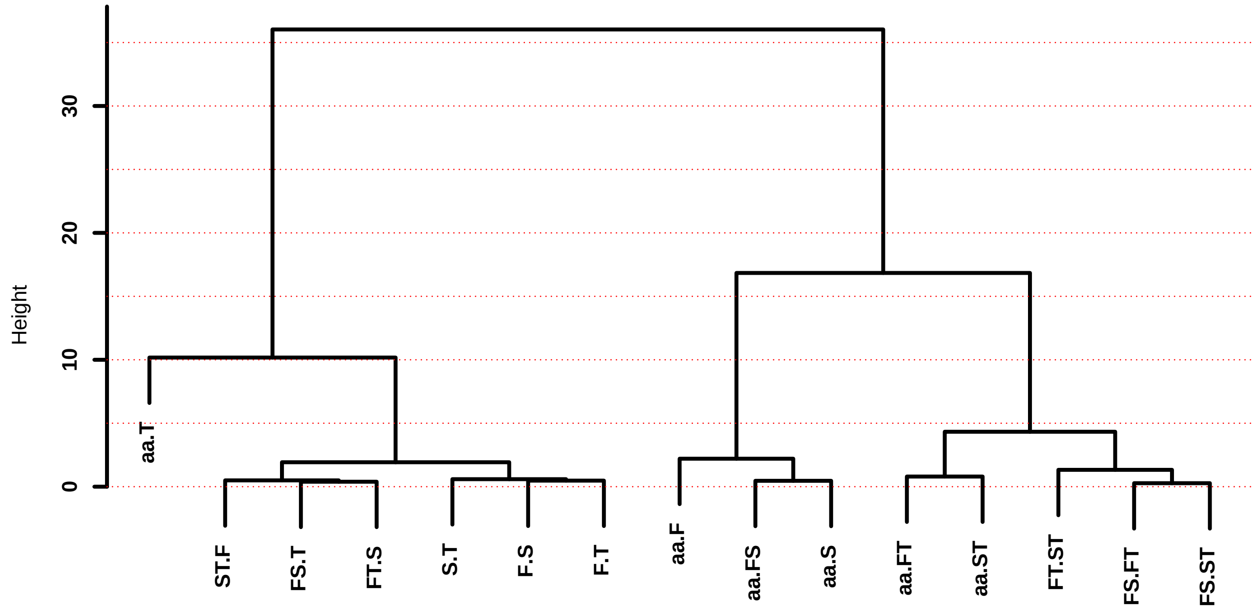

FIGURE **S11**. Dendrogram for the 15 sI measurements in the 1,434 species. Labels are formed by the two terms in the sI; for example $sI(aa;T)$ has label **aa.T**, etc. The dendrogram was obtained by the "complete" method in the function "`hclust()`" using Euclidean distances between sI's.

In Figure **S11** we can see two large clusters separated at a height > 35; the first one of those two clusters, at the left hand side of the dendrogram, includes seven sI's, say $sI(aa;T)$, $sI(ST;F)$, $sI(FS;T)$, $sI(FT;S)$, $sI(S;T)$, $sI(F;S)$ and $sI(F;T)$. All the 7 sI's in this cluster correspond to measurements with low level of dependency, i.e., values of $sI(\cdot;\cdot)$ which medians are smaller than 0.3 Note that the more divergent quantity within this cluster is $sI(aa;T)$, which is clustered with the other 6 at a height of approximately 10, and is the only standardized mutual information that includes aa; in fact the median of $sI(aa;T)$ is 0.2788 (see also Figure **S6**).

The other large cluster in Figure **S11**, at the right hand side of the dendrogram, can be described using curly brackets to show its topology as

$$\{\{sI(aa;F), \{sI(aa;FS), sI(aa;S)\}\}, \{\{sI(aa;FT)\ , sI(aa;ST)\}, \{sI(FT;ST), \{sI(FS;FT), sI(FS;ST)\}\}\}\}$$

and this cluster includes the 8 measurements which have a large median value of $sI(\cdot;\cdot)$, of at least 0.5223, which demonstrates a large dependence between its terms (the left hand side cluster analyzed above has medians smaller than 0.3 for its $sI(\cdot;\cdot)$ components). In fact, the medians of the $sI(\cdot;\cdot)$ included in this cluster are, from the smaller to the largest, $sI(FT;ST) \mapsto 0.5223$, $sI(FS;ST) \mapsto 0.5242$, $sI(FS;FT) \mapsto 0.5254$, $sI(aa;FT) \mapsto 0.6011$, $sI(aa;ST) \mapsto 0.6218$, $sI(aa;F) \mapsto 0.8977$, $sI(aa;FS) \mapsto 0.9480$ and $sI(aa;S) \mapsto 0.9609$, where here the symbol "$\mapsto$" means that we took the median of the corresponding quantity in the results for the 1,434 species.

In Figure **S11** we can see that the clusters

$\{sI(aa;F), \{sI(aa;FS), sI(aa;S)\}$ and

$\{sI(aa;FT)\ , sI(aa;ST)\}, \{sI(FT;ST), \{sI(FS;FT), sI(FS;ST)\}\}\}$

are group together at a high height, > 15, meaning that these two groups are well segregated in the space of the 15 sI's values for the 1,434 species. Measurements $sI(aa; FS)$ and $sI(aa; S)$, for which we gave box plots per group in Figures **S8** and **S9** and that show a large correlation in Figure **S10**, are the ones with the higher medians, and also are grouped together in the cluster $\{sI(aa; FS), sI(aa; S)\}$ at a height very near to 0, indicating that they are very alike quantifications of standardized mutual information.

In summary, the dendrogram in Figure **S11** shows the complex panorama of inter-dependences between standardized mutual informations, $sI(\cdot;\cdot)$, that exist in the species studied. Also, this grouping of the measurements makes perfect sense given the nature of the genetic code.

S5. The complexity of the H's landscape

A total of 54 not-zero and not-redundant H measurements were obtained for each one of the 1,434 species studied, making a total of $1,434 \times 54 = 77,436$ figures to be analyzed. Table **S10** shows the 54 entropy related quantities which constitute the uncertainty profile of each species obtained by the dissection of codon parts (see Table **S2** for the main instances; for the remaining ones please refer to the `ShannonCodonGuide.pdf` document).

Table **S10**. Entropy related measurements obtained for each species.

Class	n	Terms
Single	8	$H(\text{codon})$, $H(aa)$, $H(FS)$, $H(FT)$, $H(ST)$, $H(F)$, $H(S)$, $H(T)$
Join	6	$H(aa, FS)$, $H(aa, FT)$, $H(aa, ST)$, $H(aa, F)$, $H(aa, S)$, $H(aa, T)$
Conditional	25	$H(\text{codon}\|aa)$, $H(\text{codon}\|FS)$, $H(\text{codon}\|FT)$, $H(\text{codon}\|ST)$, $H(\text{codon}\|F)$, $H(\text{codon}\|S)$, $H(\text{codon}\|T)$, $H(aa\|FS)$, $H(FS\|aa)$, $H(aa\|FT)$, $H(FT\|aa)$, $H(aa\|ST)$, $H(ST\|aa)$, $H(aa\|F)$, $H(F\|aa)$, $H(aa\|S)$, $H(S\|aa)$, $H(aa\|T)$, $H(T\|aa)$, $H(FS\|F)$, $H(FS\|S)$, $H(FT\|F)$, $H(FT\|T)$, $H(ST\|S)$, $H(ST\|T)$
Mutual info.	15	$I(aa; FS)$, $I(aa; FT)$, $I(aa; ST)$, $I(aa; F)$, $I(aa; S)$, $I(aa; T)$, $I(FS; FT)$, $I(FS; ST)$, $I(FS; T)$, $I(FT; ST)$, $I(FT; S)$, $I(ST; F)$, $I(F; S)$, $I(F; T)$, $I(S; T)$

In Table **S10** we see the classes, number (n) and terms of entropy related measurements which can be obtained from a single species. The vector of all the 54 measurements give raw values which form what we can call a unique "entropy profile" per species for each one of the species.

We can construct a bifurcating tree (dendrogram) by calculating the Euclidean distances between species from the matrix formed by the vectors of 54 entropy measurements of each species, and then applying a clustering algorithm *via* the `hclust()` function using, for example, the "complete" method. Let's call this dendrogram "$\mathcal{D}_{\mathcal{H}}$" [1].

Alternatively, we can use the matrix of relative frequencies of the 64 codons in each species to estimate a dendrogram for the 1,434 species, again using the Euclidean distance and the "complete" method. Here that dendrogram is called "$\mathcal{D}_{\mathcal{C}}$".

Both dendrograms, $\mathcal{D}_{\mathcal{H}}$ and $\mathcal{D}_{\mathcal{C}}$, classify the same 1,434 species into 1,433 clusters (nodes), but their direct comparison and interpretation is challenging due to the large number of species classified. Thus we used the facilities of our R package `DendroLikeness`, which allows the analysis and comparison of dendrograms.

Clusters within the dendrogram are classified by the number of species that each one of those clusters has, which here we call "`class`" of the cluster, and Table **S11** shows a comparison of dendrograms $\mathcal{D}_{\mathcal{H}}$ and $\mathcal{D}_{\mathcal{C}}$ by detecting and counting the numbers of shared clusters within common classes.

[1] The R steps for estimating dendrogram $\mathcal{D}_{\mathcal{H}}$ are: "`dis.all.species.all.Hs <- dist(species.H.all, method="euclidean")`" (obtains the distance matrix), "`den.com.allSpeAllHs <- hclust(dis.all.species.all.Hs)`" (obtains the dendrogram) and finally obtains the topology with "`topo.den.com.allSpeAllHs <- dend.topo(den.com.allSpeAllHs)`".

Table **S11**. Comparison of dendrograms $\mathcal{D}_{\mathcal{H}}$ (from H values) and $\mathcal{D}_{\mathcal{C}}$ (from relative codon frequencies). Only classes that shared at least one cluster are shown.

`class`	n in $\mathcal{D}_{\mathcal{H}}$	n in $\mathcal{D}_{\mathcal{C}}$	n of comp.	n shared	% shared
2	503	484	243,452	206	42.56
3	260	256	66,560	55	21.48
4	122	132	16,104	16	13.11
5	100	83	8,300	6	7.23
6	70	61	4,270	6	9.84
8	44	40	1,760	2	5.00
11	22	27	594	1	4.55
16	8	15	120	1	12.50
22	5	3	15	1	33.33

First it is relevant to mention that $\mathcal{D}_{\mathcal{H}}$ has 93 different classes (cluster sizes) while $\mathcal{D}_{\mathcal{C}}$ has 96. Clusters between two dendrograms are comparable only when they belong to the same class, i.e., only when they have the same number of elements, and in such case the clusters can be compared to determine if they are identical, i.e., if they consist exactly of the same species. Table **S11** has only 9 rows, this means that only for $11/93 \approx 0.12$ or 12% of the classes the comparisons between dendrograms resulted in at least one pair of identical clusters, implying that the grouping in $\mathcal{D}_{\mathcal{H}}$ compared with the one of $\mathcal{D}_{\mathcal{C}}$ is very different; in fact, even if all the cases presented in Table **S11** were identical clusters, this will give a maximum of 12% of likeness between $\mathcal{D}_{\mathcal{H}}$ and $\mathcal{D}_{\mathcal{C}}$.

However, Table **S11** shows that the percentages of shared clusters (identical clusters in $\mathcal{D}_{\mathcal{H}}$ and $\mathcal{D}_{\mathcal{C}}$) goes from a minimum of 4.55% for clusters of size (`class`) 11 up to a maximum of 42.56% for the smaller clusters of `class` = 2 in the first row of the table. This first row shows that there are 503 clusters of size 2 in $\mathcal{D}_{\mathcal{H}}$, while in $\mathcal{D}_{\mathcal{C}}$ this figure is 428. By performing all the $503 \times 428 = 215,284$ pair comparisons between those clusters (column "n of comp." in Table **S11**) we found that only 206 of those clusters were identical, giving a proportion of $206/484 \approx 0.4256$ or 42.56% in column "% shared" of the table. The median of the values in column "% shared" is 12.5, thus we can roughly estimate that likeness between dendrograms $\mathcal{D}_{\mathcal{H}}$ and $\mathcal{D}_{\mathcal{C}}$ is of only 1.5%; $100 \times 0.125 \times 0.12 = 1.5$, by multiplying the median percentage of shared by the proportion of comparable classes.

In summary, from the data presented in Table **S11** we conclude that clustering of species by H values in $\mathcal{D}_{\mathcal{H}}$ is mostly different to the one given by clustering by relative frequency of codons in $\mathcal{D}_{\mathcal{C}}$. This reinforce the conclusions previously presented when analyzing the results in Figures **S2** and **S3**, demonstrating that the study of entropy measurements provides a very different panorama of species relations than the simple study of codon relative frequencies.

Now we will focus our attention in the relations between taxonomic groups found in the $\mathcal{D}_{\mathcal{H}}$ dendrogram. In $\mathcal{D}_{\mathcal{H}}$ we found 93 classes, the most numerous was the `class=2` which has 503 clusters, meaning that there are 503 clusters which include only one pair of species. The next, `class=3` has 260 elements, `class=4` has 122 elements, up to the largest cluster, `class=1434`, which obviously has a single element which group all species. A way to measure how much the dendrogram reflects the taxonomical classification implicit in the 8 species groups (see Table **S1**) is to count the number of clusters of each `class` which are formed by species of the same group. For each class of the dendrogram we obtain the proportion of consistency of the clusters in that class by dividing the number of clusters formed by members of the same group by the total number of clusters in the class. Of the 93 classes found in $\mathcal{D}_{\mathcal{H}}$, 53 ($\approx 57\%$) presented consistent clusters, i.e., clusters formed exclusively by species of the same group. The estimated linear correlation between the proportion of consistent clusters and the median of the height found in the dendrogram was $\hat{r} \approx -0.4307$, indicating that consistent clusters tend to have a small median height within the dendrogram.

Table **S12** presents the number and percentages of consistent clusters in the dendrogram $\mathcal{D}_{\mathcal{H}}$ taking into account only classes which have at least one consistent cluster.

TABLE **S12**. Number of clusters (n Clu.), number of consistent clusters (n Con.), percentage of consistency (% Con.) and median height of the clusters (Median height) per `class` in dendrogram $\mathcal{D}_{\mathcal{H}}$.

`class`	n Clu.	n Con.	% Con.	Median height
2	503	495	98.41	0.0213
3	260	248	95.39	0.0350
4	122	115	94.26	0.0444
5	100	95	95.00	0.0423
6	70	60	85.71	0.0912
7	47	40	85.11	0.0885
8	44	39	88.63	0.0834
9	21	20	95.24	0.0815
10	25	20	80.00	0.1615
11	22	15	68.18	0.1029
≥ 12	174	127	73.00	0.1479

In Table **S12** we can appreciate how the percentage of consistency is $\geq 95\%$ for the clusters of sizes 2 to 5 (`class` = 2, 3, 4 and 5) and it does not decrease very much for clusters of larger sizes; the minimum, 73%, is found for lusters of relative large sizes (`class` ≥ 12; last row in the table). We can also notice that the median of the height of the clusters per class (column "Median height" in Table **S12**) tends to be higher for larger classes, but also presents an inverse relation with the proportion of consistent clusters ($\hat{r} \approx -0.4307$, presented above).

Figure **S12** presents the histogram of the proportion of consistency in the 93 classes in dendrogram $\mathcal{D}_{\mathcal{H}}$.

In Figure **S12** we can see that in 40 of the 93 clases in dendrogram $\mathcal{D}_{\mathcal{H}}$ (43% of the clases) the proportion of consistency is very low, between 0 and 0.1; first bar at the left hand side. This coincides with clusters formed at a large height in the dendrogram (median height 0.49, number in green within the corresponding bar plot). In contrast, high values of proportion of consistency, between 0.95 and 1.00, are present in 27% of the classes formed at a low median height of 0.11; last bar in the right hand side of the figure.

In summary, from Table **S12** and Figure **S12** we conclude that small distances in the 54-dimensional space of the H values give a high proportion of clusters that are taxonomically consistent with the groups of species defined in this study.

To gain a better understanding of the complexity of the 54-dimensional space of the H values, Figure **S13** presents a dendrogram of consistent clusters of `class` = 2 within groups, and the key for the species of that dendrogram are presented in Table **S13**.

The dendrogram in Figure **S13** was constructed by selecting the consistent clusters of `class` = 2 of clusters of species from the same group which presented a minimum height for that class in dendrogram $\mathcal{D}_{\mathcal{H}}$. First, it is important to notice that the dendrogram in Figure **S13** does not have species from the groups Pr (Protists) or Ob (OtherBac; see Table **S1**); this is due to the fact that there were not clusters of `class` = 2 for those two groups in the dendrogram $\mathcal{D}_{\mathcal{H}}$; that is, species from groups Pr and Ob where grouped first with species from other groups, than with species from their own kind, implying that Pr and Ob are taxonomically less consistent in the H space than the other 6 groups of species represented by two species per group in the dendrogram of Figure **S13**.

Figure **S14** was constructed from the matrix of Euclidean distances in the H space employing the same 12 species shown in the dendrogram of Figure **S13** but adding also the full groups of Protists (Pr), OtherBac

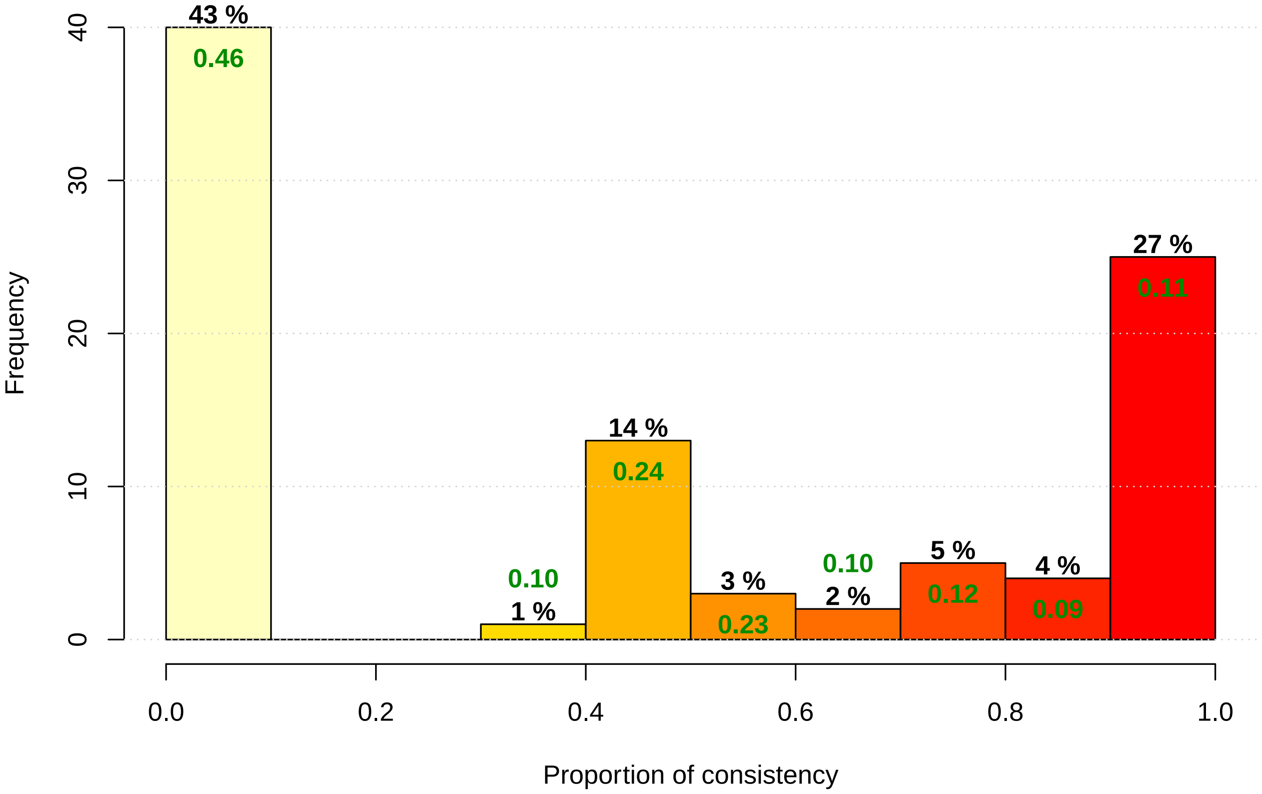

FIGURE **S12**. Histogram of frequencies (Y-axis) per proportion of consistency (X-axis) for the 93 classes of clusters found in dendrogram $\mathcal{D}_{\mathcal{H}}$. Numbers in bold at the top of the bars give the percentages of the frequencies, while numbers in green give the median of the heights of the groups included in each bar.

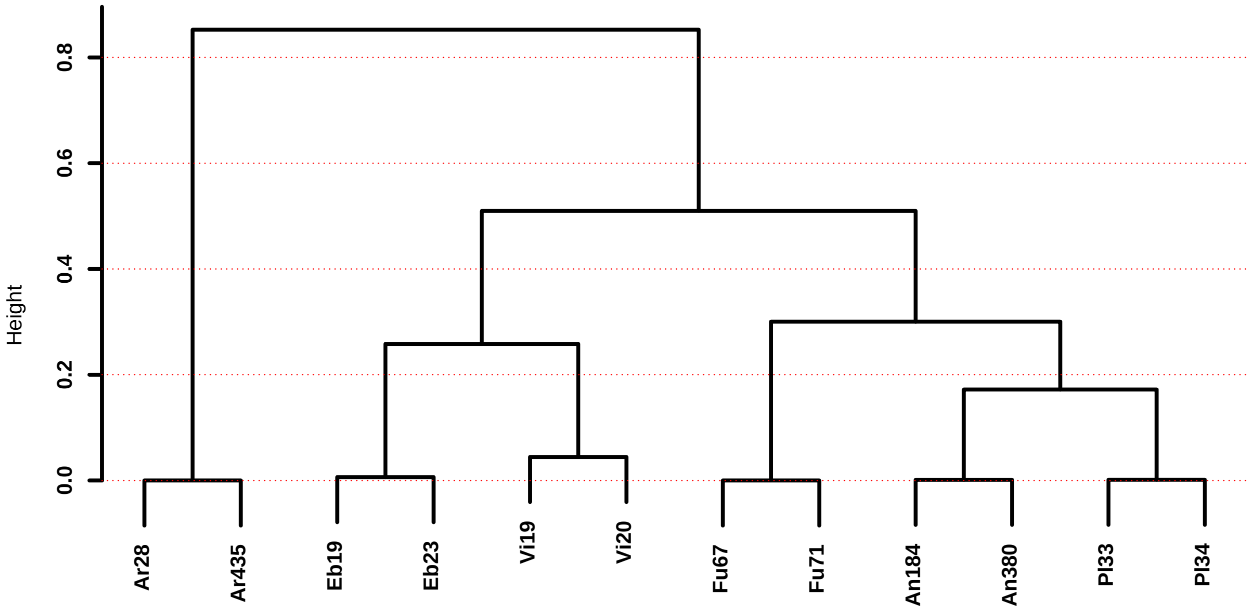

FIGURE **S13**. Dendrogram for the species with smaller distances within group in the space of the 54 H measurements. Dendrogram obtained from Euclidean distances by the "complete" method in the function "`hclust()`".

Table **S13**. Labels of species shown in the dendrogram of Figure **S13**.

Label	`n.row`	Group	Species	Comment
**Ar28**	812	Ar	*Haladaptatus cibarius* D43	Genus of halophilic archaea (particular isolate).
**Ar435**	1220		*Haladaptatus cibarius*	Genus of halophilic archaea (another isolate).
**Eb19**	675	Eb	*Citrobacter freundii*	Facultative anaerobic. Gram-negative bacteria.
**Eb23**	679		*Citrobacter pasteurii*	Bacterium isolated from human feces.
**Vi19**	1432	Vi	West Nile virus	Single-stranded RNA virus, causes West Nile fever.
**Vi20**	1433		Zika virus	Family *Flaviviridae* spread by *Aedes* mosquitoes.
**Fu67**	1287	Fu	*Ustilago maydis* 521	Causes corn smut (in maize); edible, "*huitlacoche*".
**Fu71**	1291		*Ustilago maydis*	Another isolate from the previous species.
**An184**	188	An	*Delphinapterus leucas*	The beluga (or "white") whale; cetacean.
**An380**	384		*Monodon monoceros*	The narwhal; species of toothed whale; cetacean.
**Pl33**	1324	Pl	*Cucurbita moschata*	Squash; edible flesh, flowers, greens, and seeds.
**Pl34**	1325		*Cucurbita pepo subsp. pepo*	Summer squash; domesticated in the Americas

`n.row` – Number of row in the species matrix.
Group – Ar = Archaea, Eb = EnteroBac, Vi = Virus, Fu = Fungi, An = Animal, Pl = Plant.

(Ob) and Virus (Vi). Table **S14** presents the species shown in the dendrogram of Figure **S14**, but not in the dendrogram of Figure **S13**.

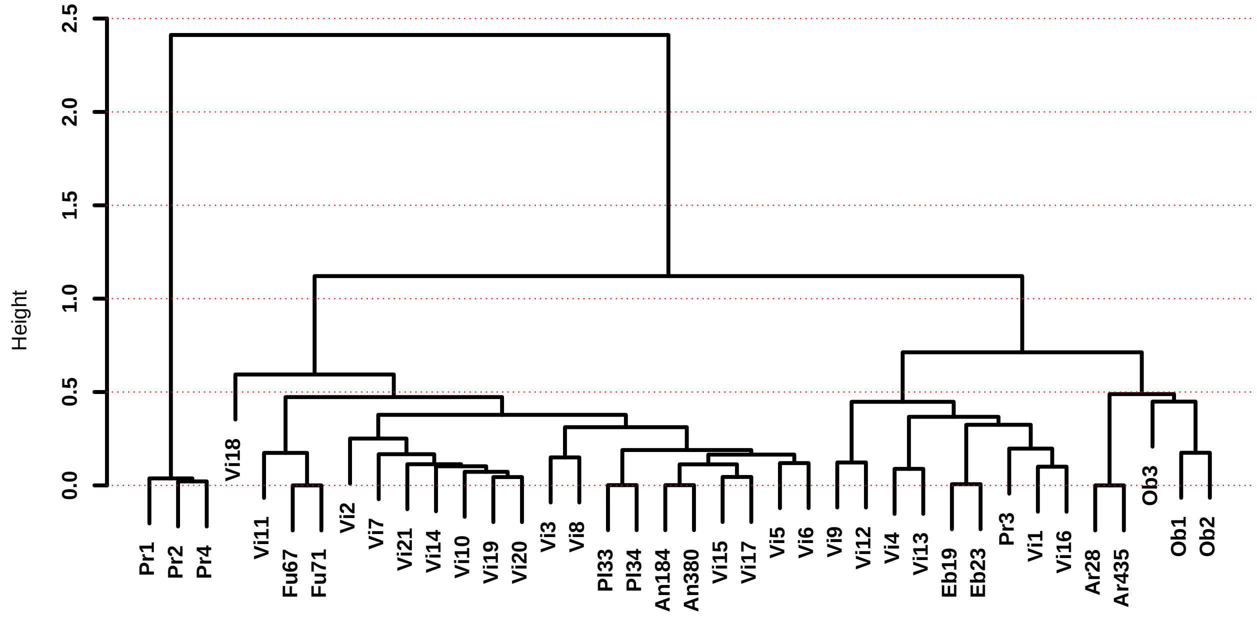

Figure **S14**. Dendrogram as the one in Figure **S12**, but adding all missing species of Pr (Protist), Ob (OtherBac) and Vi (Virus). See tables **S13** and **S14** for species names. Dendrogram constructed from Euclidean distances in the H space by the "complete" method.

A first notorious difference between the dendrograms in Figure **S13** with the one in Figure **S14** is the increase in the maximum height reached. While in Figure **S13** the maximum height is of around 0.82 between the two Ar and the other species, in Figure **S14** the maximum height is much higher, a little less that 2.5, and the cluster including three Protist (Pr1, Pr2 and Pr4) is the one that reached that maximum, segregating these 3 species from all the others shown.

TABLE **S14**. Extra species shown in the dendrogram of Figure **S14**, but not in the dendrogram of Figure **S13**.

Label	Group	Species	Comment
Pr1	Pr	*Entamoeba dispar* SAW760	harmless amoeba
Pr2		*Entamoeba histolytica* HM-1:IMSS	anaerobic parasitic amoeba
Pr3		*Entamoeba invadens* IP1	amoeba parasite of reptiles
Pr4		*Entamoeba nuttalli* P19	amoeba prevalent in macacos
Ob1	Ob	*Elusimicrobium minutum* Pei191	ultramicrobacterium; obligately anaerobic
Ob2		*Endomicrobium proavitum*	ultramicrobacterium; fixes nitrogen
Ob3		*Thermococcus barophilus*	piezophilic and hyperthermophilic archaeon
Vi1	Vi	*Cyrtanthus elatus* virus A	virus isolated from Narcissus (a plant)
Vi2		Enterovirus C	species of enterovirus; cause of poliomyelitis
Vi3		Hippeastrum mosaic virus	Potyvirus (a plant mosaic virus)
Vi4		Human immunodeficiency virus 1	HIV-1 virus causes human HIV
Vi5		Human mastadenovirus A	virus of family Adenoviridae (Humans are hosts)
Vi6		Human mastadenovirus B	Other type of previous species
Vi7		Japanese encephalitis virus	family Flaviviridae; known to infect humans
Vi8		Marburg marburgvirus	cause Marburg virus disease in humans a viral hemorrhagic fever
Vi9		Middle East respiratory syndrome-related coronavirus	causes a respiratory syndrome (MERS); coronavirus infects humans, bats, and camels
Vi10		Murray Valley encephalitis virus	(MVEV) a zoonotic flavivirus endemic to Australia and Papua New Guinea
Vi11		Pegivirus A	positive-stranded RNA virus in the family Flaviviridae
Vi12		SARS coronavirus	Severe acute respiratory syndrome coronavirus 1
Vi13		Simian immunodeficiency virus	retrovirus cause persistent infections in at least 45 species of non-human primates
Vi14		Spondweni virus	arbovirus (arthropod-borne virus), family Flaviviridae
Vi15		Sudan ebolavirus	virological taxon included in the genus Ebolavirus, family Filoviridae, order Mononegavirales
Vi16		Sweet potato virus 2	Potyvirus causal agents of sweet potato viral disease (SPVD)
Vi17		Tai Forest ebolavirus	virological taxon included in the genus Ebolavirus, family Filoviridae, order Mononegavirales
Vi18		Tomato leaf curl Kumasi virus	begomoviruses causing tomato leaf curl disease
Vi21		Zika virus 2	virus family Flaviviridae spread by daytime-active Aedes mosquitoes

Group “Pr” are Protist (amoebas), “Ob” are OtherBac and “Vi” are Virus; see Table **S1**.

In Figure **S14** Virus (Vi) are scattered through all groups formed at a height < 1, without a marked preference to be clustered with other groups. This could be due to the fact that entropy measurements in the H space for virus are strongly affected by each virus host.

Also in Figure **S14** we see that the 3 species of OtherBac (Ob), form a cluster, {Ob3, {Ob1, Ob2}}, that at higher height is grouped with the two Archaea, {Ar28, Ar435}. The cluster {{Ar28, Ar435}, {Ob3, {Ob1, Ob2}}} –at the right hand side of the dendrogram, is well separated from the one formed by the two EnteroBac (Eb), {Eb19, Eb23}, implying that in the H space the Ob species are closer to Ar than to Eb. This is not surprising, given that Ob3 has been recently re-classified in the domain of the Archaea (see Table **S14**).

A surprising fact happens with the Protist (Pr; amoebas) in Figure **S14**. Three of those species form a highly compact group, {Pr1, {Pr2, Pr4}}, at the left hand hand side of the dendrogram. These three

species are Pr1 – *Entamoeba dispar* (a harmless amoeba), Pr2 – *Entamoeba histolytica* (a parasite of humans), and Pr4 – *Entamoeba nuttalli* (an amoeba prevalent in macacos). However, the other species of Pr, Pr3 – *Entamoeba invadens* (amoeba parasite of reptiles) is completely segregated and appears clustered with two viruses, Vi1 and Vi16, and then with the two EnteroBac, Eb19 and Eb23. This implies that entropy measurements for Pr3 are highly divergent to the ones in the other three protists, {Pr1, {Pr2, Pr4}} and in turn this could be due to differences in the evolutive path caused by strong and ancient differences in life style and/or hosts.

S5.1. Studying distances within and between species groups. When using distances either, in the 54-dimensional space of the H measures, or in the 64-dimensional space of the 64 relative codon frequencies, we estimated more than a million of pair distances between species for each space; this figure arises from the calculation $1,434 \times (1,434-1)/2 = 1,027,461$. The estimated Pearson's correlation coefficient between distances obtained from H's and the ones obtained from the relative codon frequencies was $\hat{r} \approx 0.7582$, with a 99% confidence interval with limits 0.7571 and 0.7593, for the lower and upper limits, respectively. The determination coefficient between those two variables was $\hat{r}^2 \approx 0.5749$, thus only around 58% of the variation of distances from H can be explained by the distances from the relative codon frequencies. Table **S15** presents the main statistics for the pair distances between species.

TABLE **S15**. Statistics for pair of distances.

**I** – All the 1,027,461 pair of distances between species by source.						
Source ⇓	Min.	Median	Mean	Max.	S	CV
H's (54)	0.00000	0.53013	0.75561	2.96778	0.63401	0.83907
Codon relative frequencies (64)	0.00000	0.08033	0.08483	0.26206	0.04204	0.49558
Times H larger		7	9	11	15	2
**II** – Medians of distances within groups by source (8 instances).						
Source ⇓	Min.	Median	Mean	Max.	S	CV
H's (54)	0.00388	0.33334	0.32987	1.84826	0.20867	0.77553
Codon relative frequencies (64)	0.00069	0.04770	0.05106	0.14240	0.02733	0.55227
**III** – Medians of distances between groups by source (28 instances).						
Source ⇓	Min.	Median	Mean	Max.	S	CV
H's (54)	0.13621	0.74949	0.79670	2.37141	0.37100	0.44205
Codon relative frequencies (64)	0.04460	0.10255	0.10263	0.19009	0.02440	0.28545

In section **I** of Table **S15** the third row, "Times H larger", shows the rounded number of times that each one of the corresponding statistics is larger in the distances from H's than in the ones from codon relative frequencies. For example for the mean we have $0.75561/0.08033 \approx 9$, etc. *Per se* the figures in row "Times H larger" are not surprising, given that the scales of the measurements are very different; however it is interesting to notice that the values of the Coefficient of Variation, column "CV" (S/mean), is almost twice as larger for the cases of the distances from the H's compared with the ones from codon relative frequencies. This implies that distances obtained from H's are more discriminant than the ones obtained from codon relative frequencies, i.e., distances from H's tend to give a clearer segregation of species, that as we have seen produce very different grouping when estimating dendrograms.

The distances between pairs of species can be classified as distances "within" group, when both species belong to the same group, or "between" groups when the two species belong to different groups.

Given that we have 8 groups of species (see Table **S1**), we need to evaluate 8 instances within groups. To summarize the general statistics of the distances within groups we used the median of each one of the statistics per group; for example, having the 8 values of the minima within each one of the groups we took the median of those 8 minima to be presented in section **II** of Table **S15** (section "**II** – Medians of distances within groups by source (8 instances)."). On the other hand, there are $8 \times (8-1)/2 = 28$ different pairs of groups. Taking the medians of the corresponding statistics we evaluated distances

between groups of species and results are in section "**III** – Medians of distances between groups by source (28 instances)." in Table **S15**.

For all statistics presented in sections **II** and **III** in Table **S15**, we have that the values from the source (row) "H's (54)" are larger than the corresponding values in the source (row) "Codon relative frequencies". As in section **I**, this is due to the scale difference between the two sources.

By comparing the statistics for source "H's (54)" within (section **II** in Table **S15**) with the ones between groups (section **III** in the same Table) we see that "Min.", "Median", "Mean", "Max." and "S" are larger between than within groups, indicating a coherent segregation of groups of species when using distances from H measures, i.e., statistics for distances between groups are larger than for within groups. However, for the Coefficient of Variation (column "CV") the value is 0.77553 for distances within groups and smaller, 0.44205, for distances between groups suggesting that the the standardized variation is larger within than between groups, possibly due to extreme values within some of the groups.

By making an homologous comparison of statistics for source "Codon relative frequencies (64)" we have approximately the same panorama; "Min.", "Median", "Mean" and "Max." are larger between (section **III**) than within groups (section **II**), indicating that using codon relative frequencies in general gives a good segregation by groups of species.

Figure **S15** presents distributions as box plots of pairs of distances between species.

Panels (A) and (B) of Figure **S15** present the distributions of pairs of distances measured from H's or codon relative frequencies, respectively. Given that the number of species per group vary greatly, comparisons between groups for the same measurement are not similarly representative; for example, while for Archaea (Ar) we have 435 species and thus there are $435 \times (435 - 1)/2 = 94,395$ pairs of distances in the distribution, for Virus (Vi) we have only 21 species, and thus only $21 \times (21 - 1)/2 = 210$ pairs of distances in the distribution, etc. Nevertheless, for groups represented by a relative large number of species as Animal (An, $n = 652$) Archaea (Ar, $n = 435$), EnteroBac (Eb, $n = 126$) and Plants (Pl, $n = 122$; see Table **S1** for other values of n), we can make relatively fair comparisons. In panel (A) we can see that the distributions for An and Pl are more compact and have smaller means and medians than the ones for Ar and Eb, denoting a less variable landscape of H distances for those eukaryotes (An and Pl) than for Ar or Eb. In contrast, panel (B), for distances calculated from relative codon frequencies, shows that the most compact distribution happens for Eb, followed by Pl and then for An, with the distribution for Ar is, as in panel (A), is the less compact and the one that presents the largest variation range.

As mentioned above there are there are $8 \times (8-1)/2 = 28$ different pairs of groups. Given that presenting the 28 box plots for the distribution of pairs of distances will be too cumbersome, we decided to present only the distributions for distances from H values in the 8 pair of groups which include Ar (Archaea) and each one of the other 7 groups of species. Those box plots are presented in panel (C) of Figure **S15**. This also makes sense because, as seen in panel (A) of the same figure, the distribution of distances within Ar is the most variable one.

In panel (C) of Figure **S15** we can see that the distributions of pairs of distances between Ar and each one of the other 7 groups are not very different, having alike compactness (by observing the boxes with interquartiles) and also similar central tendency (medians and means). The means and medians for the cases of Eb and Ob (representing distances between Ar and those two groups, respectively) are the only ones that are below a distance of 1, indicating a relatively closeness with Ar, but in all the other 5 cases means and medians are well above 1, indicating that Ar is very well segregated by H distances, from those 5 groups.

S5.2. The largest distances between species in the H space. We have seen that neither, distances from H nor from codon relative frequencies give a fully congruent taxonomic segregation of the groups of species studied. In Figure **S13** we obtained a dendrogram with the pairs of species with smaller distances

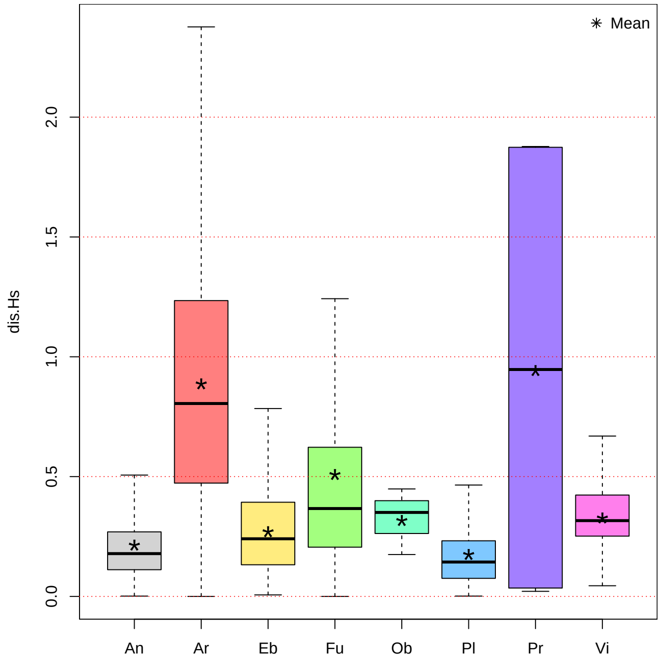

(A) From H's within group.

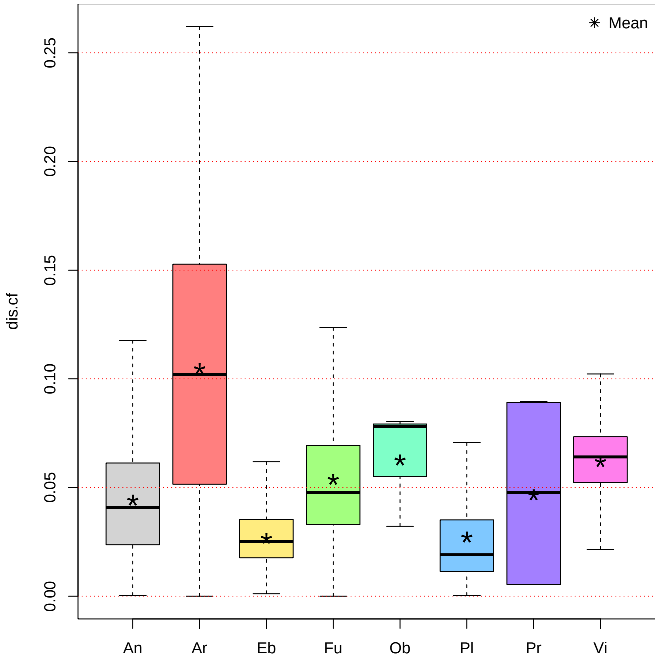

(B) From codon frequencie within group.

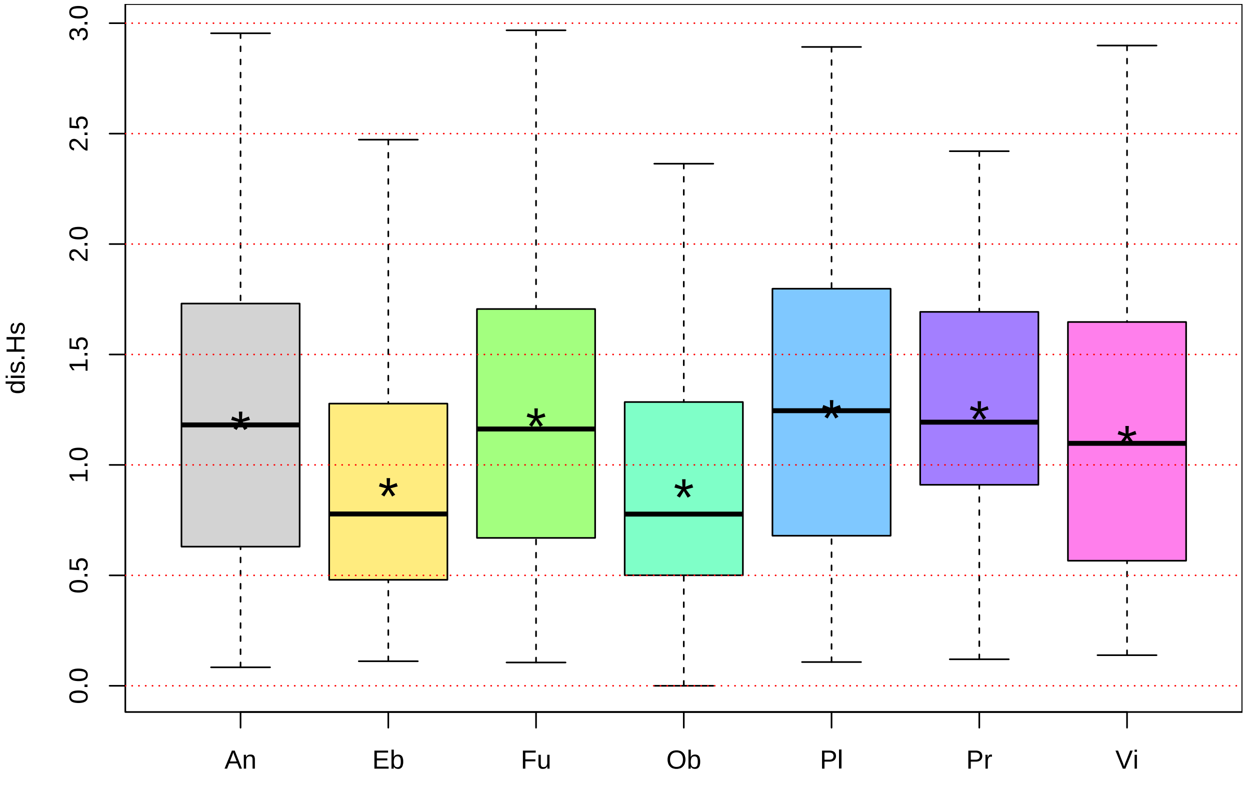

(C) From H's, between "Ar" and each one of the other 7 groups (in the labels).

FIGURE **S15**. Distributions as box plots of pairs of distances between species (see Table **S1** for labels).

within the main species groups. Here we will explore the other extreme, that is, we explore the species that are at a largest distances in the H's space.

For each one of the 8 species groups in turn, we selected (from the set of all H distances) the largest distance which included one member of the group, without taking into account if the other member of the distance pair was or not in that target group. This procedure yielded a set of 10 species representing all the largest distances in the H space and included at least one species from each one of the 8 groups. Only as a reference point, we included our own species in the group of selected species. The resulting dendrogram is presented in Figure **S16**, while Table **S16** lists the species included in that dendrogram.

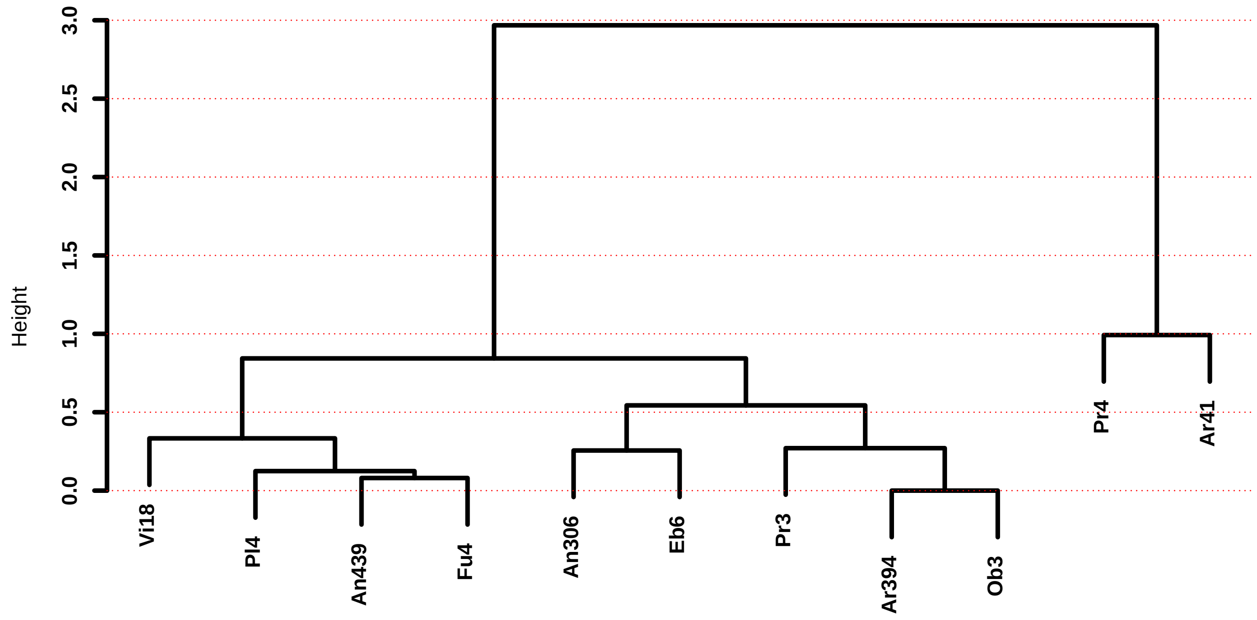

FIGURE **S16**. Dendrogram for the species with larger distances between species pairs in the 54 dimensional space of H values (humans included only as a reference point). Dendrogram obtained from Euclidean distances by the "complete" method of the function "`hclust()`".

In Figure **S16** we can note that the height shown in the Y-axis goes from 0 up to 3, and this range is much larger than the ones obtained in Figure **S13** which used minimal distances between pairs of species of the same group (maximum a slightly larger than 0.8), and also the maximum height of 3 is much higher that the maximum height shown in the dendrogram per species group using medians (Figure **S3**), which is approximately 0.17 This confirms that the species shown in Figure **S16** are outliers in the H space (this fact does not include humans, which is presented only as a reference). In fact, none of the four binary clusters shown in Figure **S16**, {An439, Fu4}, {An306, Eb6}, {Ar394, Ob3} or {Pr4, Ar41} are formed by species of the same group. This demonstrates again that the H distances are not fully taxonomically congruent; species from different groups are linked together when maximum distance in the H space is used.

In Table **S16** we can see that, excepting humans in group An, only Archaea (Ar) and Protist (amoebas, Pr) are represented in this group of outliers by more than one species; Eb, Fu, Ob and Vi are represented by a single species. This is consistent with the distributions presented in panel (A) of Figure **S15**, which shows that Ar and Pr are the most variable groups regarding H distances.

The complete lack of taxonomic consistency shown in the dendrogram of Figure **S16** illustrates the complexity of the H space through the tree of life. By detecting the largest distances in that space we selected a set of 10 outlier species, that excepting humans (An306 selected as reference), are far apart of their own groups and relatively closer to species in very different groups. In Table **S16** we can see

TABLE **S16**. Species shown in the dendrogram of Figure **S16**.

Label	`n.row`	Group	Species	Comment
An306	310	An	*Homo sapiens*	A very strange species indeed!
An439	443		*Opisthorchis viverrini*	Southeast Asian liver fluke, trematode that infects the bile duct
Ar394	1178	Ar	*Thermococcus barophilus* MP	piezophilic and hyperthermophilic archaeon
Ar41	825		*Halarchaeum rubridurum*	halophilic archaea in the family Halobacteriaceae
Eb6	662	Eb	*Buttiauxella gaviniae* ATCC 51604	bacterium isolated from a snail
Fu4	1224	Fu	*Cantharellus anzutake*	edible mushroom native to Japan and Korea
Ob3	1219	Ob	*Thermococcus barophilus*	piezophilic and hyperthermophilic archaeon
Pl4	1295	Pl	*Ananas comosus*	pineapple; a tropical plant
Pr3	3	Pr	*Entamoeba invadens* IP1	amoeba parasite of reptiles
Pr4	4		*Entamoeba nuttalli* P19	amoeba prevalent in macacos
Vi18	1431	Vi	Tomato leaf curl Kumasi virus	begomoviruses causing tomato leaf curl disease

Group “An” Animal, “Ar” Archaea, “Eb” EnteroBac, “Fu” Fungus, “Ob” are OtherBac, “Pl” Plant, “Pr” Protist, and “Vi” Virus; see Table **S1**.

that for animals the outlier is An436, a trematode that infects the bile duct, and which surprisingly is joined first with a fungus, Fu4, which is an edible mushroom from Japan and Korea. The two Archaea, A394 and Ar41 are linked first with Ob3, now classified as a hyperthermophilic archaeon, and to Pr4, an amoeba prevalent in macacos, respectively. On the other hand the representative of plants in the dendrogram, Pl4, is the the pineapple, which happens to be an outlier for that group and is linked first to the cluster formed by an animal, An439, the trematode, and the fungus, Fu4, an edible mushroom. Surprisingly our own species (An306) is clustered first in this dendrogram with a hugely distant species, Eb6, a bacterium.

Following the same procedure described for the H distances, we selected the maximum distances in the space of the codon relative frequencies. Results are presented in Figure **S17** and Table **S17**.

By selecting the maximum distances in the space of the codon relative frequencies we obtained the dendrogram shown in Figure **S17**, which groups 13 species. In this dendrogram 5 species, An306, Ar394, Ob3, Pr3 and Pr4 are also shown in the dendrogram of Figure **S16**, meaning that 4 of those species (excluding humans, * An306) are outliers in both, the space of H's as well as in the one from codon relative frequencies. This in turn suggests that species Ar394, Ob3, Pr3 and Pr4 have had a very peculiar evolutive path that has departed far from the one followed by other species in their respective group.

On the other hand, in Figure **S17** we have (aside of humans) two representative of the animals, An512 (an ocean organism) an An587 (a nematode). In both cases those species are clustered first to groups different to animals, while humans (An306) is clustered with a group already containing species from 4 different groups. One of those groups is the virus, which is represented by Vi4, the HIV-1 human virus, suggesting that the relative codon frequencies in this species has more in common with its human host than with other viruses. In this dendrograms plants are represented by Pl122, a single-cell green alga, which is clustered together with an animal, An512 (a species of barnacle) and Eb106 a gram-negative bacteria. As before in Figure **S16**, the complete lack of taxonomic consistency shown in the dendrogram of Figure **S17** illustrates the complexity of the relative codon frequency space in the species sampled.

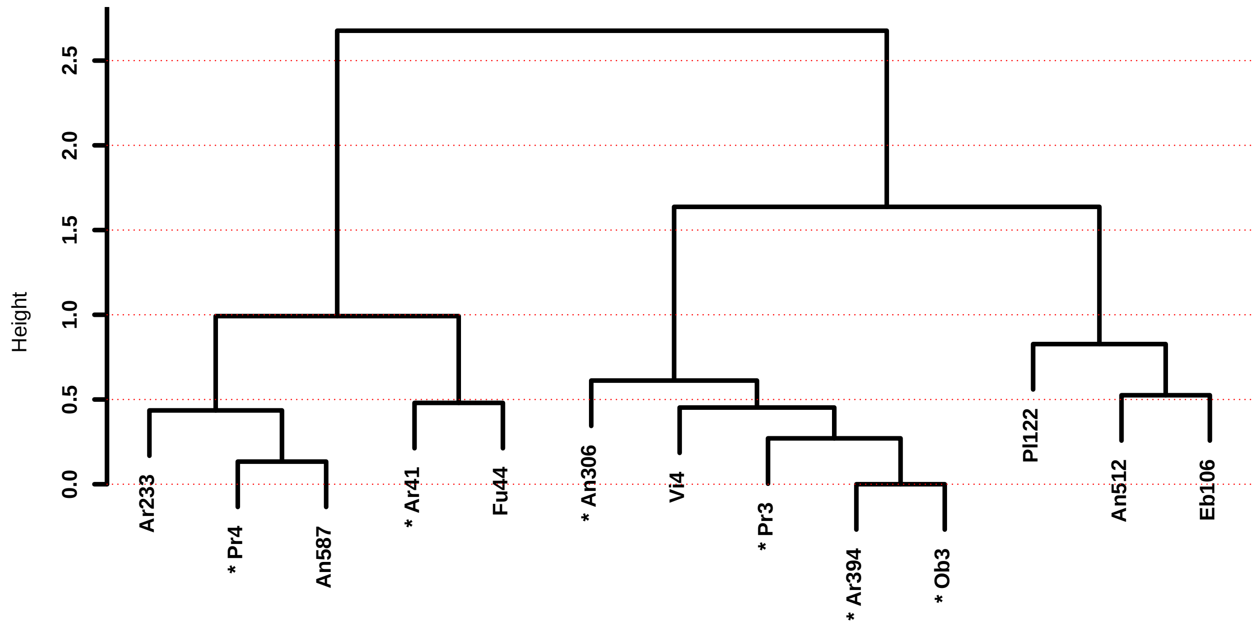

FIGURE **S17**. Dendrogram for the species with larger distances between species pairs in the 64 dimensional space of codon relative frequencies values (humans included only as a reference point). Dendrogram obtained from Euclidean distances by the "complete" method of the function "`hclust()`". Species that also appear in Figure **S16** are remarked by an asterisk in the label.

S5.3. Species close or far to the median per space (H and codon relative frequencies). The median of all the 1,434 values in either, the H or the codon relative frequencies, represents the more reliable central tendency measurement in those spaces –given that the mean is highly affected by outliers. In this section we study the distances of each one of the 1,434 species to the medians in the H and codon relative frequencies spaces.

The H space has 54 dimensions, which correspond to that number of entropy measurements. We formed a vector with the median values of each one of those measurements in the 1,434 species, and then obtained the Euclidean distances that each one of the species has to that vector of medians. The value of that distance for each species tell us if the species is "common" –in the sense of being close to the median, or if it present very peculiar H values (a large distance to the H median) and thus could be considered as an outlier in the H space.

On the other hand, the space of the relative codon frequencies has 64 dimensions, each one corresponding to one codon. Again, we formed a vector with the median values of each one of those measurements in the 1,434 species, and then obtained the Euclidean distances in codon relative frequencies that each one of the species has to that vector of medians. As for H, values, the value of that distance for each species tell us if the species is "common" –in the sense of being close to the median, or if it present very peculiar relative codon frequency values (a large distance to the medians in that space) and thus could be considered as an outlier in the relative codon frequency space.

Table **S18** presents the general statistics for the distances to medians in each one species and in the two spaces, while Figure **S18** presents a dot plot of distances to medians for each one of the 1,434 species in the H (X-axis) and codon relative frequencies (Y-axis) colored by group. Panel (A) presents the whole data for all species while panel (B) shows a zoom view that allows to appreciate details of the bivariate distribution for cases where both distances are relatively small.

In panel (A) of Figure **S18** the bivariate distribution of distances to the medians in the H (X-axis) and codon relative frequencies (Y-axis) presents strong differences for groups. As for other measures,

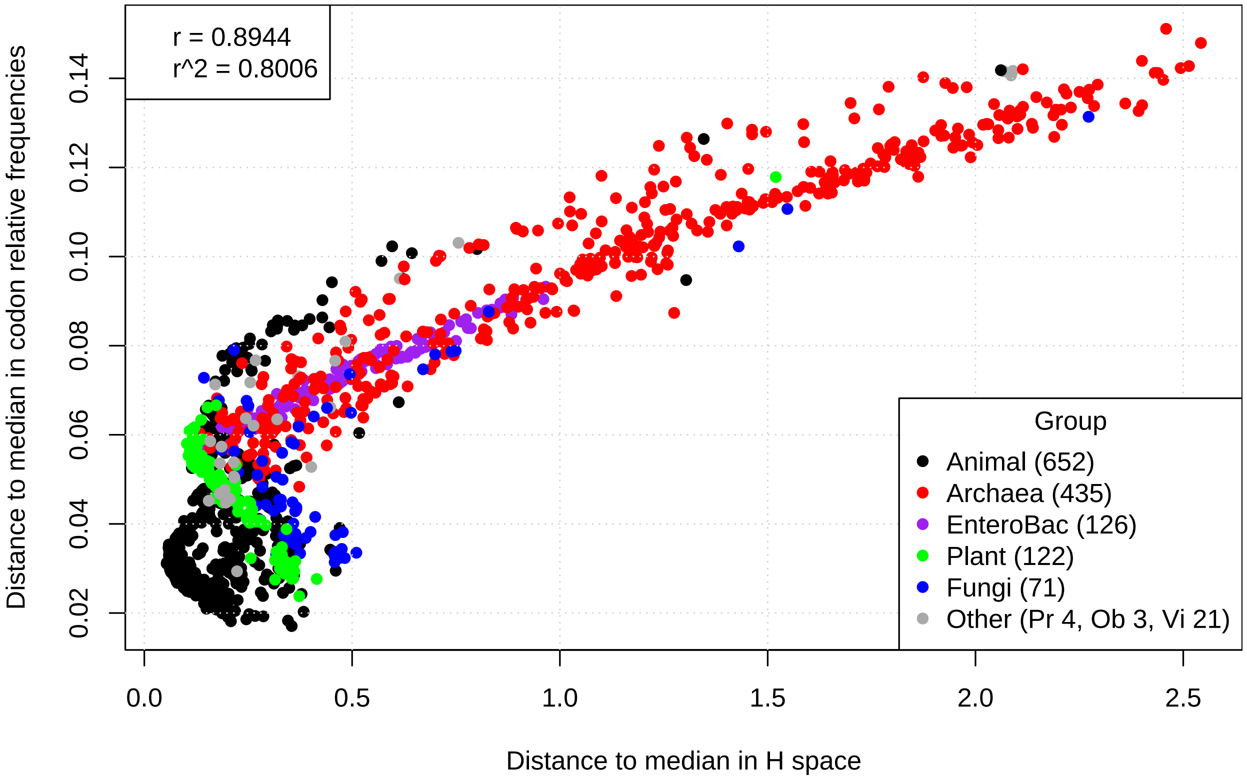

(A) Whole data.

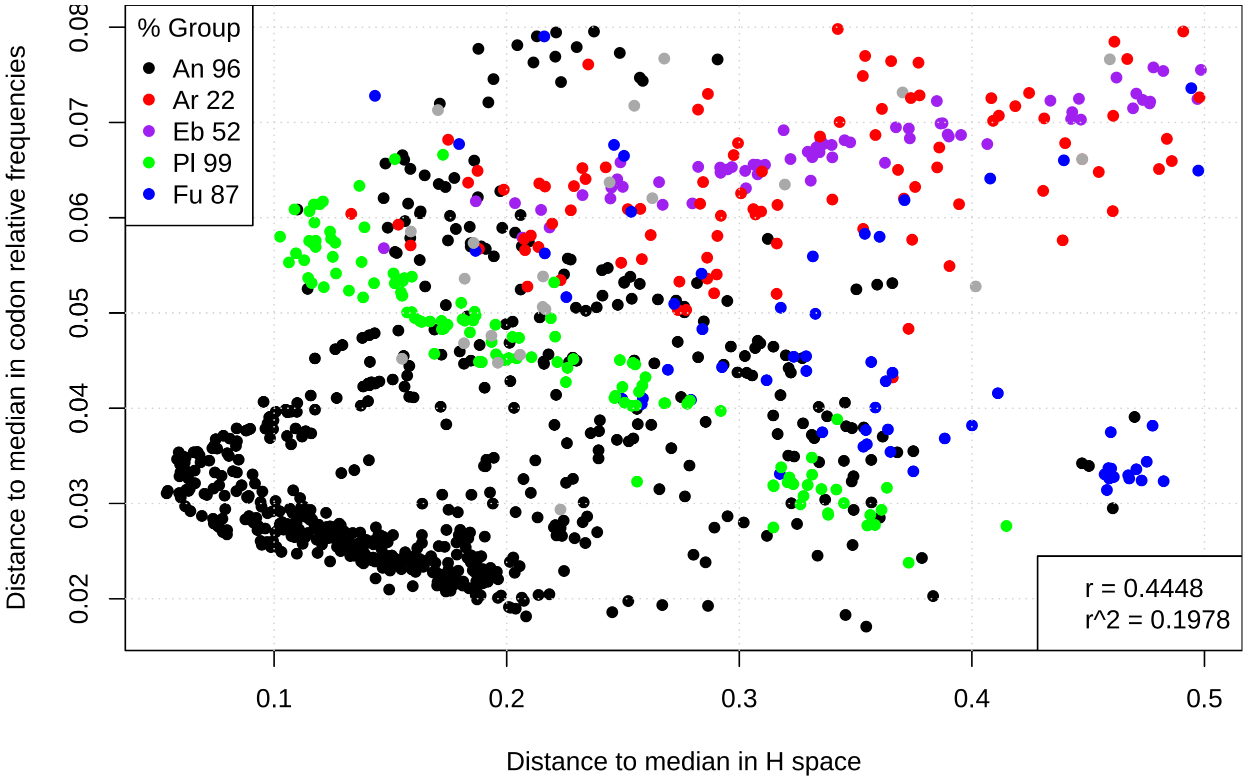

(B) Zoom of the low left hand side corner of panel (A).

FIGURE **S18**. Dot plot of distances to medians for each one of the 1,434 species in the H (X-axis) and codon relative frequencies (Y-axis). See also Table **S18**.

TABLE **S17**. Species shown in the dendrogram of Figure **S16**.

Label	`n.row`	Group	Species	Comment
* An306	310		*Homo sapiens*	* A very strange species indeed!
An512	516	An	*Pollicipes pollicipes*	A species of percebe (goose neck barnacle); ocean organism
An587	591		*Strongyloides ratti*	nematode (parasite) found in wild rats
Ar233	1017		*Methanobrevibacter wolinii* SH	Gram-positive Coccobacillus isolated from sheep faeces
* Ar394	1178	Ar	*Thermococcus barophilus* MP	* piezophilic and hyperthermophilic archaeon
* Ar41	825		*Halarchaeum rubridurum*	* halophilic archaea in the family Halobacteriaceae
Eb106	764	Eb	*Pluralibacter gergoviae*	Gram-negative, motile and facultatively-anaerobic bacteria
Fu44	1264	Fu	*Rhodotorula graminis* WP1	fungi of class Microbotryomycetes, produces red colonies
* Ob3	1219	Ob	*Thermococcus barophilus*	* piezophilic and hyperthermophilic archaeon
Pl122	1413	Pl	*Chlamydomonas reinhardtii*	single-cell green alga, large cup-shaped chloroplast, and an eyespot apparatus
* Pr3	3	Pr	*Entamoeba invadens* IP1	* amoeba parasite of reptiles
* Pr4	4		*Entamoeba nuttalli* P19	* amoeba prevalent in macacos
Vi4	1417	Vi	Human immunodeficiency virus 1	HIV-1 human virus related to viruses in chimpanzees and gorillas

Labels of species beginning with "*" exist also in dendrogram of Figure **S15**.
Group "An" Animal, "Ar" Archaea, "Eb" EnteroBac, "Fu" Fungus, "Ob" are OtherBac, "Pl" Plant, "Pr" Protist, and "Vi" Virus; see Table **S1**.

Archaea (Ar) is the group which presents higher spread in the H space, by having a range of $2.0612 - 0.0538 = 2.0074$ (see Range = Max. - Min. in Table **S18**). However, in the space of the relative codon frequencies, the group of Animal (An) has a larger range than Archaea (Ar), 0.1247 and 0.1079, respectively. Nevertheless, the ample range in Animal is due to a single species, *Strongyloides ratti*, a parasite nematode found in wild rats (black point near the upper right hand side in panel (A) of Figure **S18** which has a value in the X axis of 2.06), while in Archaea there are many (red) points larger than 2.06 in the X axis.

Also in panel (A) of Figure **S18** the estimated Pearson's correlation coefficient between the distances to the medians in all species in the H and codon relative frequencies is high $\hat{r} \approx 0.8944$ ($\hat{r}^2 \approx 0.8006$). Nevertheless this linear tendency is mainly due to the linear pattern observed in Archaea (red points) and occurs primarily in the values above $X > 0.5, Y > 0.08$ while in the lower left hand side of the plot the pattern is very different.

To examine in detail the the low left hand side corner of panel (A), panel (B) in Figure **S18** presents a zoom of the area $X \leq 0.5, Y \leq 0.08$ In panel (B) the legend in the upper right hand side of the plot shows the percentages of points of each one of the main 5 groups, An (Animal), Archaea (Ar), EnteroBac (Eb), Plant (Pl) and Fungi (Fu), which are included in this view. This indicates that large percentages of the Animal (An, 96%), Plants (Pl, 99%) and Fungi (Fu 87%) are included within the zoom view, while only 22% of Archaea (Ar) and 52% of EnteroBac (Eb) are within the limits. Also, the estimated Pearson's correlation coefficient between these points is of only $\hat{r} \approx 0.4448$ ($\hat{r}^2 \approx 0.1978$ lower right hand side legend)

TABLE **S18**. General statistics for the distances to medians for all species in each space.

Distance to general median in the H space.									
	Species group								
Stat. ⇓	All	An	Ar	Eb	Fu	Ob	Pl	Pr	Vi
Min.	0.0538	0.0538	0.1332	0.1472	0.1434	0.4474	0.1026	0.3702	0.1550
Max.	2.5424	2.0612	2.5424	0.9658	2.2721	0.7561	1.5195	2.0899	0.4839
Median	0.2732	0.1598	1.0865	0.4802	0.3630	0.6147	0.1943	2.0771	0.2156
Mean	0.5186	0.1884	1.1178	0.4959	0.4407	0.6061	0.2231	1.6536	0.2473
S	0.5592	0.1348	0.6343	0.1924	0.3163	0.1545	0.1427	0.8556	0.0938
CV	1.0782	0.7152	0.5675	0.3880	0.7178	0.2549	0.6393	0.5174	0.3794
Range	2.4886	2.0074	2.4093	0.8186	2.1288	0.3086	1.4170	1.7197	0.3289
Distance to general median in the space of relative codon frequencies.									
	Species group								
Stat. ⇓	All	An	Ar	Eb	Fu	Ob	Pl	Pr	Vi
Min.	0.0171	0.0171	0.0432	0.0568	0.0314	0.0661	0.0238	0.0731	0.0294
Max.	0.1511	0.1418	0.1511	0.0932	0.1314	0.1031	0.1178	0.1417	0.0809
Median	0.0532	0.0302	0.1001	0.0733	0.0440	0.0951	0.0477	0.1411	0.0538
Mean	0.0608	0.0367	0.0981	0.0736	0.0512	0.0881	0.0467	0.1243	0.0573
S	0.0332	0.0170	0.0252	0.0082	0.0201	0.0194	0.0119	0.0341	0.0130
CV	0.5458	0.4626	0.2564	0.1118	0.3923	0.2206	0.2539	0.2742	0.2269
Range	0.1340	0.1247	0.1079	0.0364	0.1000	0.0369	0.0941	0.0685	0.0516
See Table **S1** for the keys of groups.									

In summary, from Figure **S18** we can conclude that distances to medians for each one of the 1,434 species in the H and codon relative frequencies spaces have a large linear relation mainly for Archaea (red points), but not for the other groups, confirming the fact that entropy measurements in the H space give a very different panorama than the measurements in the relative codon frequencies space.

To study which species have a minimum or a maximum distance to the medians we constructed dendrograms with the species that presented such values either, in the H or the codon relative frequencies spaces. In all the 4 dendrograms of this section (Figures **S19**, **S20**, **S21** and **S22**), additionally to the selected species we presents the **MEDIAN** as a reference. This reference indicates a non-existent species which will have exactly the median for all values in the corresponding spaces. Also, in the beginning of labels of the species we added "**m.**" for the ones that were selected by presenting a minimum distance value, while "**M.**" denotes that the species was selected by presenting a maximum distance value. Species that appear in all 4 dendrograms have an asterisk at the end of the label, and Table **S20** presents the Group, Label, Species (Scientific name) and a brief Comment for all species that appear in the dendrograms.

Figure **S19** for species presenting extreme values of distances with the median in the H space shows 16 species plus the MEDIAN as a reference. In this dendrogram the two clusters linked at a height > 2.5 are formed by 11 species plus the MEDIAN at the left hand side and by 5 species at the right hand side. The cluster at the left hand side links the units at height ≈ 1, and includes only three species selected by the maximum, **M.Eb37 ***, **M.Ob2** and **M.Vi4**; all the other 8 species were selected by presenting a minimum ("**m.**" within their labels). In fact, 6 species are very close to the MEDIAN and are linked in a cluster at height ≈ 0.25; the one closer to the MEDIAN is **m.An378**, a bird with scientific name *Molothrus ater*, followed by **m.Pl198**, the flowering plant *Ricinus communis* (see Table **S20**).

The cluster in the right hand side of the dendrogram in Figure **S19** links 5 species at a height ≈ 1.27 and is constituted by one plant **M.Pl122 ***, *Chlamydomonas reinhardtii*, a single-cell green alga which is conspicuous among plants in both, the H and the relative codon frequencies spaces, an animal, **M.An587 ***, the parasite nematode *Strongyloides ratti*, also conspicuous among animals, and which interestingly is

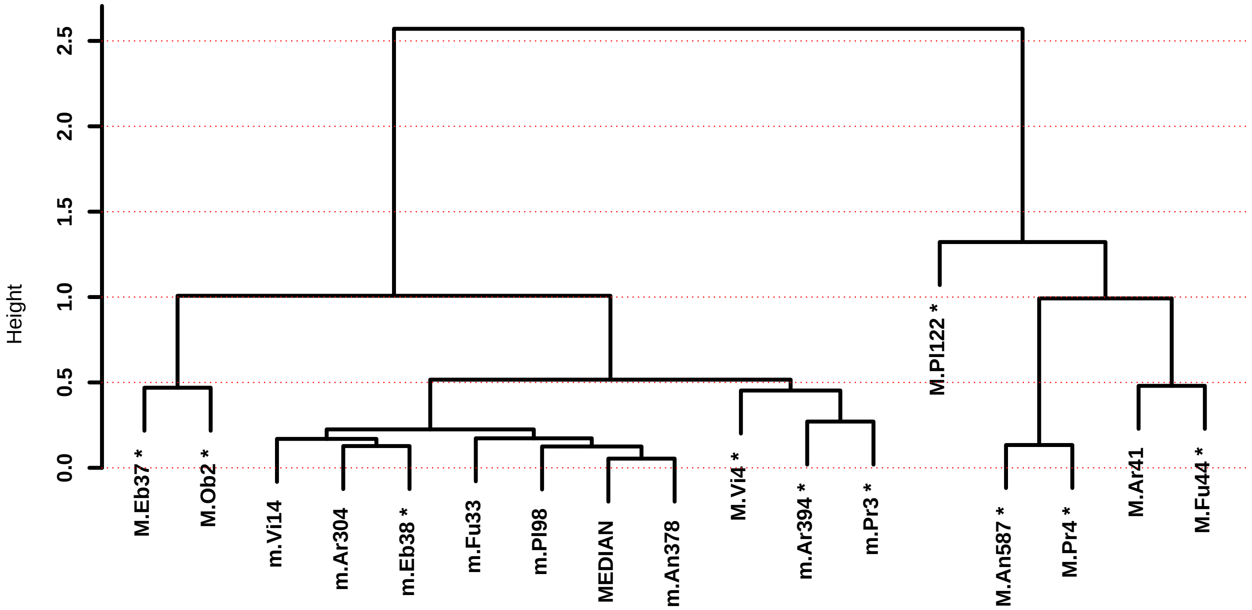

FIGURE **S19**. Dendrogram for the species with extreme values of distance with the median in the H space. The labels for species of each group with showed the **minimum** distance to the median begin with **m.**, while the ones which presented the **Maximum** distance to the median begin with **M.**. The **MEDIAN** is also presented as a reference. Euclidean distance with "complete" method.

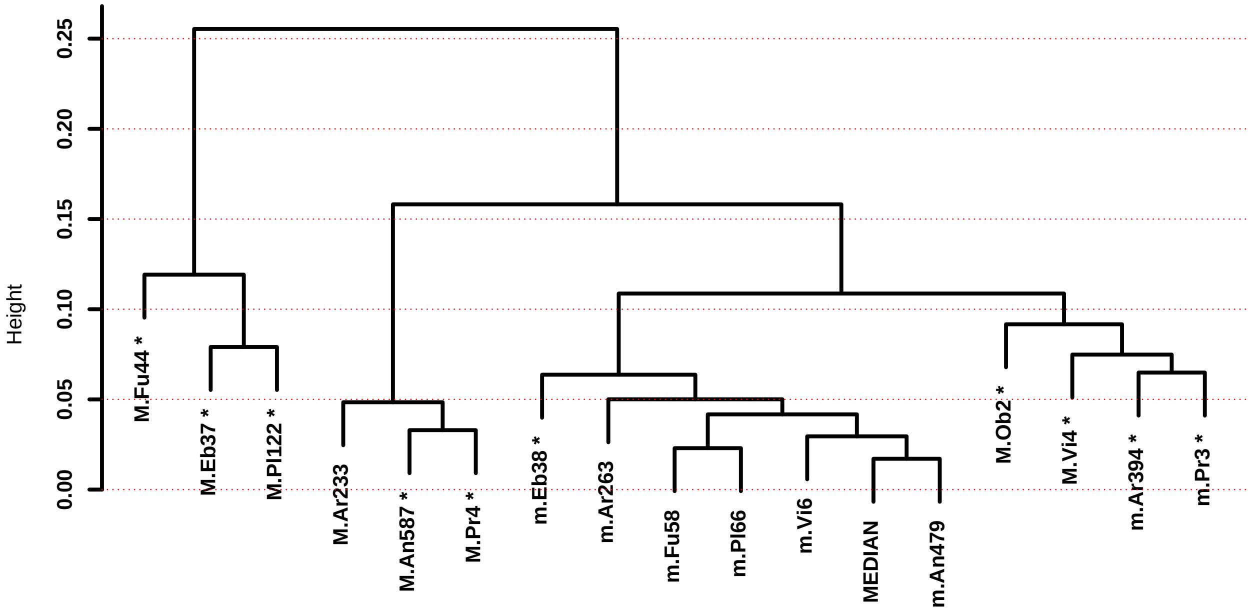

FIGURE **S20**. Dendrogram for the species with extreme values of distance with the median in the space of codon relative frequencies. The convention for labels is as in dendrogram of Figure **S18**. Euclidean distance with "complete" method.

linked in this dendrogram with the amoeba **M.Pr4 *** (*Entamoeba nuttalli*). In this same group there is also an Archaea, **M.Ar41**, and the fungus **M.Fu44 *** of class Microbotryomycetes (see Table **S20**).

In summary, the dendrogram in Figure **S19**, constructed with species which have extreme values of distance to the median in the H space, do not show noticeable taxonomic consistency, but only allows to detect species that are remarkable for their closeness or departure to the median, having representative species for the 8 taxonomic groups.

The dendrogram in Figure **S20** was constructed with the species with extreme values of distance with the median in the space of codon relative frequencies. This dendrogram has 16 species, 8 selected by maximum ("**M.**" in label) and 8 selected by minimum ("**m.**" in label). In total 10 species of the ones shown in Figure **S20** (the ones with an asterisk at the end of the label) are also presented in Figure **S19** –which studied extreme values in the H space. This fact shows that, at least for extreme values, both spaces give relatively concordant results.

Now let's study with more detail exclusively the maximum distances to the median in the H space. Table **S19** presents those distances while Figure **S21** shows the corresponding dendrogram.

TABLE **S19**. Euclidean distances between the **MEDIAN** vector and the 8 species selected by having the largest distance with that vector (one from each group). Employed to estimate the dendrogram in Figure **S21**. Distances vary from 0.1335 up to 2.5424 with a median of 1.2864 The 8 smallest distances are in *italics* annotated with letters a to h.

	**M.An587***	**M.Ar41**	**M.Eb37***	**M.Fu44***	**M.Ob2***	**M.Pl122***	**M.Pr4***	**M.Vi4***
**MEDIAN**	2.0612	2.5424	0.9658	2.2721	*h 0.7561*	1.5195	2.0899	*e 0.4839*
**M.An587***		0.9540	1.2726	*g 0.7368*	1.5264	1.2926	*a 0.1335*	1.6689
**M.Ar41**			1.7262	*d 0.4800*	2.0202	1.2376	0.9922	2.2244
**M.Eb37***				1.4615	*c 0.4690*	0.9334	1.2801	*f 0.6243*
**M.Fu44***					1.8044	1.1492	0.7925	1.9635
**M.Ob2***						1.0794	1.5254	*b 0.4600*
**M.Pl122***							1.3221	1.3343
**M.Pr4***								1.6901

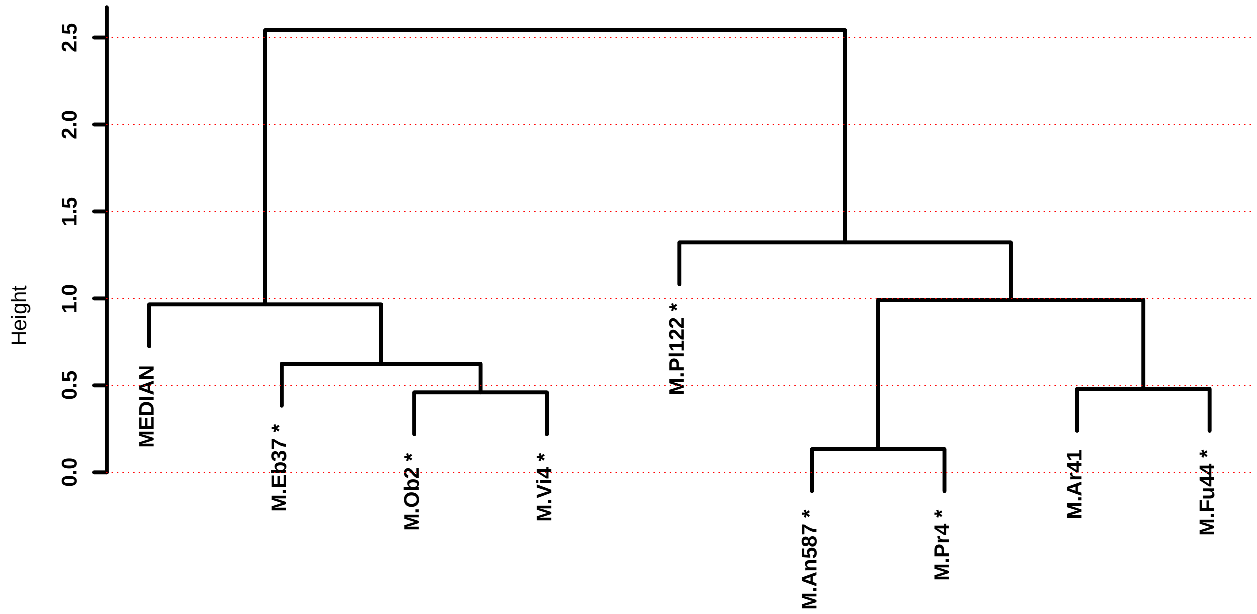

FIGURE **S21**. Dendrogram for the species with maximum values of distance with the median in the H space. Euclidean distances in Table **S19**. Dendrogram with the "complete" method.

Table **S19** is relevant because it presents, for each one of the 8 groups, the species which are more conspicuous in the H space; i.e., the species selected corresponds to the points more to the left hand side

in panel (A) of Figure **S18** (one per group). Figure **S21** gives a dendrogram which has the largest height when considering the H space, because species with maxima distances in that space were selected. In Figure **S21** most (7 of the 8 species –the ones with as asterisk in the label) are also extremes in the relative codon frequencies space; the exception being **M.Ar41** (*Halarchaeum rubridurum*), an halophilic archaea in the family Halobacteriaceae, which in the dendrogram in Figure **S21** is grouped first with **M.Fu44*** (*Rhodotorula graminis*), a fungus of class Microbotryomycetes which produces red colonies. Interestingly, this fungus, **M.Fu44***, presents the second largest Euclidean distance with the **MEDIAN**, the value 2.2721 in Table **S19**, which is only surpassed by the value 2.5424 which corresponds to the already mentioned Archaea **M.Ar41**.

On the other hand, the smallest value in Table **S19**, "*a 0.1335*" occurs between **M.An587*** (*Strongyloides ratti*), a parasitic nematode found in wild rats and the Protist, **M.Pr4*** (*Entamoeba nuttalli*), an amoeba prevalent in macacos. These two species form the binary cluster with the lowest height in Figure **S21**, and the fact that a protist is linked with an animal is not too strange remembering that we are "forcing" the link between outliers of each group (see also panel (B) in Figure **S18**).

Another binary cluster in Figure **S21** is formed between **M.Ob2*** (*Endomicrobium proavitum*) an ultramicrobacterium that fixes nitrogen and **M.Vi4***, the human immunodeficiency virus 1, at height "*b 0.4600*" (Table **S19**) and, again, the fact that a bacteria is linked with a virus is due to the fact that we are detecting the link between outliers of each group.

The representative of plants in Figure **S21**, the single-cell green alga **M.Pl122*** (*Chlamydomonas reinhardtii*) joins at a large height of ≈ 1.35 the cluster already formed by the animal, the protist, the archaea and the fungus. This plant is a clear outlier from the plant group in the H space.

Finally in Figure **S21** the EnteroBac **M.Eb37*** (*Cronobacter universalis*), a gram-negative rod-shaped bacteria, joins the binary cluster formed by **M.Ob2*** and **M.Vi4***, at height > 0.5, showing some taxonomic consistency in the H space.

Figure **S22** presents the dendrogram for the species with minimum values of distance with the median in the H space.

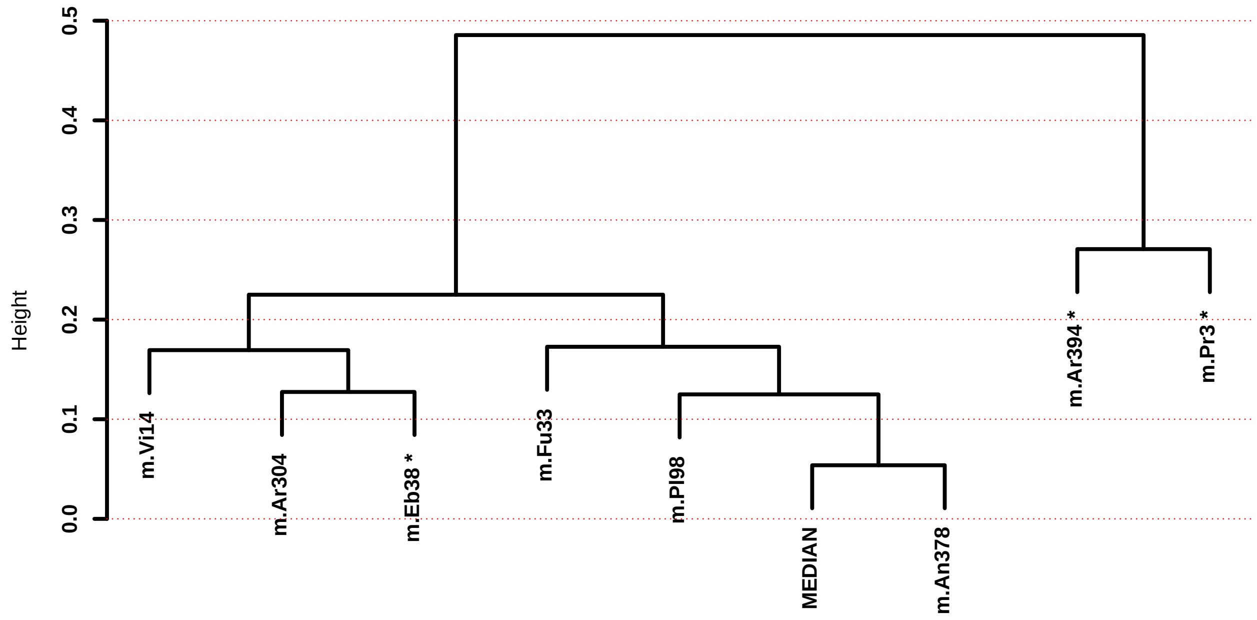

FIGURE **S22**. Dendrogram for the species with minimum values of distance with the median in the H space. Euclidean distance with "complete" method.

Table **S20**. Species shown in the dendrograms presented as Figure **S19** to **S22**.

Group	Label	Species	Comment
Selected by both, H and codon relative frequencies			
An	M.An587 *	*Strongyloides ratti*	nematode (parasite) found in wild rats
Eb	m.Eb38 *	*Edaphovirga cremea*	Gram-negative, facultatively anaerobic, non-motile, nonspore-forming, creamy-pigmented bacterium
Eb	M.Eb37 *	*Cronobacter universalis* NCTC 9529	Gram-negative, facultatively anaerobic, oxidase-negative, catalase-positive, rod-shaped bacteria of the family Enterobacteriaceae.
Fu	M.Fu44 *	*Rhodotorula graminis* WP1	fungus of class Microbotryomycetes, produces red colonies
Ob	m.Ar394 *	*Thermococcus barophilus* MP	piezophilic and hyperthermophilic archaeon (previously classified as bacteria)
Ob	M.Ob2 *	*Endomicrobium proavitum*	ultramicrobacterium; fixes nitrogen
Pl	M.Pl122 *	*Chlamydomonas reinhardtii*	single-cell green alga, large cup-shaped chloroplast, and an eyespot apparatus
Pr	m.Pr3 *	*Entamoeba invadens* IP1	amoeba parasite of reptiles
Pr	M.Pr4 *	*Entamoeba nuttalli* P19	amoeba prevalent in macacos
Vi	M.Vi4 *	Human immunodeficiency virus 1	HIV-1 virus causes human HIV
Selected only by H			
Group	Label	Species	Comment
An	m.An378	*Molothrus ater*	brown-headed cowbird; obligate brood parasite
Ar	m.Ar304	*Methanospirillum lacunae*	methanogenic archaea
Ar	M.Ar41	*Halarchaeum rubridurum*	halophilic archaea in the family Halobacteriaceae
Fu	m.Fu33	*Meira miltonrushii*	mite-associated basidiomycete yeast
Pl	m.Pl98	*Ricinus communis*	Euphorbiaceae; castor bean or castor oil plant, perennial flowering plant
Vi	m.Vi14	Spondweni virus	arbovirus (arthropod-borne virus), family Flaviviridae
Selected only by codon relative frequencies			
Group	Label	Species	Comment
An	m.An479	*Penaeus monodon*	giant tiger prawn and other names, crustacean reared for food
Ar	m.Ar263	*Methanogenium cariaci* JCM 10550	coccoid in shape and Gram-negatives methanogenic archaea
Ar	M.Ar233	*Methanobrevibacter wolinii* SH	species of methanogen archaeon
Fu	m.Fu58	*Suillus paluster*	basidiomycete fungi associated with Pinaceae
Pl	m.Pl66	*Musa acuminata subsp. malaccensis*	a species of banana native to Southern Asia
Vi	m.Vi6	Human mastadenovirus B	human adenovirus

The dendrogram in Figure **S22** is much more compact than the one previously presented in Figure **S21**; the maximum height in the later is 2.5, while in the former it is only 0.5, thus the ratio $2.5/0.5 = 5$ implies that by selecting the species by taking the minima of distances to the median (in Figure **S22**) we obtain a dendrogram that is approximately 5 times less high, or 5 times more “compact”, than when taking the maxima (in Figure **S21**).

Given that we are taking minima distance to the median to select the species in the dendrogram of Figure **S22**, we are selecting species that are more "typical" in the H space. By using curly brackets to represent the topology of that dendrogram, and ignoring the **MEDIAN**, which is there only as reference, we have that the dendrogram's topology is:

$$\{\{\{\text{Vi14}, \{\text{Ar304}, \text{Eb38}\}\}, \{\text{Fu33}, \{\text{Pl98}, \text{An378}\}\}\}, \{\text{Ar394}, \text{Pr3}\}\}$$

thus we have 8 species representing only 7 of the 8 groups (the small group of 3 "Ob" species is absent), and for Archaea (Ar) we have two representatives, Ar304, with label **m.Ar304** which is not present in the dendrogram of Figure **S20** (for the codon relative frequencies space), and Ar394, with label **m.Ar394 *** which is present in the dendrogram of Figure **S20**. The fact of having two Ar in this dendrogram is explained because the range of variation of this group in the H space is so big (see panel (A) in Figure **S18**), that two minima enter in the selection process, forming the binary clusters {Ar304, Eb38} at low height and {Ar394, Pr3} at high height. In words, by taking the minima of distances to the median we find that Ar304 (label **m.Ar304**) a methanogenic archaea (*Methanospirillum lacunae*) is close to Eb38 (label **m.Eb38 ***), a gram-negative bacterium (*Edaphovirga cremea*) which also appears in the dendrogram of Figure **S20**. At the right hand side of the dendrogram in Figure **S22** Ar394 (label **m.Ar394 ***) an archaeon (previously classified as bacteria (*Thermococcus barophilus*) is grouped with an amoeba parasite of reptiles, Pr3 (label **m.Pr3 ***) with scientific name *Entamoeba invadens*. See Table **S20** for the names and comments of other species included in the dendrogram of Figure **S22**.

S6. Relations between GC-content and main entropy measures

Genomic GC-content is a fundamental genomic trait. It has been demonstrated to be surprisingly diverse across the tree of life, and in bacteria, its evolution has been shown to occur through a combination of gradual change and evolutionary jumps (O'Neill et al., 2022). This trait is also strongly linked to other key genomic features, such as codon and amino acid usage.

In this section, we study the relationship between GC-content in the genes of all 1,434 species and our main entropy measures, including $H(\text{codon})$, $H(aa)$, $H(FS)$, $H(FT)$, $H(ST)$, $H(F)$, $H(S)$, and $H(T)$ (see Table **S10**).

The relative frequency of GC-content in the 1,434 species ranged from a minimum of 0.2770 in the Archaea *Methanobrevibacter wolinii* to a maximum of 0.7030 in the single-cell green alga *Chlamydomonas reinhardtii*; both species also exhibited extreme values for the information measures (see Table **S8**). The mean GC-content was 0.5167, with a median of 0.5232 and a standard deviation of approximately 0.09.

Figure **S23** presents box plots for the relative frequency of GC-content across the main taxonomic groups in the study.

As shown in Figure **S23**, Archaea exhibit the widest variation in GC-content among all the groups, with a range from a minimum of 0.2798 to a maximum of 0.6467. This group also presents the largest median (0.6151) and mean (0.5509), along with the largest standard deviation ($S \approx= 0.12$). This confirms that the species within Archaea are the most variable of all the groups studied here (see also Figures **S4**, **S5**, **S7**, **S8** and **S9** for other box plots). Notably, the interquartile range for Archaea includes the interquartile ranges of the other four groups.

From Figure **S23**, we also observe that the mean GC-content is greater than 0.5 for Archaea, EnteroBac, and Fungi, while for Plants, the mean is significantly lower at 0.4580. This suggests that the evolution of plant genes may have been subject to less selective pressure for a high GC-content. On the other hand, the GC-content for animals, with a mean of 0.4987, is very close to the neutral value of 0.5. The two animal species closest to this value are the turtle *Terrapene carolina triunguis* and the fish Cyprinus carpio, which have GC-contents of 0.4999 and 0.5002, respectively.

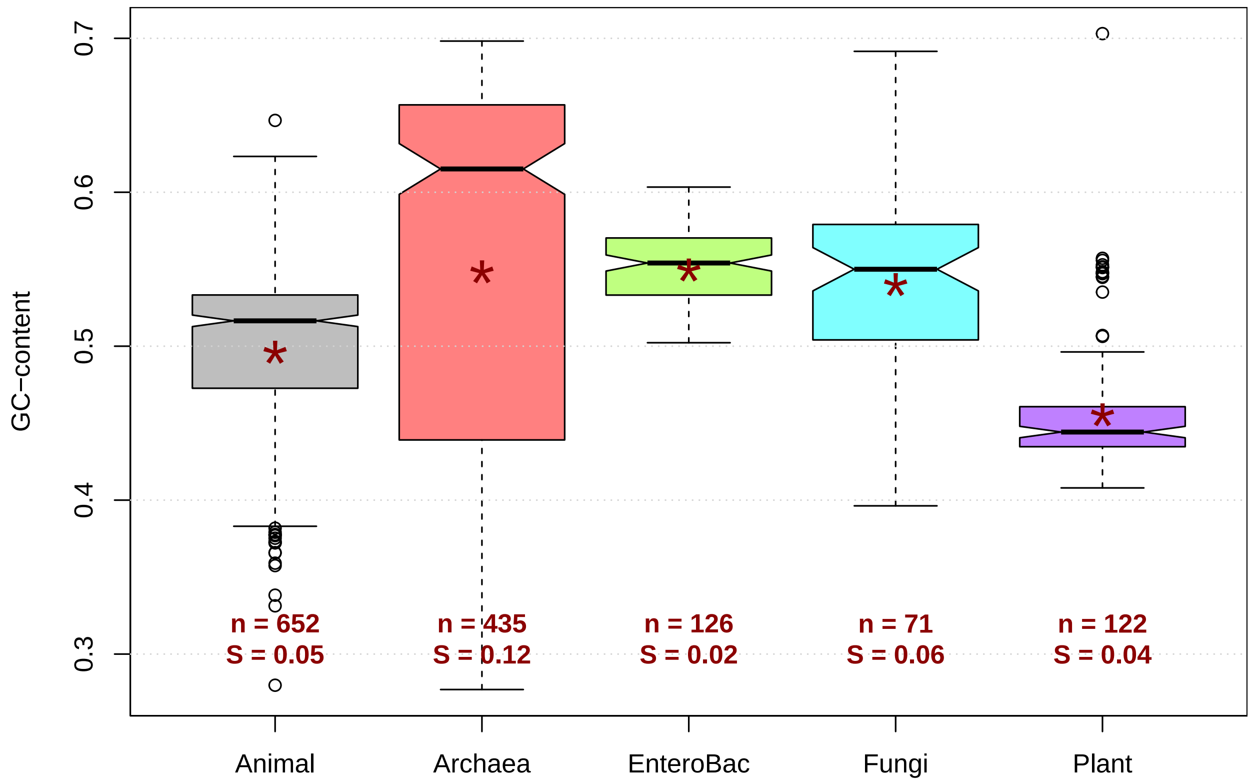

FIGURE **S23**. Box plots for GC-content in the main groups of species studied. Means plotted as asterisks; n are number of species and S standard deviation in each box plot.

In conclusion, the box plots in Figure **S23** demonstrate that GC-content exhibits ample variation both within and between the taxonomic groups studied. With this in mind, we next examine the relationship between GC-content and our informational parameters. To this aim, Figures **S24** to **S27** present dot plots of GC-content versus the entropy measures, with data points colored by their group of origin.

GC-content is a fundamental genomic trait linked to many key genomic features, such as codon and amino acid usage. This trait is well-understood and widely used by scholars in the field to provide a concise molecular summary of genetic code usage for each species. The question is whether our entropy measures are merely simple variants of GC-content or if, in contrast, the study of these informational properties adds a deeper understanding of the molecular grammar latent in the genetic code.

Figures **S24** to **S27** present dot plots of GC-content versus the main entropy measures, with data points colored by their group of origin. These plots show that the relationships between GC-content and each of the main entropy measures are complex and far from linear. In all eight panels of the four figures, it is easy to appreciate that the entropy measures (on the Y-axis) better segregate the species into their corresponding groups than the GC-content (on the X-axis). In other words, there is less overlapping between groups along the Y-axis compared to the X-axis. This implies that entropy measures are, in general, more informative regarding the group of origin of the species than the GC-content.

In more detail, panel (A) of Figure **S24** shows the relationship between GC-content and H(codon), where we find a curvilinear relationship. The maximum value of H(codon) occurs at a GC-content of approximately 0.5 (the neutral value), while the minimum values of H(codon) are found at both tails of the GC-content distribution. The estimated Pearson's correlation coefficient is negative, $r = -0.48250$, a value that was strongly influenced by the large number of Archaea data points located in the region where $X > 0.6$. Curvilinear relationships similar to the one in panel (A) of Figure **S24** are also present in panel (B) of Figure **S24**, panel (A) of Figure **S26**, and panel (B) of Figure **S27**. In all these cases, if one were to guess a species' group of origin based on its GC-content (X-axis) versus its entropy value

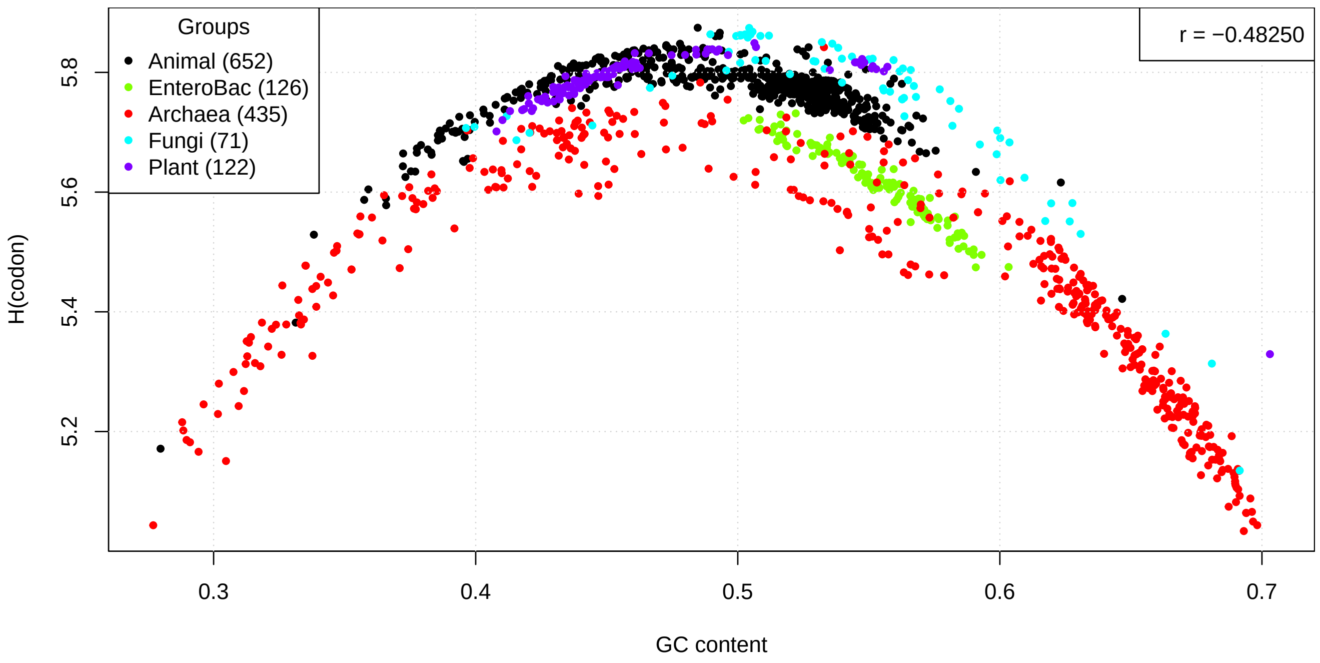

(A) GC-content by entropy given by codons, H(codon).

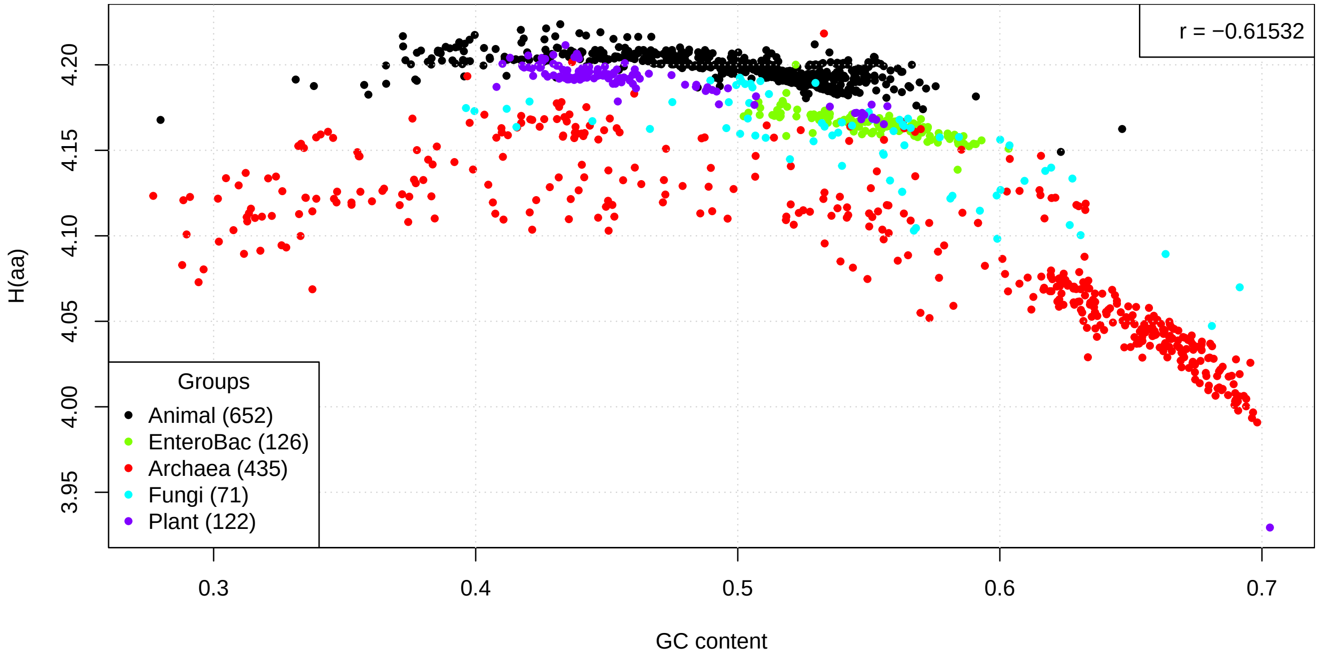

(B) GC-content by entropy given by amino acids, $H(aa)$.

FIGURE **S24**. Dot plots of GC-content with entropies; data points colored by their group of origin (1 of 4 figures).

(Y-axis), the guess would be more likely to be correct with the entropy value. This demonstrates that entropy values are in no way redundant when GC-content values are known.

Interestingly, the only case where there is a strong linear relationship between GC-content and an entropy measure occurs for the entropy of the second base, $H(S)$, presented in panel (A) of Figure **S27**, which shows a correlation of $r = 0.82154$ ($r^2 = 0.6749$).

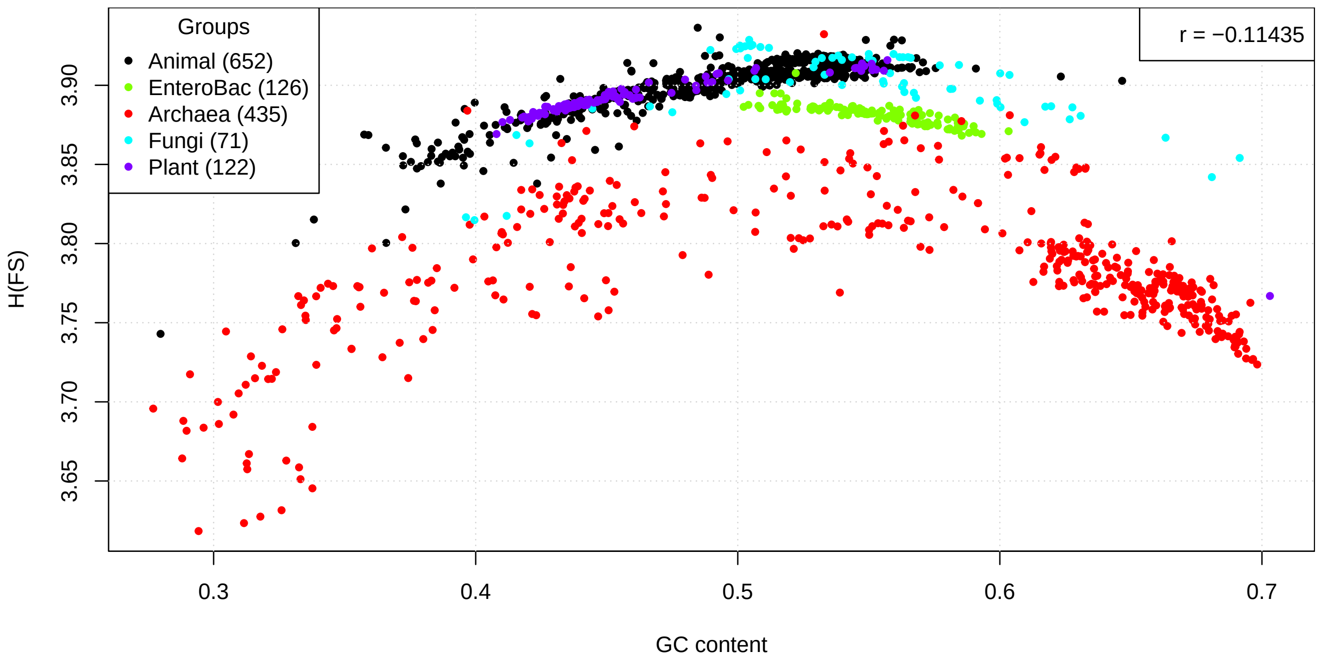

(A) GC-content by entropy given by First-Second bases, $H(FS)$.

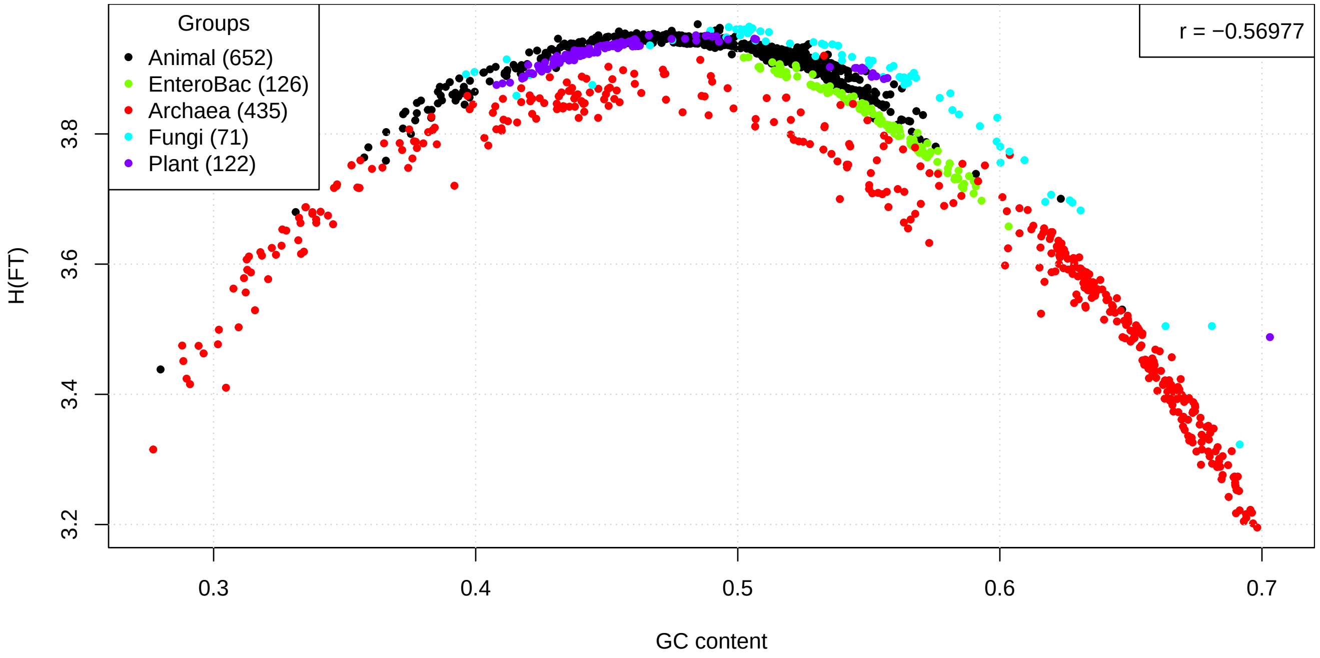

(B) GC-content by entropy given by First-Third bases, $H(FT)$.

FIGURE **S25**. Dot plots of GC-content with entropies; data points colored by their group of origin (2 of 4 figures).

In conclusion, our analysis of the relations between GC-content and the main entropy measures (as presented in the "Single" row of Table **S10**) shows that these entropy measures provide valuable information that cannot be obtained directly or indirectly from GC-content alone. The same conclusion is reached by examining the other 46 informational measures, which correspond to Joint Entropies (6 cases), Conditional Entropies (25 cases), and Mutual Informations (15 cases); these results are also summarized in Table **S10**, but plots are not shown here for brevity.

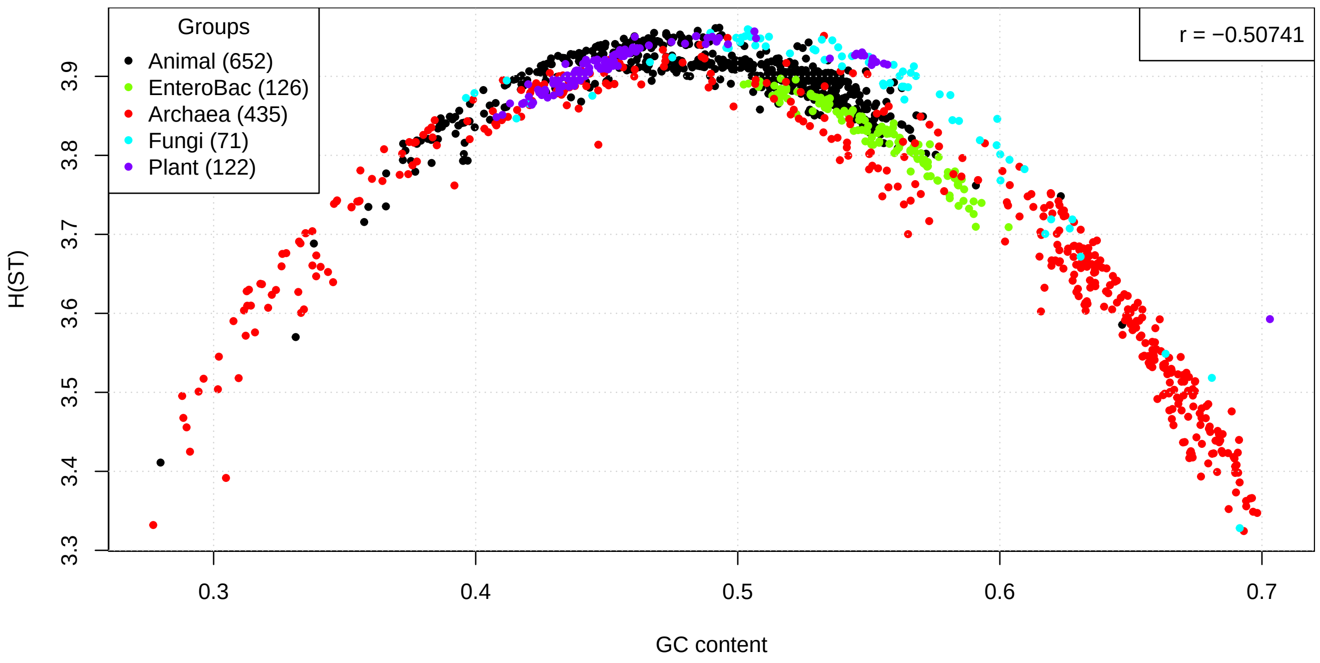

(A) GC-content by entropy given by Second-Third bases, $H(ST)$.

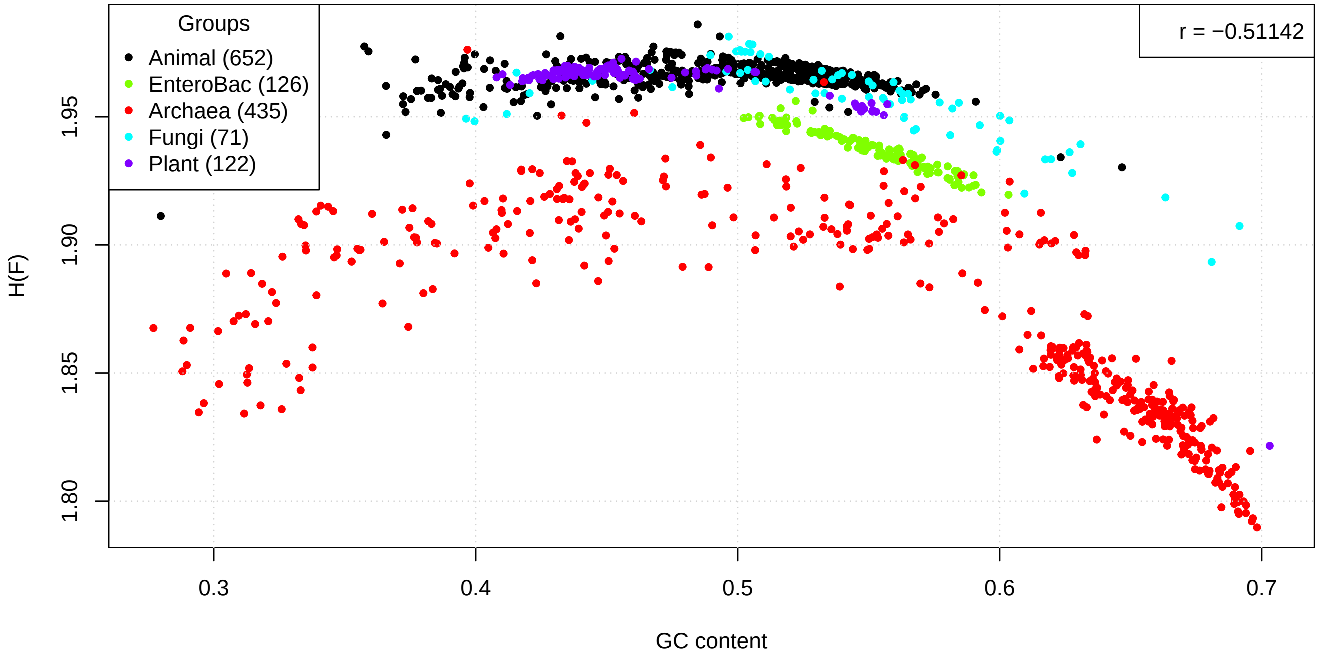

(B) GC-content by entropy given by First base, $H(F)$.

FIGURE **S26**. Dot plots of GC-content with entropies; data points colored by their group of origin (3 of 4 figures).

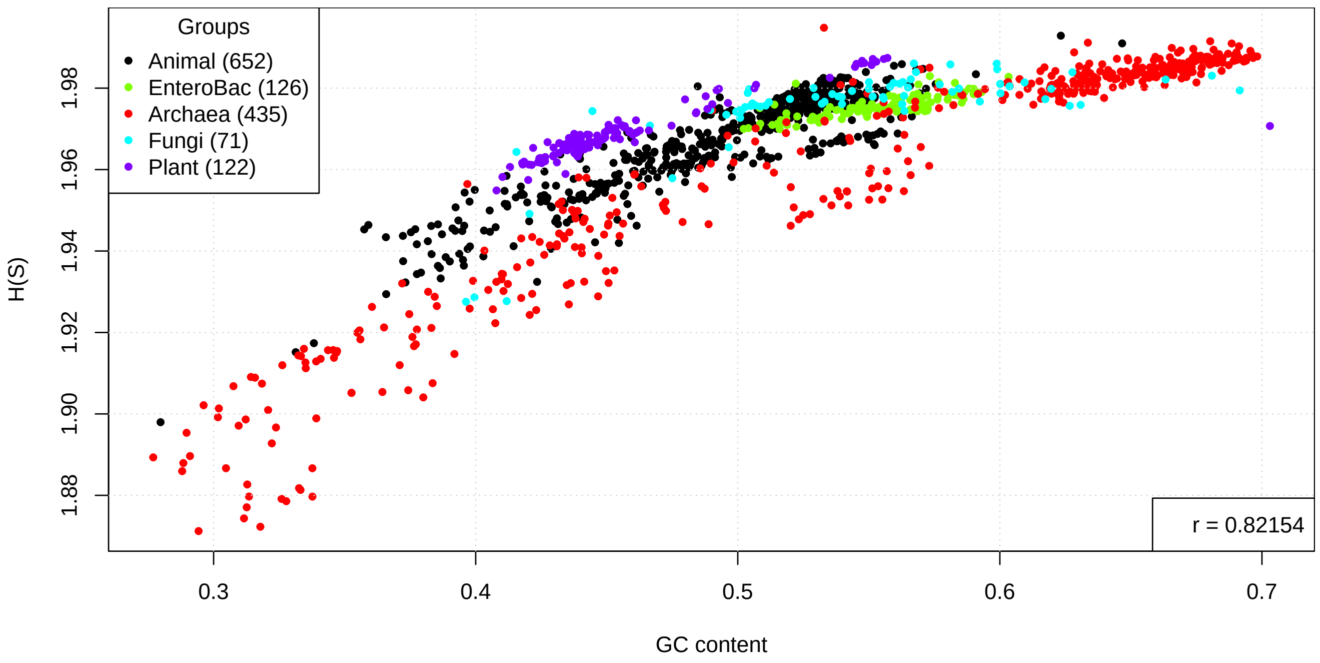

(A) GC-content by entropy given by Second base, $H(S)$.

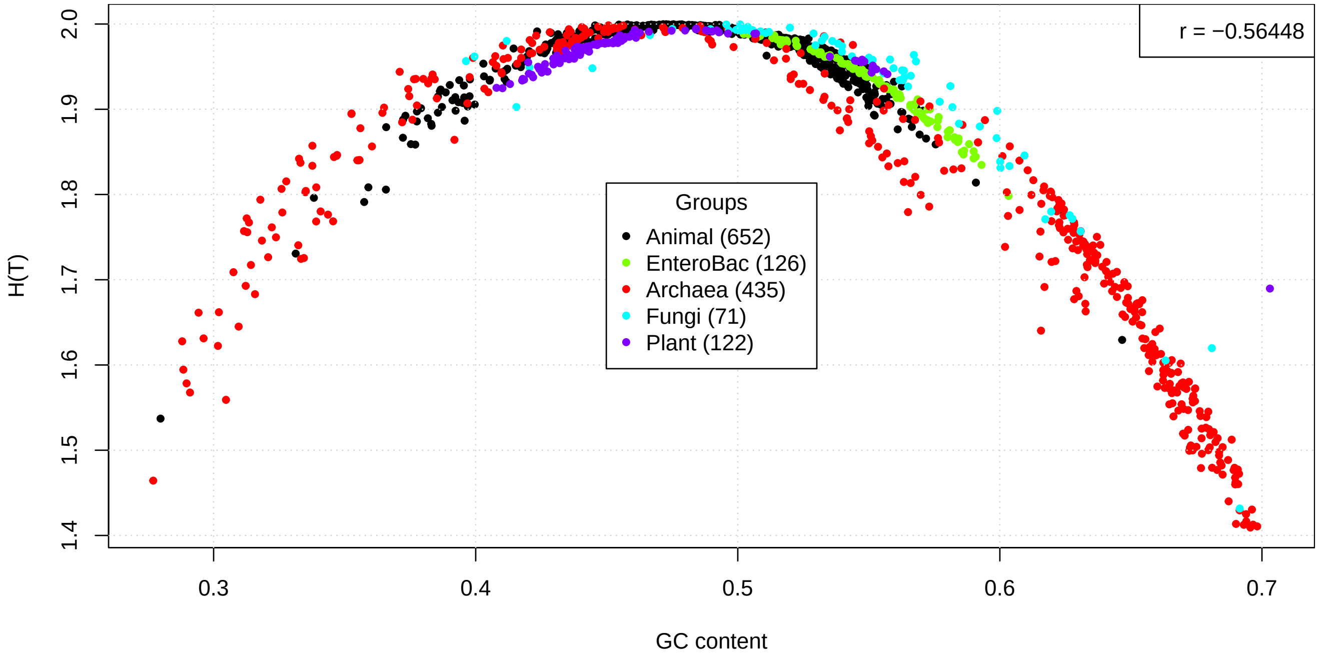

(B) GC-content by entropy given by Third base, $H(T)$.

FIGURE **S27**. Dot plots of GC-content with entropies; data points colored by their group of origin (4 of 4 figures).

References

Adami C (2004) Information theory in molecular biology. *Physics of Life Reviews*, 1, 3–22.

Adami C (2016) What is information? *Philosophical Transactions of the Royal Society A: Mathematical, Physical and Engineering Sciences*, 374, 20150230.

Adami C (2024) *The Evolution of Biological Information: How Evolution Creates Complexity, from Viruses to Brains.* Princeton University Press.

Martínez O (2025) Shannon.codon: An R package for the analysis of codon frequencies. *Zenodo submission.* doi:10.5281/zenodo.15650136. URL `https://doi.org/10.5281/zenodo.15650136`.

Martínez O (2024) Dendrolikeness: an R package to compare dendrograms. *Zenodo submission.* doi: 10.5281/zenodo.13737570. URL `https://doi.org/10.5281/zenodo.13737570`.

O'Neill RM, Smith SA, Johnson AD, O'Meara GWL, and Smith SA (2022) Evolutionary jumps in bacterial gc content. *G3: Genes—Genomes—Genetics*, 12. doi:10.1093/g3journal/jkac108. URL `https://academic.oup.com/g3journal/article/12/8/jkac108/6586800`.

R Core Team (2013) *R: A language and environment for statistical computing.* R Foundation for Statistical Computing, Vienna, Austria. URL `http://www.r-project.org`.

Shannon CE (1948) A mathematical theory of communication. *The Bell System Technical Journal*, 27, 379–423.

Subramanian K, Payne B, Feyertag F, and Alvarez-Ponce D (2022) The codon statistics database: a database of codon usage bias. *Molecular Biology and Evolution*, 39, msac157.

S7. Appendix: R objects containing results.

Tables **S21**, **S22** and **S23** show the R objects obtained during the analyses. The R binary file

"`SuppResCodonUse.RData`"

contains such objects and is available upon request from the corresponding author.

Table **S21**. Relevant R objects

**Object**	**Comment**
all.H.table	Table with categories of all.H results
col4group	Colors for group (for plots)
common.spe.fin.den	Common species in final dendrogram (den.finalHmat$labels)
common.spe.lab.fin	Labels in substring(common.spe.fin.den, 3, 15)
comp.clu.Hs.cod.fre	Comparison of clusters H and codon relative frequencies
comp.clu.Sl0	Comparison of shared clusters between dendrograms obtained from Hs and codon relative frequencies; see 58_Results, Mon May 26 08:02:45 2025.
d2d.all	Distances between pairs of all species; dis.Hs - Distances in the space of the 54 H values, dis.cf - Distances in the space of the 64 relative codon frequencies.
d2d.all.stats	Statistics for all distances between species
d2d.all.stats.all	Summary of statistics for all distances between species
d2d.BetAr	Distances between Archaea and other groups; dis.Hs - Distances in the space of the 54 H values, dis.cf - Distances in the space of the 64 relative codon frequencies. Used for BoxPlotDisWithAr.pdf
den.avg.dis.mainH	hclust(dis.mainH, method=average)
den.avg.mainHgroup	hclust(dis.median.mainH, method=average)
den.avg.mean.crf	hclust(w.mean.dis.crf, method=average)
den.avg.median.crf	hclust(w.median.dis.crf, method=average)
den.avg.w.mean.mainH	hclust(w.mean.dis.mainH, method=average)
den.avg.w.median.mainH	hclust(w.median.dis.mainH, method=average)
den.com.allIs	hclust(dis.all.Is)
den.com.dis.mainH	hclust(dis.mainH, method=complete)
den.com.dis.median.Is	hclust(dis.medians.Is)
den.com.mainHgroup	hclust(dis.median.mainH, method=complete)
den.com.mean.crf	hclust(w.mean.dis.crf, method=complete)
den.com.median.crf	hclust(w.median.dis.crf, method=complete)
den.com.w.mean.mainH	hclust(w.mean.dis.mainH, method=complete)
den.com.w.median.mainH	hclust(w.median.dis.mainH, method=complete)
den.final.crf	hclust(dist(final.crf.mat))
den.final.Hmatm	hclust(dist(finalHmat.M))
den.final.HmatM	hclust(dist(finalHmat.m))
den.finalHmat	hclust(dist(finalHmat))
den.max.dis.cf	hclust(dis.max.dis.cf)
den.max.dis.H	hclust(dis.max.dis.H)
dis.all.Is	Euclidean distances between all mutual information terms (Is, denoted only by their terms separated by a full stop)
dis.all.species.all.Hs	Euclidean distances between species in the full space of the 54 H measurements.
dis.cod.rel.fre	Euclidean distance between all species from relative codon frequencies
dis.mainH	1-as.dist(cor(species.H.all[, 1:8])ˆ 2)

Table **S22**. Relevant R objects (continues from Table **S21**)

**Object**	**Comment**
dis.max.dis.cf	dis.max.dis.cf <- dist(spec.max.dis.cf.dat)
dis.max.dis.H	dist(spec.max.dis.H.dat)
dis.mean.mainH	dist(mat.mean.mainH)
dis.median.mainH	dist(mat.median.mainH)
dis.medians.Is	dist(t(mat.med.Is.group))
dis.r2Hs.raw	as.dist(cor(species.H.all[, 1:8])^ 2)
dist2data	Function to convert a distance matrix to a data.frame
dist2median	Distances to median for each species in both spaces
dist2median.crf	dist2median.crf[i] <- sqrt(sum((codon.rel.freq[i,]-median.crf)^ 2))
dist2medianHs	dist2medianHs[i] <- sqrt(sum((species.H.all[i,]-median.Hs)^ 2))
extreme.spH2	Extreme species in H, 2 by group
extreme2median.crf	Extreme species in codon relative frequencies per group
extreme2medianH	Extreme species in H space per group
final.crf.mat	Matrix for final dendrogram of species by codon rel. freq.
finalHmat	Matrix for final dendrogram of species by H
finalHmat.m	Matrix of minima for final dendrogram in H
finalHmat.M	Matrix of maxima for final dendrogram in H
groups.lab	sort(unique(substring(species.real.label, 1, 2)))
huge	topo.den.com.allSpeAllHs <- dend.topo(den.com.allSpeAllHs)
huge.amp.H.all	Data of all H for all species to calculate distances
huge.cla2	For clase 2
huge.clu	huge$clusters
huge.cons.class	Consistency in huge cluster (H space)
huge.den.amp	huge.den.amp <- hclust(huge.dis.amp)
huge.den.com.sel.cla2	hclust(huge.dis.sel.cla2)
huge.dis.amp	huge.dis.amp <- dist(huge.amp.H.all)
huge.dis.sel.cla2	huge.dis.sel.cla2 <- dist(huge.sel.cla2.H.all)
huge.hei.by.class	Height in huge dendrogram by class
huge.ori	huge$original
huge.sel.cla2	Selected from huge by class
huge.sel.cla2.H.all	Selecte in H space per class
huge.sel.cla2.spe	union(huge.sel.cla2$spe1, huge.sel.cla2$spe2)
huge.spe.sel.cla2	table of species using species2
huge.speExtraF13	Label, group and Species
huge.tab.cla2	table(huge.cla2$group); huge.tab.cla2[order(huge.tab.cla2, decreasing=T)]
huge.tab.class	huge.tab.class <- table(huge.ori$class)
huge.tab.square.class	huge.tab.square.class <- table(huge.tab.class)
lm4Is	lm(species.std.I$s.I.aa.FS[species.std.I$present==T] ~ species.std.I$s.I.aa.S[species.std.I$present==T])
mat.mean.mainH	Matrix of means of main H per group
mat.med.Is.group	Matrix of medians per group of all the Is in species.
mat.median.mainH	Matrix of medians of main H per group
median.crf	apply(codon.rel.freq, 2, median)
median.Hs	apply(species.H.all, 2, median)
min4H2	Minima for H (2)
objects.comment	Comments for relevant objects

TABLE **S23**. Relevant R objects (continues from Table **S22**)

**Object**	**Comment**
pairs.max.dis.cf	Distances between pairs of all species; dis.Hs - Distances in the space of the 54 H values, dis.cf - Distances in the space of the 64 relative codon frequencies.
pairs.max.dis.H	Distances between pairs of all species; dis.Hs - Distances in the space of the 54 H values, dis.cf - Distances in the space of the 64 relative codon frequencies.
rel.fb	Cases are number of times that First base is present in the coding of the amino acid; column NdifAA gives the number of different aa coded by each instance of the first base.
rel.tb	Cases are number of times that Third base is present in the coding of the amino acid; column NdifAA gives the number of different aa coded by each instance of the Third base.
res.H1	Table per group of species
spe.den.final.all	A file with all species
spe.den.final.crf	data.frame tabulating the Species which appear in final dendrogram by codon frequencies
spe.den.finalHmat	data.frame tabulating the Species which appear in final dendrogram by H
spe.group	Species per group
spec.ind	a vector of indicators of the species
spec.max.dis.cf	Species with maximum distance in codon frequencies
spec.max.dis.cf.dat	Relative codon frequencies for species selected by presenting the largest distances in pairs of codon frequencies, plus human as reference
spec.max.dis.H	Species with maximum distance in H
spec.max.dis.H.dat	Data for the species with maximum distance in H
species.all.Is	All Is for all species (mutual informations)
species.H.all	data.frame with the results of H.all() for each one of the 1434 species. Names are simplified. Row names are the labels that can be used for analyses of each one of the 8 taxonomic groups (the first two characters define the group)
species.huge.amp	attributes(huge.amp.H.all)$row.names
species.Hy1	Data testing hypothesis 1 H.T $\geq$ H.F
species.Hy2	Data testing hypothesis 2: H.F.g.aa, H.T.g.aa, difTminF
species.Iaa	cbind(species.Hy2[,1:3], species.H.all[,40:45])
species.only.aa.I	Only aa Is (mutual informations)
species.real.label	The true (updated) label for each specie, attributes(species.H.all)$row.names
species.std.I	Standardized mutual information terms (sI) and auxiliar variables
species2	An updated data.frame like species but with the real.label
sta.8H	Standardized main Hs (singleH)
sta.dist2median.to.crf	Statistics of distances to median in codon relative frequencies
sta.dist2median.toHs	Statistics of distances to median in H
sum.dis.crf	Summary of Euclidean distances between relative frequencies of the 64 codons within and between group (set)
sum.dis.mainH	Summary of Euclidean distances mainH (8 elements) within and between group (set)
sum.m.dis.crf	Matrix with summary of distances in codon relative frequencies
sum.m.dis.mainH	Matrix with summary of distances in H
summ.H2	Summary of H2
tab.all.dis.sum	Table StatsAllDis.txt (a table with a nice summary of results)
table.sel.spe	Table with selected species
table2	Table 2; T2medXgroup.txt
table9	Table of maxima; table9.txt
w.mean.dis.crf	Mean of distances in codon relative frequencies
w.mean.dis.mainH	Mean of distances in H
w.median.dis.crf	Median of distances in codon relative frequencies
w.median.dis.mainH	Median of distances in H
forGCanal	Data for analyses of GC-content
